# Mind the gap: An embedding guide to safely travel in sequence space

**DOI:** 10.1371/journal.pcbi.1014433

**Published:** 2026-07-09

**Authors:** Adam Wu, Jakub Lála, Quentin Trolliet, Abhinav Rajendran, Stefano Angioletti-Uberti

**Affiliations:** 1 AminoAnalytica Ltd, Bedford, United Kingdom; 2 Department of Materials, Imperial College London, London, United Kingdom; 3 Thomas Young Centre, Imperial College London, London, United Kingdom; 4 Nanograb Ltd, London, United Kingdom; Sorbonne Universite, FRANCE

## Abstract

We present a hybrid approach combining a protein language model (pLM) with Monte Carlo (MC) sampling for generating enzyme mutants free of mutations deleterious for structural preservation. Given the amino acid sequence of the original enzyme and a set of residues for which the local environment should be conserved, i.e., the catalytic site, our approach generates mutants that differ vastly in the overall sequence while retaining the geometry of the conserved region, thereby representing promising candidates for further experimental screening. Unlike end-to-end deep-learning approaches, whose results are harder to interpret and control, the use of a well-established, classic technique such as MC sampling allows us to easily interpret the generative process as the sampling of an energy landscape determined by the pLM. In turn, such an interpretation enables us to steer this generative process and control its outcome by making use of robust statistical mechanics concepts, e.g., temperature, thereby explicitly guaranteeing certain properties of the generated mutants. We further show, through comparison to experimentally characterised chorismate mutase variants, that low embedding energy is a necessary condition for catalytic function, providing direct experimental grounding for the energy function at the core of our approach. Given the increasing relevance of generative algorithms in the design and search for novel, optimised enzymes, we believe that our results constitute an important step for the future development of this class of techniques. To facilitate experimental verification, we finally provide over 12,500 sequences in total for 13 different enzymes involved in catalytic processes ranging from biomass degradation to DNA replication.

## Author summary

Enzymes are nature’s catalysts: proteins that accelerate essential chemical reactions. Engineers often want to redesign them, but even a single amino acid change can destroy function by disrupting the precisely shaped active site where catalysis occurs. We present a method that generates enzyme sequences potentially very different from the original while reliably preserving the active-site geometry. It combines a protein language model (an AI trained on millions of protein sequences) with Monte Carlo sampling, a well-understood statistical technique. The algorithm proposes random mutations one at a time, and the protein language model scores these mutations via an energy. The higher the energy, the more the active site environment is perturbed, and the algorithm rejects those mutations that disturb it excessively. Unlike black-box AI approaches, the method is interpretable: it behaves like a physical system with a tuneable “temperature” controlling how boldly it explores. We validated it against experimental data, confirming that a low-energy score is a necessary (but not sufficient!) condition for the enzyme to remain functional, and used molecular dynamics simulations to verify that generated enzymes remain structurally stable. Over 12,500 candidate sequences across 13 enzymes are provided as an open resource for experimental testing.

## 1 Introduction

Enzymes are protein catalysts that enable specific chemical reactions and are fundamental to numerous industrial and therapeutic applications, from biofuel production to pharmaceutical synthesis. Yet, the discovery and optimisation of enzymes with desired properties, such as enhanced activity, stability, or substrate specificity, remain a significant bottleneck in enzyme engineering [1]. The current gold standard, directed evolution [2,3], is an iterative process that mimics natural selection by creating diverse libraries of enzyme variants through random mutagenesis or recombination, followed by high-throughput screening or selection for the desired trait. Although directed evolution has produced numerous successful enzymes, it is inherently limited by the vastness of sequence space [1]. Experimentally probing this space is time-consuming and labour-intensive, and requires careful decisions about the initial sequence library. For example, one’s starting point might be enzymes from bacteria or plants that evolved in environments similar to those desired in the application. It is clear that in use cases with limited structural or mechanistic information, the challenge is even greater. One notable success story of directed evolution has been the discovery and optimisation of Taq polymerase, a thermostable DNA polymerase that enabled the development of polymerase chain reaction (PCR) technology. Its discovery involved isolating the native enzyme from extremophilic bacteria that lived in the prohibitively hot springs of Yellowstone Park, where high temperatures caused the evolution of heat resistance necessary for PCR [4]. Today, Taq polymerase is widely used for a wealth of biotechnology applications, from medical diagnostics to quality control in food production [5].

When employing random mutagenesis strategies in directed evolution, a major limitation is often the high probability of introducing deleterious mutations: the intricate three-dimensional structure of an enzyme is crucial for its function and catalytic activity, and even a single amino acid substitution can disrupt this delicate architecture [6]. Mutations that destabilise the structure of the active site (also referred to as the catalytic site throughout this work) are overwhelmingly likely to abolish or severely impair catalytic activity. Compounding this issue is the fact that functionally important residues are not solely confined to those close to the active site itself. Distant residues, both in terms of the primary sequence or of the three-dimensional structure, can still play a crucial role in determining the overall fold of the enzyme and thus maintaining the structural integrity of the active site. Therefore, a significant proportion of the mutants generated in directed evolution through random amino acid changes can lead to misfolding of the active site and loss of activity, representing wasted experimental effort and impeding the efficient exploration of functional enzyme space.

In recent years, the rapid advancement of artificial intelligence (AI) and the development of powerful protein language models (pLMs) [7–10] have opened new avenues for protein engineering and enzyme design [11–14]. In this regard, most AI-based generative approaches in the literature use a one-shot strategy based on deep neural networks. While these models often differ in the architecture of the underlying neural network [11–13,15], the general idea underpinning the different models is similar: through training, these models learn the complex *joint* probability distribution of sequence and structure (or sequence-structure-function), and can use this distribution to directly propose novel enzyme sequences with any desired characteristic. By learning these complex relationships, these models can, at least in theory, generate sequences that are more likely to fold correctly and possess the targeted activity without the need for extensive library construction and screening. Despite their promise, current end-to-end AI-based generative approaches often suffer from a critical limitation: the lack of interpretability. In other words, while these models can generate novel sequences exhibiting the desired properties, the inner workings of the generative mechanism and the underlying reasons for their success or failure, often remain opaque. This lack of interpretability hinders our ability to rationally refine the design process, troubleshoot unsuccessful designs, and extract generalisable principles for enzyme engineering. For example, two recently proposed algorithms for enzyme generation, ProGen [11] and Raygun [12], lack the explicit possibility to preserve specific residues in the generative process, making it impossible to ensure the catalytic site remains present. In this case, multiple mutants must be generated and only those containing the catalytic site are chosen for downstream experimental testing. In general, understanding *why* a specific mutant is generated is crucial not only for building trust in these methods, but also for guiding future design efforts beyond a trial-and-error paradigm, and to integrate previous knowledge into the design campaign (for example, which residues are part of the catalytic site and must be preserved). These problems are further exacerbated if the goal is to build even more complex algorithms to drive multi-objective optimisation, where not only the catalytic activity but also other properties, such as thermal stability, should be enhanced.

To alleviate some of the aforementioned issues, we present a generative algorithm based on the relation between a specific residue and its embedding vector representation encoded by a pLM, describing its local context within the amino acid sequence. In practice, we use this relation to define an energy landscape where, by construction, the minimum energy corresponds to the target enzyme whose function we wish to mimic, and higher energies correspond to mutants with varying degrees of change in the environment around the residues that make up the catalytic site. By sampling this energy landscape via standard MC, we show how simply increasing the effective temperature allows us to reliably generate mutants that preferentially sample a larger neighbourhood around this minimum, while still remaining structurally close to the original enzyme and sampling highly diverse sequences.

### 1.1 Using embedding similarity as a bias to generate mutants conserving the structure of specific regions

Decades of research on proteins and their evolution demonstrated that correlations between the chemical identities of amino acid residues can reveal how mutations propagate through a protein’s local environment [7,16,17]. This correlation can be direct, e.g., when two residues are spatially close to each other, but also indirect, e.g., when they share a neighbour. As a result, when a single amino acid residue is altered, it can influence a neighbouring residue. This change then cascades, affecting other amino acids further along the chain, even if they were not directly adjacent to the initial modification. As such, rather than depending on their linear distance along the protein backbone, correlations are expected to decay with the distance between residues in the folded structure. This latter idea has been exploited to build protein folding algorithms, including the most recent and accurate ones, such as AlphaFold [18], RosettaFold [19] or ESMFold [7]. Among these algorithms, ESMFold derives these correlations between residues from a pLM called ESM-2 [7], which was trained to solve a so-called masked language modelling problem. In this type of training, random residues are masked, and the algorithm is tasked with retrieving their identities given the surrounding sequence. Learning how to solve this problem is equivalent to learning correlations between residues: if the pLM can correctly identify that two residues in a sequence are strongly correlated, the presence of one of them can be used to increase the chance of correctly guessing the identity of the other, as opposed to a random guess. In technical, but quite illustrative jargon, these correlations are used to reduce the language model’s perplexity. Once trained, ESM-2 takes a sequence of amino acids as input and outputs contextual information in a multi-dimensional vector for each residue, its so-called embedding vector, $\vec{\epsilon }$. In a transformer architecture, embedding vectors are built as a combination of terms whose expansion coefficients are determined by the (self-) attention matrix **A**. The entries of such matrix $A_{ij}$ are interpreted as a measure of the strength of correlation between the (*i*,*j*) pair of residues, and, as a result, the embedding vector contains information regarding a residue within the context of the specific amino acid sequence provided as input. For this reason, these vectors can, and have been, successfully used as the starting input to a variety of algorithms for different downstream tasks. Crucially for our purposes, these tasks included structural ones, such as understanding the local fold to which a specific residue belongs, or the prediction of residue-residue contacts. In fact, per-residue embedding vectors given by ESM-2 are used by highly accurate ESMFold to reconstruct a protein’s three-dimensional structure from its sequence alone [7,20].

Following this interpretation, our main hypothesis is that if one changes the identity of one amino acid residue, the change in the embedding vector of the remaining ones is a measure of how much this mutation changed their local environment. Specifically, if a mutation in a residue *X* does *not* affect the local environment of a residue *Y*, then the embedding vector of residue *Y* should also be minimally affected, and vice versa. In this regard, we should also highlight that the magnitude of such a change should not only be affected by how strongly residues *X* and *Y* are correlated, but also by which amino acid *X* is substituted with. For example, substituting *X* with an amino acid that is biochemically similar might lead to a small change in *Y*, even when *X* and *Y* are strongly correlated. Importantly, pLMs encode knowledge of chemically conservative substitutions, allowing them to distinguish between structurally benign and disruptive mutations.

We formalise this previous hypothesis to build an energy function to quantify the effect of mutations. This energy function is then used within an algorithm that, starting from a reference protein, can generate mutants that retain the structure and local environment of any user-defined set of protected residues {*P*}. Due to the structure-property relation, when this protein is more specifically an enzyme and {*P*} is chosen to be the putative catalytic site, we would expect such enzyme to also preserve its function. In other words, by preventing mutations that will make the enzyme unfold, our algorithm can generate a pool of candidates depleted in what would be otherwise deleterious mutations, thereby reducing the number of experimental screenings necessary in directed evolution approaches for enzyme optimisation.

**Fig 1:**
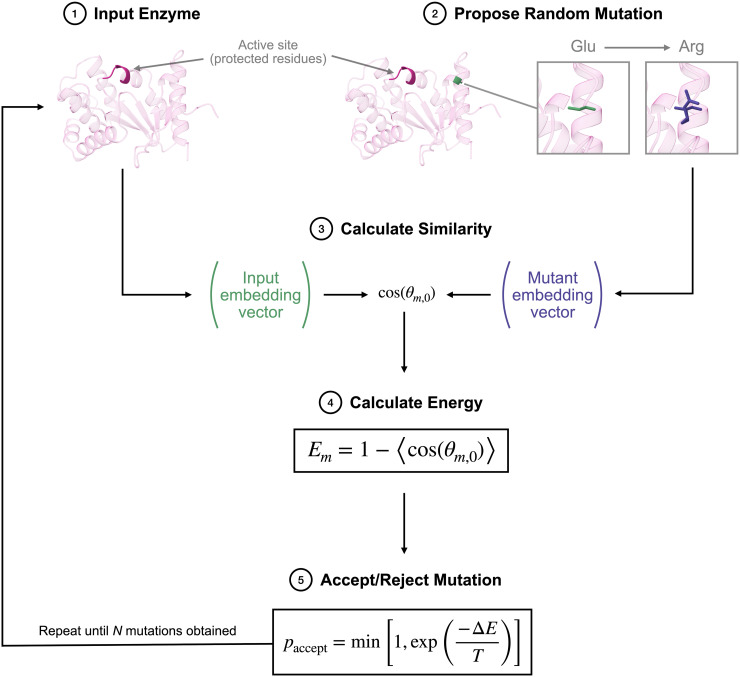
Flow chart summarising our generative algorithm. Starting from a reference enzyme as input, a random mutation is made. The impact of this mutation is quantified by computing the change in embedding vectors, derived from the ESM-2 pre-trained pLM, for a user-defined set of residues {*P*} whose local environment is to be preserved. This change, measured by cosine similarity, defines an effective energy used to guide a standard Metropolis MC sampling algorithm to accept or reject the mutation, following Eq. 1. The well-known mathematical properties of this type of algorithm guarantee that the equilibrium distribution of sequences generated spans all the possible mutants within a certain energy from the original one. Correspondingly, when the connection between a residue’s embedding vector and its environment holds, such a procedure generates mutant sequences that maintain the spatial configuration around {*P*}. If the choice of {*P*} carefully includes all residues in the catalytic site, this process effectively generates a library of enzyme variants with likely preserved catalytic activity.

**Fig 2:**
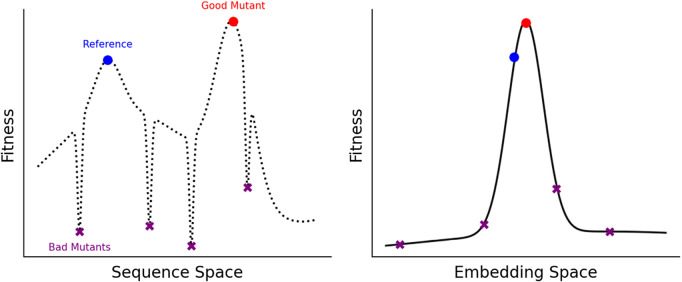
Fitness landscape schematic comparison between sequence and embedding spaces. In sequence space (left), structurally or functionally similar mutants, e.g., the reference enzyme and a viable mutant, are often separated by low-fitness intermediates, making naive exploration problematic and inefficient in directed evolution. In contrast, in embedding space (right), such mutants can cluster together, smoothing the landscape and removing fitness “gaps.” Sampling in the embedding space enables the generation of diverse, high-fitness candidates while avoiding deleterious variants, making it a more effective starting point for downstream experimental optimisation.

A graphical representation of our algorithm is provided in Fig 1. In simple words, our approach goes as follows: i) start with the original amino acid sequence of the enzyme to mimic; ii) propose a random mutation in one of the residues; iii) decide whether to accept or reject the proposed mutation based on how much the embedding vectors of the amino acids in the catalytic site have changed, using a Metropolis MC acceptance criterion; and iv) repeat points ii) and iii) to generate as many samples as requested. The general outline of this approach in itself is not novel. In the context of statistical sampling of a probability distribution, this algorithm has been used (e.g., for molecular simulations) at least since the 1950s [21], while also being recently employed in enzyme design [22]. However, to the best of our knowledge, we are the first to propose the underlying acceptance criterion based on an energy function built on the connection between an amino acid sequence, its residues’ embedding vectors as provided by a pLM, and these residues’ local environment. This connection is crucial because it provides an easy route towards generating mutants that, while arbitrarily different from the original enzyme in any other aspect, can conserve any specific part of the enzyme and its structure. Unlike one-shot generative approaches based on deep-neural networks, where such conservation cannot be guaranteed, this capability allows us to generate mutants that preserve the key ingredients necessary to preserve catalytic activity. By avoiding deleterious mutations, our algorithm enriches the library in variants that are more likely to have higher fitness. We should stress, however, that while necessary (see also Section 2.5), preservation of the local environment is still not sufficient to guarantee catalytic activity. Fig 2 illustrates this advantage and contrasts embedding-guided sampling with the purely sequence-based exploration typical of directed evolution. Additionally, the definition of a generalised energy to produce mutants via MC sampling allows us to provide a robust interpretation of the generative procedure in terms of statistical mechanics concepts.

### 1.2 An energy function built on residues’ embedding similarity and its use for sampling

Before we analyse the results of our generative approach, we briefly outline the underlying mathematical framework and highlight key properties of the sampling algorithm. Central to our method is an energy function defined in the latent space of the ESM-2 pLM, which encodes contextual embeddings for each residue in a protein sequence. We define the energy of a mutant, $E_{m}$, with respect to a set of *protected* residues {*P*}, as:


$$\begin{array}{c} E_{m}(\{P\})=1-\left\langle \cos(\theta_{m,0})\right\rangle =1-\frac{1}{N_{P}}\sum_{r\in \{P\}}{\vec{\epsilon }}_{m,r}\cdot {\vec{\epsilon }}_{0,r} \end{array}$$ (1)

Here, ${\vec{\epsilon }}_{m,r}$ is the embedding vector for residue *r* in mutant *m*, and ${\vec{\epsilon }}_{0,r}$ is the corresponding embedding in the reference sequence. The sum is taken over the set of protected residues {*P*}, with $N_{P}=|\{P\}|$ denoting the number of protected residues. These are residues whose local environment will be preserved. In the case of an enzyme, the protected residues are typically those directly involved in the catalytic site, or those located within a certain distance from it in the folded structure (see Table 1 for the specific residues used in this work). The energy function therefore quantifies the extent to which the embeddings, and by extension, the local environments, of the protected residues have changed in the mutant sequence. Importantly, $E_{m}$ is not the inverse of a fitness function (as in Fig 2) but is instead defined in an *effective* embedding-similarity space.

**Table 1:** Summary of the enzymes analysed in the main text along with their putative protected residues. For each enzyme, structural comparisons between three engineered mutants (S1, S2, S3) and the reference structure are quantified using the root-mean-square deviation (RMSD) of the protected residues. Sequence identity percentages for the full-length enzymes relative to the parent are also provided. Additional enzymes are detailed in the S1 Appendix, Table B.

PDB ID	Class	Protected Residues, {*P*}	Protected RMSD (Å)			Sequence Identity (%)		
			S1	S2	S3	S1	S2	S3
1A2J	Oxidoreductase	Cys-30, Cys-33	0.035	0.033	0.044	53	24	9
1EDG	Cellulase	Arg-79, His-122, Asn-169, Glu-170, His-254, Tyr-256, Glu-307	0.188	0.184	0.204	81	51	44
1UA7	Hydrolase	Asp-173, Glu-205, Asp-266	0.243	0.070	0.229	39	29	14
1TAQ	Taq polymerase	Asp-610, Ile-614, Glu-615, Phe-667, Tyr-671, Lys-663, Arg-659	0.130	0.234	0.280	95	71	27

Using this definition of the energy, sequence generation proceeds via a standard Metropolis MC sampling procedure [21,23,24]. At each step, a random mutation is proposed to any of the amino acids that are not part of the protected residues, and is accepted with probability:


$$\begin{array}{c} p_{m\to m+1}=\min\left[1,\exp\left(\frac{-\Delta E_{m,m+1}}{T}\right)\right] \end{array}$$ (2)

where $\Delta E_{m,m+1}=E_{m+1}-E_{m}$ is the difference in energy between the proposed mutant at step *m* + 1 and the current state of the system *m*.

From a mathematical point of view, the chain of states (i.e., amino acid sequences) generated by using a Metropolis MC algorithm and the acceptance criterion defined by Eq. 2 is a discrete-time Markov chain [25] sampling a Boltzmann probability distribution characterised by the energy function *E*. Under very mild assumptions, statistical mechanics thus implies that, in the long-time limit, our algorithm samples a region minimising the free-energy F(T)=E−TS(E), where $S(E)=k_{B}\ln W(E)$ is the entropy of the latent space at a specific energy *E*, and *W*(*E*) is the number of states (i.e., amino acid sequences) with such energy.

This formulation presents us with an important property. The effective temperature *T* controls how far in energy mutants will sample from the energy minimum, which by construction, corresponds exactly to the reference sequence. In other words, starting from our reference sequence, and after an initial transient, the equilibrium distribution sampled by our generative algorithm will correspond to sequences within a certain distance in latent space from the reference sequence. If a small distance in energy or latent space corresponds to sequences whose folded structure preserves the local environment of the protected residues, our algorithm guarantees to generate similarly folded sequences, regardless of other differences. For example, while a low temperature will limit the distance sampled in latent space from the original enzyme, there is nothing limiting sequence diversity besides the explicit conservation of the protected residues imposed by the mutation proposal. Thus, the number of amino acids that are different in the reference compared to the mutant can be, at least in principle, arbitrarily large to the upper limit of $N-N_{P}$, where *N* is the total sequence length. While correlations between sequence and structure will still implicitly limit how far in sequence the mutant can be from the reference enzyme, our algorithm is mathematically guaranteed to sample all sequences within a certain energy region without any bias, and thus generate the maximum diversity allowed within this space.

## 2 Results

**Fig 3:**
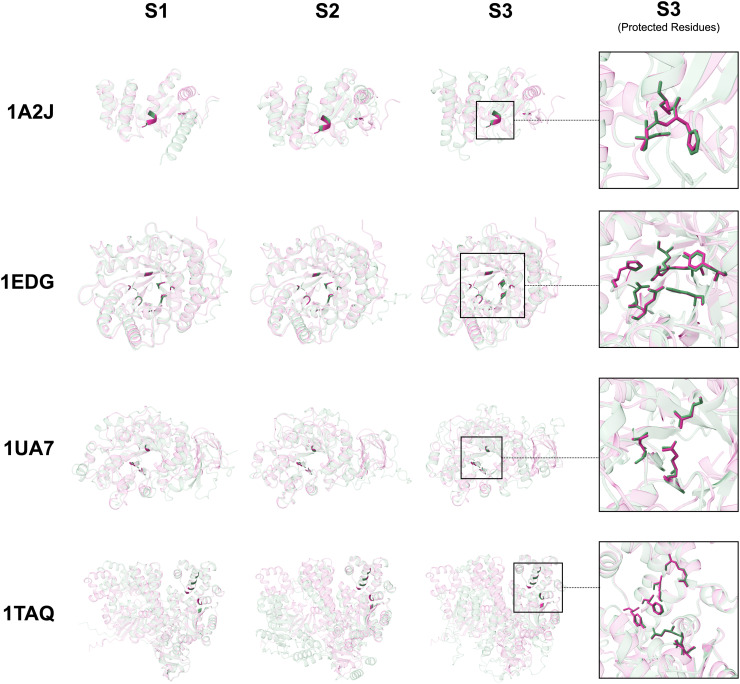
Examples of mutants for different representative enzymes generated using our methodology, highlighting its protected catalytic site. Each row corresponds to one of four representative enzymes (three mutants each), with the right-hand column showing a zoom of the catalytic site. The pink-shaded cartoon is a ribbon representation of the structure of the original enzyme, and the green-shaded ones are the mutant (both predicted by ESMFold [7]), overlaid on top of each other. Dark regions correspond to the protected residues in the catalytic site. While the RMSD between the catalytic site of the original enzyme and that of the mutant remains extremely small (less than 0.3Å), the difference in the rest of the structure can be almost arbitrarily large, demonstrating the ability of our algorithm to generate very diverse enzymes while preserving the geometry of specific, user-defined regions. Additional details for these mutants can be found in Table 1. The PDB codes for the reference enzymes are: 1A2J = oxidoreductase, 1EDG = cellulase, 1UA7 = hydrolase, 1TAQ = Taq polymerase.

In Fig 3, we show twelve illustrative examples of mutants from four representative enzymes, generated using our approach, superimposed onto folded structures for the original enzyme. The folded structures from which the RMSD was extracted have been calculated using ESMFold (see details in the Methods section). To reduce the possibility of having generated adversarial sequences that might mislead the protein folding algorithm, calculations using AlphaFold2 have also been performed. These results are provided in the S1 Appendix, Table A and Fig A and Fig B, and fully confirm all trends observed using ESMFold.

In Table 1, we also report the RMSD of the protected residues between the original enzyme and the mutants. Across all reported structures, the RMSD remains below 0.3Å, indicating exceptional preservation of local geometry. This structural conservation is particularly remarkable given the broad range of sequence identities between mutants and the reference enzymes, which, for the four enzymes in Table 1, span from as low as 9% to as high as 95%. This proves that our algorithm is capable of generating mutants that, despite an almost identical environment for the protected residues, can differ largely in sequence.

Although the protected residues exhibit minimal structural deviation, the remainder of the protein structure shows greater variability. As visualised in Fig 3 and S1 Appendix Fig C, RMSD values outside the protected site can vary substantially. In some cases, the ratio between the global RMSD and the RMSD of the protected residues (both calculated using the original enzyme as reference) approaches three orders of magnitude, ranging from approximately 1–400. This broad variation reflects a key design feature of our method: it does not impose constraints on structural regions beyond the specified protected residues, allowing for substantial backbone and side-chain flexibility elsewhere in the protein. We also notice that all predictions made by ESMFold have very high confidence. The average predicted Local Distance Difference Test (pLDDT) [26], a standard metric of folding algorithm confidence in local structure predictions around a certain residue [7,18], is very high for protected residues, with a minimum of 0.87 for the 12 mutants depicted in Fig 3. In contrast, pLDDT scores for non-protected regions show greater variability, reflecting the trends observed in RMSD. Together, these results confirm that our embedding-guided algorithm reliably preserves the structural integrity of catalytically relevant regions, even when extensive sequence-level diversity is introduced.

### 2.1 Embedding similarity is indeed a good proxy for similarity in the local structure

**Fig 4:**
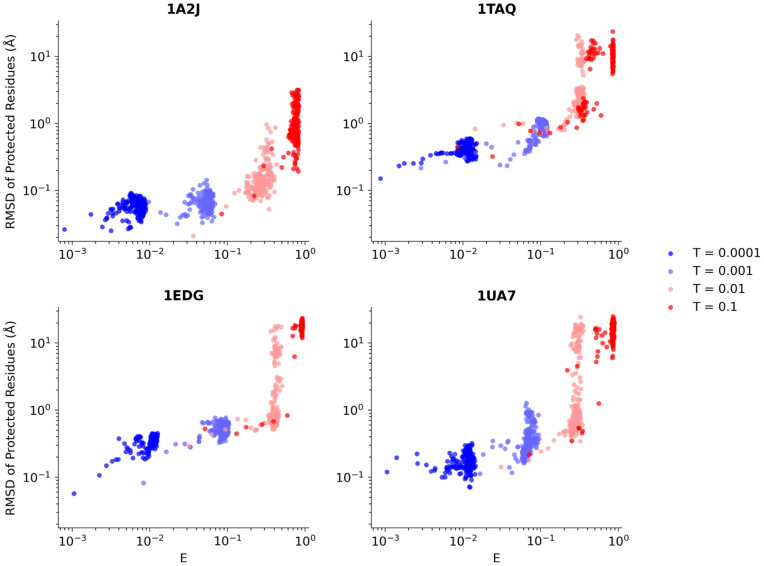
Scatter plots of the mutant energy, $E_{m}$, vs. the RMSD of the protected residues, for the four representative enzymes from Fig 3. We use the Spearman’s rank correlation coefficient, ρ, reported in Table 2, to measure the monotonicity of the function describing the connection between two variables plotted here. There is a strong correlation between the quantities, with lower energy values corresponding to lower RMSD values for the protected residues, allowing one to use the former as a proxy for the latter during the sampling procedure.

**Fig 5:**
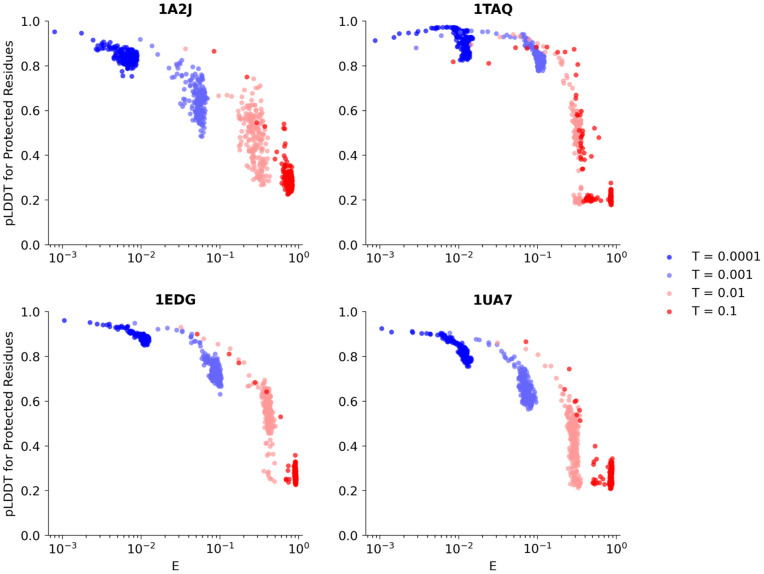
Relationship between pLDDT and mutation energy ($E_{m}$) for protected residues across four representative enzymes from Fig 3. The scatter plots reveal a consistent negative correlation: residues with lower mutation energies tend to exhibit higher pLDDT scores in ESMFold predictions. This trend is most prominent at low sampling temperatures, particularly at *T* = 0.0001, where all pLDDT values exceed 0.75, suggesting satisfactory structural confidence. The strength of these correlations, quantified via the Spearman’s rank correlation coefficient, ρ, is detailed in Table 2.

While the results above qualitatively demonstrated that our generative approach can produce sequence-divergent mutants with preserved local structure at user-defined residues, we now quantitatively assess the relationship between our embedding-based energy function (Eq. 1) and structural similarity, as measured by RMSD. This analysis is shown in Fig 4, which reports the RMSD of protected residues compared to the corresponding energies for a large number of mutants derived from the representative enzymes shown in Fig 3. A complementary analysis in Fig 5 reports a similar graph, but with the average pLDDT instead of the RMSD of the protected residues (additional data is provided in the S1 Appendix, Table C and D).

**Table 2:** Spearman’s rank correlation coefficient ρ between the mutant energy $E_{m}$ (see Eq. 1) and the RMSD of protected residues in the mutants generated in this study. The strong correlation, signalling a monotonic relation between energy and RMSD, supports the interpretation of the embedding vector as a descriptor for the local residue environment. Notably, a similarly strong correlation, but of opposite sign, connects our energy to the pLDDT, with the reported values also signalling very high confidence in the predicted structures for low enough energies. All reported correlations have a p-value <10^-4^.

Enzyme (PDB)	$\rho (E_{m},RMSD)$	$\rho (E_{m},pLDDT)$
1A2J	0.855	-0.877
1EDG	0.931	-0.944
1UA7	0.848	-0.852
1TAQ	0.924	-0.944

To quantify these trends, we calculate the Spearman’s rank correlation coefficient ρ [27], a statistical measure of a function’s monotonicity, whose modulus varies between 0 (no correlation) and 1 (perfectly monotonic function), with the sign indicating increasing (positive) or decreasing (negative) behaviour. As can be observed in Table 2, there is a consistently strong positive correlation between embedding-based energy and RMSD (ρ ranging from 0.848 to 0.931), as well as a strong negative correlation with pLDDT (ρ ranging from –0.852 to –0.944). These results confirm that lower-energy mutants, i.e., those more similar to the reference in embedding space, also tend to be more structurally similar and confidently predicted.

This is a critical finding, because while our MC procedure guarantees sampling over embedding-defined energy landscapes, this energy is only defined through the sequence embeddings of the protected residues, not structural deviations. Initially, we only hypothesised that these two quantities should have a strong correlation; here, we provide empirical evidence for this assumption (at least in a statistical sense). From a practical perspective, the observed correlation means that we do not need to fold structures at each step during sampling, which would increase computational costs by orders of magnitude. Instead we can simply take changes in the embeddings as a useful, computationally cheaper proxy to drive the generative procedure. In other words, to generate mutants with a conserved environment for the protected residues, we only need to sample low-energy regions in the embedding similarity space. This will implicitly correspond to sampling low-RMSD values with high confidence in the predictions made, as also shown by the strong (anti-)correlation with the pLDDT.

### 2.2 A reliable and robust generative procedure can be achieved by an appropriate choice of effective temperature

**Fig 6:**
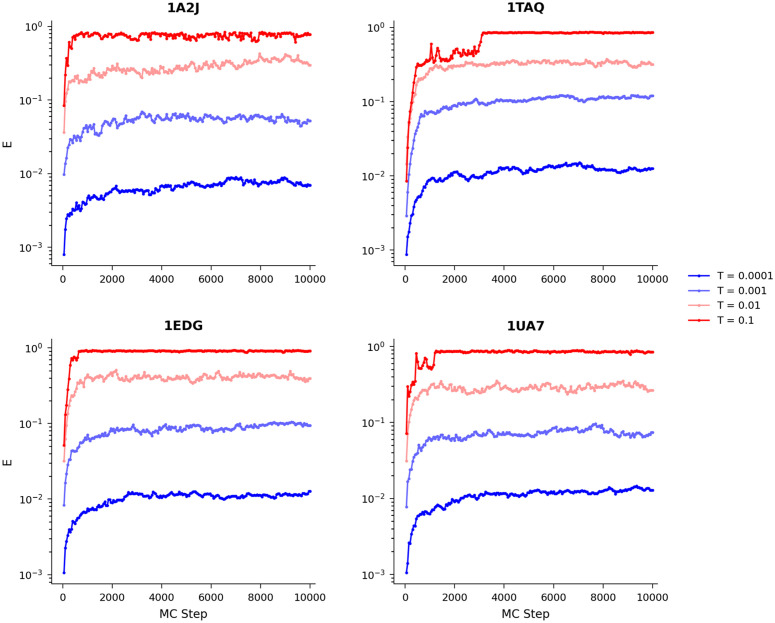
Trajectories of energy vs. number of MC steps during the generative procedure for the four representative enzymes from Fig 3. Low to high sampling temperatures are presented as a colour gradient from blue to red, respectively. After some initial transient, it is clear that the trajectory plateaus around an equilibrium energy value that is a monotonic function of the temperature, as expected from the properties of MC sampling.

When using MC sampling as a generative procedure, higher temperatures correspond to sampling regions of higher average energy. By contrast, lower temperatures constrain exploration to a narrow basin around the reference sequence. The mathematical convergence properties of the Markov chain associated with the MC trajectory guarantee that the generated mutants will be distributed around a well-defined energy region, once equilibrium is reached. This can be readily observed in Fig 6, where we show the energy as a function of the number of MC steps for different representative runs at different temperatures. As the number of MC steps (i.e., attempted mutations) increases, the energy plateaus around a value that increases as the sampling temperature increases. In all the cases analysed, if we choose a temperature for which the energy plateaus to a value below $E_{m}\approx 0.005$, our algorithm reliably generates mutants with very low RMSD of the protected residues, below 1Å, as well as with high prediction confidence, with most mutants showing an average pLDDT above 0.7.

**Fig 7:**
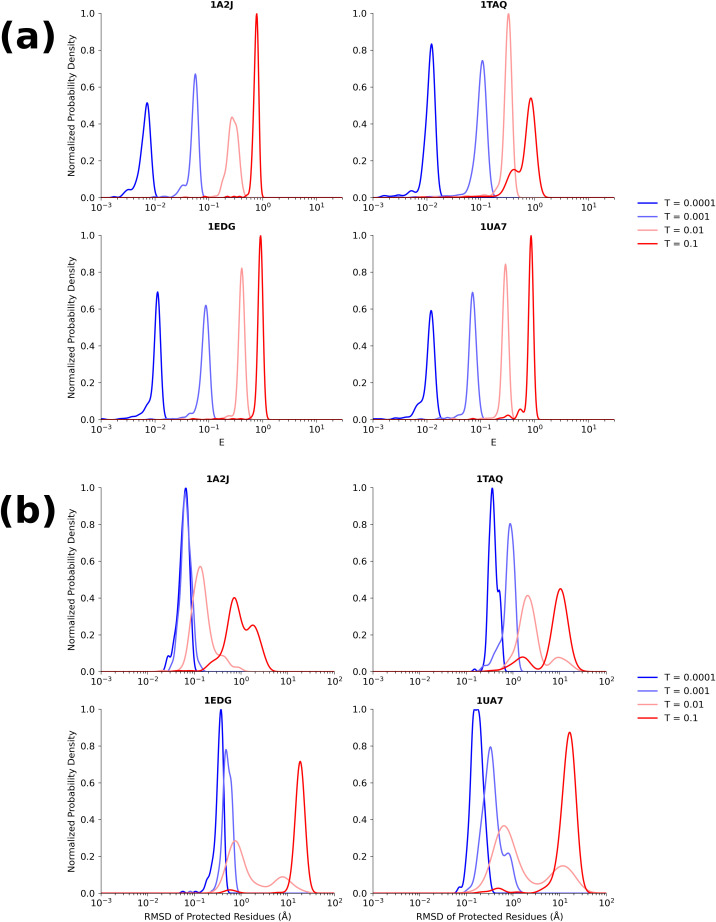
Temperature-dependent distributions of energy and RMSD across the four representative enzymes from Fig 3. **a)** Probability density function of the energy values observed (corresponding to the energy of different mutants) as a function of sampling temperature. **b)** Probability density function of the RMSD values as a function of sampling temperature. As a result of the generative procedure being based on a MC approach, sampling at increasing temperatures is equivalent to shifting the average value of the energy and RMSD sampled to higher values, providing an easy and physically justifiable way to control the generative procedure. The probability density function is estimated using Kernel Density Estimation and Scott’s rule [28] for the choice of the Gaussian kernel width. The peak of the highest distributions is normalised to 1.0, with the other distributions scaled proportionally, such as to keep the area under the curve the same across the distributions.

An alternative representation of the same data can be seen in Fig 7, where we report the probability density function of the mutant energies and protected residue RMSDs. Even in this case, it is clear that the distribution of mutants peaks both in energy and RMSD space, with the mode of the distribution moving to larger values for larger temperatures. Curiously, at high enough temperatures we also observe a phenomenon which we can relate to melting. For very low and very large temperatures, the algorithm produces distributions with a single minimum; however, around a critical temperature the system shows a bimodal distribution with peaks both at high and low RMSD, corresponding to an equilibrium between what we can interpret as an ordered (low RMSD) and liquid (high RMSD) phase. Anecdotally, but somewhat interestingly, our simulations also demonstrated that starting from a sequence generated at very high temperature, the system is usually unable to find the minimum even after reverting the temperature below the critical value, at least not given sampling of a few thousands of MC steps. A similar situation occurs when one quenches a fluid via molecular simulations, where the system can remain trapped in a disordered, long-lived amorphous state that is not representative of the equilibrium configuration of that temperature. In practical terms, the presence of this hysteresis means that for the generative procedure to be successful, one should always start and remain at a temperature below the critical melting temperature. While we empirically observe that a temperature of below 0.001 always satisfies this condition for all enzymes we have simulated, this temperature should be expected to be system-dependent. Furthermore, although we do not study this effect here in detail, we generally expect this temperature to also depend on the specific pLM used. Determining this critical temperature is relatively easy, based on the fact that above this threshold, very unfavourable mutations are accepted, resulting in random structures being rapidly generated which completely lose any resemblance to the reference enzyme. Based on previous observations, a simple protocol to find the maximum sampling temperature for any system involves running a series of simulations at logarithmically spaced temperatures and monitoring the equilibrium energy in each case. For a small subset of the samples generated, the corresponding RMSD of the protected residues can be measured. One can then simply proceed with the generative procedure at or below the highest temperature at which the RMSD is below an acceptable level.

### 2.3 The sampling temperature only weakly constrains sequence identity

While higher sampling temperatures lead to increased structural deviations from the reference enzyme, our energy function does not explicitly bias towards sequences close to the original one. Instead, the generative process samples sequences according to their embedding-defined energy, treating all sequences with equivalent energy equally, regardless of how similar they are to the reference. As a result, even mutants with low sequence similarity may be sampled at low temperatures, provided they preserve the local embedding environment of protected residues.

**Fig 8:**
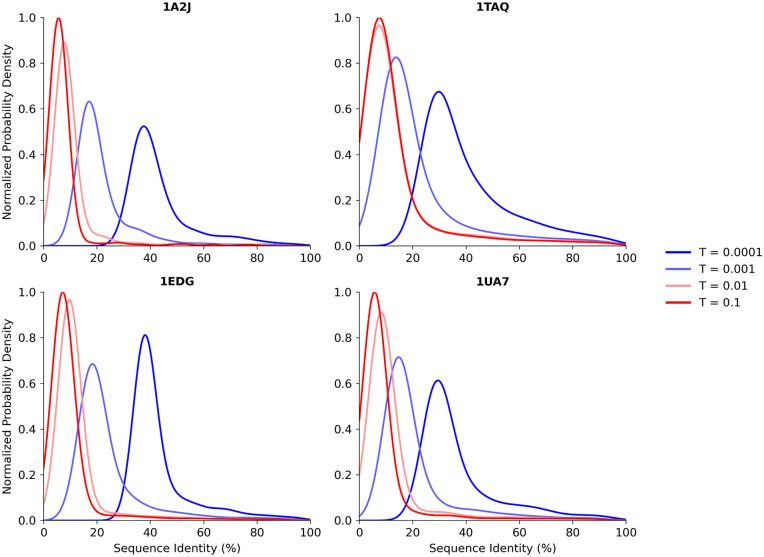
Probability density functions of the sequence identities obtained for the four representative enzymes from Fig 3 at different temperatures. Although in principle our algorithm does not explicitly bias sequence similarity, reducing the sampling temperature clearly results in structures of higher similarity. For the enzymes presented here, a sequence identity of 40% involves the mutations of approximately 110 amino acids for 1A2J and 500 amino acids for 1TAQ. Similarly to Fig 7, the probability density function is estimated using Kernel Density Estimation and Scott’s rule [28] for the choice of the Gaussian kernel width. The peak of the highest distributions is normalised to 1.0, with the other distributions scaled proportionally, such as to keep the area under the curve the same across the distributions.

This decoupling of structure and sequence is shown in Fig 8, where for the previously reported enzymes, we present the distribution of sequence identity for the generated mutants; here, defined as the fraction of non-mutated residues compared to the reference sequence. As expected, higher temperatures broaden the distribution, enabling more divergent sequences. However, even at the lowest temperature tested (*T* = 10^-4^), where average protected residue RMSDs remain well below 1Å, sequence identity can drop to as low as 20%, corresponding to hundreds of residue differences.

These results highlight how our algorithm can simultaneously maintain local structural integrity and achieve substantial sequence diversity; a property that is essential for effective exploration of sequence space in enzyme engineering, as it enables the generation of highly novel variants while avoiding mutations that would compromise functionally critical regions. Importantly, the ability to generate diverse sequences without compromising structural constraints avoids a common failure mode of AI-driven generative models: mode collapse, where a model fails to produce diverse samples, and instead, yields a few high-probability outputs.

Taken together with the strong correlation between embedding energy and RMSD, these findings confirm that sampling low-energy regions is sufficient to ensure structural conservation at protected sites, while still supporting broad sequence exploration. This balance between constraint and diversity positions our method as a powerful tool for designing enzyme variants with high functional potential.

We also highlight here that, on this structural-preservation axis, our method compares favourably with standard approaches for *in silico* directed evolution based on point or pairwise substitution correlations derived from Multiple Sequence Alignment (MSA), namely, sampling guided by BLOSUM-derived substitution energies or by Potts models fitted via mean-field Direct Coupling Analysis (DCA) (see the S1 Appendix, Fig F and Fig G). Specifically, sampling based on our pLM-derived embedding energy (Eq. 1) achieves higher sequence diversity at matched protected-site RMSD and, conversely, lower structural distortion for a given level of sequence diversity. Quantitatively, pLM-guided sampling reaches sequence identities as low as ∼20–30% while keeping the protected-site RMSD at or below ∼0.3 Å, whereas DCA- and BLOSUM-guided sampling remain confined to ∼70–90% sequence identity at comparable RMSDs and, when pushed to matched levels of sequence diversity, incur distortions exceeding ∼1 Å for DCA and reaching several Å for BLOSUM (Fig G and Fig F). We stress that this comparison is purely structural: it does not constitute an experimental fitness benchmark, and the relative merits of pLM- and DCA-based generative approaches in terms of functional yield remain to be assessed directly in the wet lab.

### 2.4 Low embedding energy variants further preserve the local dynamics of the wild type

**Fig 9:**
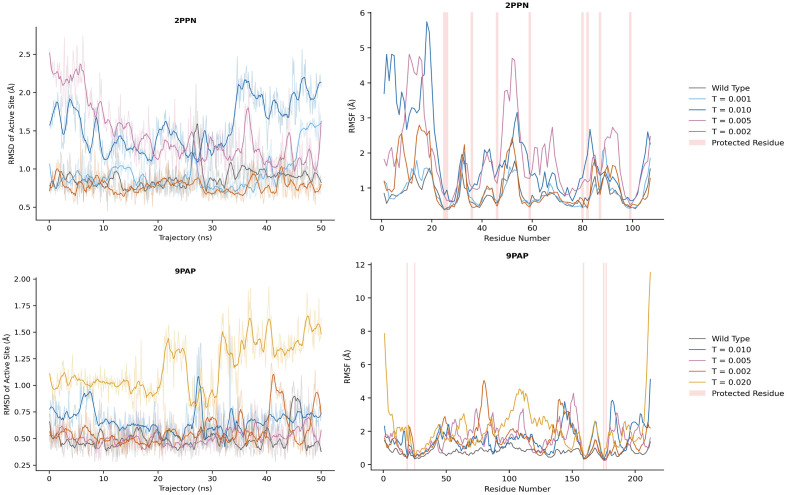
Molecular dynamics simulations of representative mutants generated at different sampling temperatures. Left column: time evolution of the RMSD (see Eq. 3) of each mutant with respect to the mean positions of the original enzyme. For mutants generated at low sampling temperature, the active-site geometry remains very close to that of the original enzyme throughout the entire trajectory, even when thermal fluctuations are included. Right column: RMSF averaged over time (see Eq. 4), for each residue; shaded regions indicate protected residues (i.e., those belonging to the putative catalytic site and thus preserved during the generative procedure). While large fluctuations can and do occur elsewhere – possibly indicating unfolding of specific regions – residues in the active site remain extremely stable throughout the trajectory, with fluctuations below 1Å, consistent with a well-defined folded state, as is also apparent from visual inspection of the individual trajectories. Sequence identities for these mutants range from ≈6% (9PAP, *T* = 0.01) to 37% (2PPN, *T* = 0.001). Additional simulation data and trajectory analysis are shown in S1 Appendix, Fig H, Fig I and Fig J.

The folding algorithms used to assess deviations from the wild-type active-site geometry provide only a static view of the enzyme. A further well-known limitation is that deep-learning-based folding algorithms, having been trained predominantly on structures with ordered domains, tend to over-stabilise the predicted folds [29]. To complement this static picture with an orthogonal computational perspective, and to probe the dynamics of the active site and how these differ between the wild type and our generated mutants, we performed Molecular Dynamics (MD) simulations (details in the Methods section). Results are summarised in Fig 9 for two representative enzymes, corresponding to wild types with PDB IDs 2PPN and 9PAP (drawn from the broader supplementary library (see Data Availability Statement, and S1 Appendix Table B), as illustrative examples for the MD analysis). Qualitatively consistent results for all 13 enzymes studied are reported in S1 Appendix, Fig H, Fig I and Fig J. The left column shows the time evolution of the RMSD along the MD trajectories, defined as:


$$\begin{array}{c} \text{RMSD}(t)={\left\langle {\left(x_{i}^{M}(t)-{\left\langle x_{i}^{WT}(t)\right\rangle }_{t}\right)}^{2}\right\rangle }_{i}, \end{array}$$ (3)

where *t* denotes time, *i* indexes residues within the protected site {*P*}, and superscripts *M* and WT denote the mutant and wild-type positions, respectively. This RMSD, computed after alignment via the Kabsch algorithm, uses the time-averaged active-site position of the wild type as reference. Comparison with the wild-type RMSD shows that, at least for enzymes sampled from the equilibrium distribution at low sampling temperature (and thus exhibiting low embedding energy), the active-site geometry is preserved not only on average, as predicted by the folding algorithms, but also when thermal fluctuations are taken into account (note that the MD temperature and the sampling temperature are unrelated quantities).

The right column reports the mean per-residue fluctuations over the same trajectories, now defined as:


$$\begin{array}{c} {\text{RMSF}}_{i}={\left\langle {\left(x_{i}^{M}(t)-{\left\langle x_{i}^{M}(t)\right\rangle }_{t}\right)}^{2}\right\rangle }_{t}. \end{array}$$ (4)

For residues within the protected site, these fluctuations remain small for both the wild type and the low-temperature mutant, as is also readily apparent from visual inspection of the trajectories: the active site remains folded over the simulated timescales, with fluctuation magnitudes in the mutant practically identical to those of the wild type. Taken together, these results show that low-temperature mutants preserve not only the average active-site geometry but also its dynamics and flexibility.

These observations do not constitute direct confirmation of catalytic activity, which would require experimental validation. However, previous work [22] has shown that short (10 ns) trajectories are effective at identifying poor variants that rapidly adopt non-productive conformations. The preservation of both local geometry and active-site flexibility *in silico*, therefore, suggests that our algorithm could enrich for functional sequences, making the generated mutants promising candidates for downstream experimental testing.

### 2.5 Comparison to experimental data

While a comprehensive experimental validation of all generated mutants will require a community effort (and for this reason we provide all sequences and data as open-source resources), we can use existing experimental data to address a crucial question: *does a low embedding energy, which we have linked to preservation of the active-site geometry, also translate into a functional enzyme?*

To investigate this question, we use the experimental data from Russ et al. [30], who studied chorismate mutase, an enzyme whose catalytic region contains highly conserved residues and which is represented by a large number of natural variants across species. In their study, approximately 1130 natural variants were synthesised and assayed for catalytic activity, alongside approximately 1,600 synthetic sequences generated using Boltzmann Machine Direct Coupling Analysis (bmDCA) [31], a machine-learning approach that learns statistical patterns and correlations from an MSA of 1,259 AroQ-family chorismate mutase homologs. The synthetic sequences span a wide range of sequence identities, from approximately 90% down to approximately 20%, relative to their closest functional natural variant in the MSA. For each synthetic variant, we compute the embedding energy as defined by Eq. 1, using as reference the closest functional natural variant, i.e., the same protocol one would follow in practice when starting from a natural enzyme and aiming to preserve its function (for this same reason, we analyse here only the 999 sequences that preserve the catalytic site). As an additional baseline, we also generate 500 random mutants by introducing point mutations into randomly selected functional natural sequences, spanning sequence identities from 20% to approximately 100% while preserving the catalytic residues. Especially at low sequence identity, these random variants are expected to have vanishingly low probability of being functional [30].

**Fig 10:**
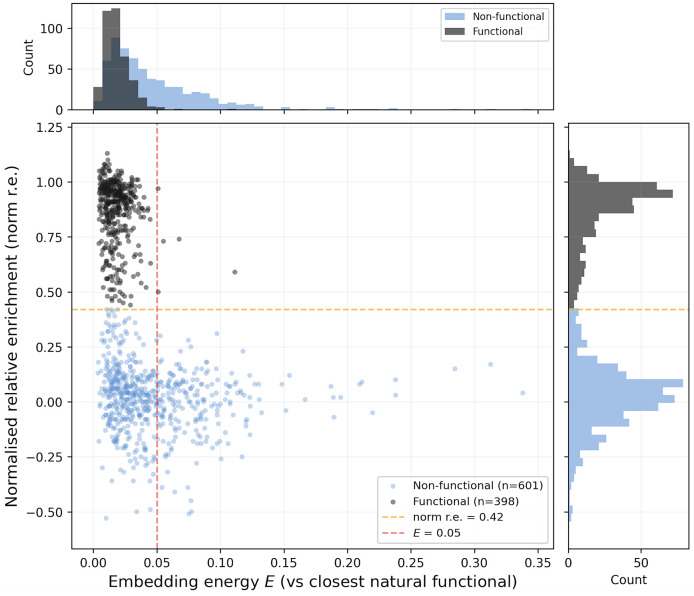
Embedding energy vs catalytic function for synthetic variants from Russ et al. [30]. The embedding energy (see Eq. 1) was calculated for synthetic mutants previously characterised in [30]. Normalised relative enrichment (NRE) was also measured by the same authors after expression of the mutant in *E. coli* and used to gauge an enzyme’s catalytic activity, with enzymes showing NRE > 0.42 being classified as functional. Nearly all functional synthetic variants (98.7%) show that our pLM-derived embedding energy is lower than 0.05, supporting the conclusion that while low embedding energy is not sufficient, it is a necessary condition for functionality.

The results are summarised in Fig 10. Our findings mirror the pattern reported by Russ et al. [30] using their statistical energy from the bmDCA model, here replaced by our pLM-based embedding energy. Specifically, we observe that while a low embedding energy does not guarantee functionality – consistent with the expectation that preserving the active-site geometry is necessary but not sufficient for catalysis – a high embedding energy is a strong predictor of non-functionality: for $E_{m}\geq 0.05$, only 2.3% of synthetic variants are functional, and for $E_{m}\geq 0.15$, none are. Importantly, even among synthetic sequences with less than 50% sequence identity to any natural variant, those classified as functional consistently exhibit low embedding energy, suggesting that the metric captures genuine structural preservation beyond mere sequence similarity.

It is worth noting that in Russ et al. [30], catalytic activity, and therefore the classification of variants as functional or non-functional, was measured upon expression in *E. coli*. However, the MSA from which their model was trained includes chorismate mutases from diverse species, which may function optimally under different conditions or expression systems. Some synthetic variants classified as non-functional might therefore retain activity in other contexts, introducing a potential bias in the experimental labels.

To further support our conclusions, we compare the synthetic variants against the randomly generated baseline (see S1 Appendix Fig K). The cumulative energy distributions reveal a clear separation: while 98.7% of functional synthetic variants have $E_{m}<0.05$, only 41.8% of random mutants fall below this threshold, and, crucially, all random mutants with $E_{m}<0.05$ have much higher sequence identity to their closest natural functional variant, indicating that their low energy simply reflects high similarity to the reference. By contrast, synthetic variants that achieve low embedding energy do so across a much broader range of sequence identities, demonstrating that the bmDCA generative model, unlike random mutagenesis, preserves the catalytic-site geometry even at substantial sequence divergence.

Taken together, these results demonstrate that the embedding energy, as defined here and used for sampling, takes consistently low values for experimentally confirmed functional variants and is elevated for non-functional ones, serving as an effective filter for excluding variants that are very likely non-functional. This supports our hypothesis that Monte Carlo sampling guided by the pLM-based embedding energy can increase the probability of generating functional variants by removing those that are almost certainly not. It also supports the interpretation that similarity in the local environment, as measured by the embedding energy, correlates with preservation of functionality, providing a physically interpretable metric that is complementary to approaches based on the log-likelihood of the overall sequence, such as the statistical energy of DCA models.

## 3 Discussion

The method presented here introduces a general and interpretable framework for enzyme sequence generation that directly addresses several limitations of existing approaches. Below, we discuss the key advantages of our method relative to prior generative models, its current limitations, and the broader implications for protein design.

Our approach distinguishes itself from existing models in two fundamental ways. First, it enables explicit preservation of user-defined structural motifs, such as catalytic sites. This ability is similar to that of complex multi-step pipelines, including those with backbone diffusion, sequence in-painting, and refolding [32,33]; but unlike these end-to-end deep-learning models, our method guarantees preservation of key residues by design. This means we can guarantee that any engineered mutant will retain the essential catalytic site, a feature typically absent in simpler, one-shot generative approaches [11,12]. Second, a significant advantage of our model is that it does not require any additional training beyond the initial pre-trained pLM – a stark contrast to all other current models. This latter aspect offers two additional advantages.

First, the computational cost of our generative procedure is minimal, determined solely by the pLM’s sequence-to-embedding transformation, and is easily handled by low-end computational hardware, including desktop machines. The efficiency of such an algorithm is best measured by the number of independent variants that can be generated for a given computational budget, and can in principle be optimised in a system-dependent fashion through careful choice of parameters and mutation protocol (e.g., allowing more than a single mutation per MC step). Without any such optimisation, for the system sizes considered here – up to approximately 900 amino acids in length – one can already generate over 100 independent variants in under one hour on a standard laptop GPU. This baseline performance can be further improved through more complex sampling schemes (e.g., parallel tempering), or by parallelising the mutation process and leveraging batch predictions with a Heat Bath acceptance method instead of Metropolis MC [24].

The second advantage is our model’s generality. This stems from its pure transfer learning approach, which only requires the pLM to generate expressive, context-dependent embeddings that capture information about each residue’s environment. Because we solely extract information from the pLM (RMSD calculations are only for validation of our hypothesis, not generation), we expect our model to provide optimal mutants even for enzymes and proteins with unknown structures. This makes the approach particularly attractive for working with large databases of uncharacterised sequences lacking structural annotations. In principle, by not relying on any additional fine-tuning, our model is compatible with any modern pLM that provides context-aware embeddings. While we use ESM-2 due to its proven structural relevance (e.g., as used in ESMFold), alternative models such as aminoBERT [8] or the more recent AMPLIFY [10] could also be employed.

Beyond practical advantages, our method rests on a well-understood theoretical foundation. By employing a decades-old MC sampling method, we inherit its asymptotic convergence properties: in the long-time limit, the Markov chain samples a Boltzmann distribution associated with our energy function, favouring low-energy (structurally conserved) mutants with exponentially higher probability than high-energy ones. We stress that these are asymptotic guarantees: as for any Markov chain in a high-dimensional sequence space, the mixing time is not knowable *a priori*, and finite-length runs may under-sample distant regions. In practice, we monitor energy trajectories to diagnose stationarity (Fig 6) and find that, for the systems considered here, mutants with low sequence identity to the wild type are reached on accessible timescales, though we make no claim of exhaustive coverage of the relevant sequence space. With this caveat, the MC formulation still offers concrete benefits over end-to-end deep neural network-based generative approaches: it provides a principled link between low-energy sampling and structural conservation, and reduces – though does not eliminate – the risk of mode collapse that can affect one-shot generative models. Additionally, and perhaps most powerfully, our procedure frames the generative process as sampling of an energy landscape. This unique formulation allows for systematic and flexible biasing of the search towards sequences with user-defined properties. Any feature predictable from a sequence, for instance, using a deep neural network, can be incorporated into the energy function as an auxiliary term. This lets us penalise undesired deviations and optimise toward specific target values for features like an enzyme’s ease of expression, or its thermal stability. Crucially, the MC framework imposes no differentiability or continuity constraints on these predictive models, enabling seamless integration of both regression and classification outputs. Unlike one-shot generative approaches where tuning the relative influence of multiple objectives is often intractable, our method allows for explicit and interpretable weighting of each energy component. This inherent modularity and tunability makes our approach exceptionally well-suited for multi-objective enzyme design, where diverse and often competing constraints must be balanced with precision.

Despite these advantages, our approach has several limitations worth acknowledging. First, the energy function (Eq. 1) sums exclusively over protected residues, providing no direct penalty for structural disruption elsewhere in the protein; in principle, a mutant could preserve catalytic-site embeddings while undergoing significant rearrangements in the rest of the scaffold, and fully addressing this would require systematic re-folding of each generated candidate as an additional filter. Second, since the generative energy and the primary structural validation metric both derive from ESM-2 representations, their correlation is not entirely independent; however, as shown in S1 Appendix, Table A and Fig A and Fig B, AlphaFold2 – which uses a fundamentally different model architecture, training procedure and includes MSAs as input – confirms the low-RMSD character of low-energy mutants, suggesting this circularity does not substantially bias our conclusions. Third, the sampling temperature *T* is currently determined empirically through a brief calibration sweep; given the low cost of ESM-2 embedding and ESMFold structure prediction, this sweep is computationally inexpensive, yet a principled criterion for its *a priori* selection remains an open problem. Finally, and most critically, we do not provide direct experimental validation of our generated sequences beyond the retrospective comparison to Russ et al. [30]: demonstrating catalytic activity experimentally for variants generated by our algorithm represents an essential and exciting next step, which we aim to facilitate by making all code and generated libraries publicly available.

Before we conclude, we highlight that the work and the results we present in this manuscript are better understood in the context of a wealth of recent literature on epistatic signals [34–40], that is, the context dependence of protein mutations, and its use to understand proteins evolution and protein design. In particular, various works have shown how methods such as DCA can detect epistatic signals, generating a roadmap of allowed vs forbidden mutations. Such roadmap, which naturally emerges following proteins evolution [39], can be leveraged to generate functional protein mutants. For example, Alvarez et al. [37,38] has shown how DCA can be used to generate new-to-nature protein sequences, and proved that the sequences designed using epistatic models are functional *in vivo*, while often exploring areas of sequence space that nature has not yet reached. Di Bari et al. [35] have previously discussed that these signals can be used to understand the timescales of evolution. What Alvarez et al. implied – and what our work confirms – is that the same signals can be used to speed up evolution *in silico*. In a similar vein, recent extensions of the DCA framework have been shown, with experimental validation, to produce more diverse functional variants than the standard formulation we benchmark against in the S1 Appendix Fig G [41,42]; a head-to-head experimental fitness comparison of our pLM-based sampler against these next-generation DCA variants is a natural – but out-of-scope – extension of the structural comparison provided here.

We also want to highlight here that we are not the first to show the power of deep learning and the transformer architecture in capturing epistatic signals. In fact, close to the spirit of our work, Sgarbossa et al. [36] previously used MSA Transformer, a pLM trained on MSAs, to go beyond the pair correlations learnt by DCA. This work also showed how such signals can be used to generate high-quality, diverse sequences that mimic the natural one. Similarly to our own observations, as previously described, the MSA Transformer generated sequences that are more diverse compared to those generated via a DCA model, exploring new, viable areas of the fitness landscape. Our work extends these results in one important way. While more generally the fact that a pLM purely trained on sequences alone, without the need of an MSA, can also capture co-evolutionary signals was extensively discussed in the original paper presenting ESM-2, we show here that these signals not only correlate more generally to sequence fitness and functionality, but that the learnt embeddings can be used as a direct measure of structural similarity in the local geometry of a given amino acid, extending their potential use to other tasks. For example, here we use them to generate new sequences that preserve the geometric structure of specific regions, but other applications could be imagined, not relying on structure modelling at all, but per-residue functional annotation alone. In fact, this also suggests that if epistatic signals can be captured by ESM-2, and similarly the embeddings strongly correlate to local geometry, then the reason why epistatic signals can be used to screen out non-functional variants is exactly because they provide an idea of what mutations can be made without affecting functionality.

## 4 Conclusions

In conclusion, we integrate a pLM with a statistical mechanics-based approach to introduce a generative algorithm that is able to create an optimal pool of mutants for experimental screening, starting from any initial enzyme. By construction, these mutants can be almost arbitrarily different in their sequence from the original one, yet preserve the catalytic site and its local structure, and are thereby depleted of deleterious mutations that would likely result in a loss of catalytic activity.

A core strength of the method lies in its theoretical foundation. By basing our generative procedure on solid statistical mechanics concepts and using a well-established algorithm such as MC sampling, we provide a clear and robust interpretation of our generative procedure, its convergence properties, and the role of the effective temperature as an intuitive control parameter. In addition to increasing our ability to understand and control the results, this hybrid method can be used to bias the generative procedure toward enzyme sequences that display any feature for which a sequence-feature function exists, simply by adding this feature as an additional term in the energy function. In the future, this aspect will allow us to go beyond the simple preservation of catalytic site shown here, and directly optimise for other application-relevant quantities, e.g., thermal stability, solubility or toxicity.

Finally, while our exposition focused on a limited set of four enzymes, we emphasise that the same qualitative trends and conclusions hold across a broader benchmark of 13 structurally and functionally diverse enzymes, spanning lengths from approximately 100–800 residues with extremely low sequence similarity. In total, our algorithm generated over 12,500 mutants across all 13 enzymes with catalytic site RMSD smaller than 2Å. Information about accessing this library, stored online on Zenodo, is provided in the Data Availability Statement.

Beyond static structural metrics, Molecular Dynamics simulations further confirm that low-energy mutants preserve not only the geometry but also the conformational dynamics of the wild-type active site, providing an orthogonal, physics-based validation of the algorithm’s output. We further establish a direct connection to experiment by comparison to characterised chorismate mutase variants [30], demonstrating that low embedding energy is a necessary condition for catalytic function.

Overall, our results represent a step forward in developing interpretable and controllable generative algorithms for enzyme engineering and, more broadly, protein design. By coupling the power of pLMs with the transparency of classical simulation techniques, our framework offers an alternative to current one-shot generative approaches. This hybrid strategy enables targeted exploration of sequence space with explicit control over structural constraints – crucial for optimising enzyme function through directed evolution. We suggest that the prevailing emphasis on end-to-end sequence generation may be misplaced for many practical applications, where interpretability, modularity, and precise constraint enforcement are more valuable than automated novelty. In this context, our approach offers a compelling path toward more rational and efficient protein engineering.

## 5 Methods

### 5.1 Embedding calculations

Residue embeddings for the Monte Carlo evolution algorithm were generated using the 650M-parameter ESM-2 model [7] (specifically, esm2_t33_650M_UR50D). Per enzyme sequence, one embedding calculation, performed on NVIDIA T4 GPUs via AWS, required less than 0.5 seconds for all the sequence lengths considered in this paper. The protected residues *P* for each enzyme were determined from literature search.

### 5.2 Folding via ESMFold and AlphaFold2

To validate our results and ensure robustness, we employed two state-of-the-art protein structure prediction tools: ESMFold and the ColabFold implementation of AlphaFold2 [43], to model the structures of the generated sequences and compare them to their respective reference enzymes. This dual-model approach helps guard against adversarial artifacts specific to a given predictor and strengthens confidence in the structural plausibility of the designed variants.

For ESMFold, calculations were run using Nvidia T4 GPUs using AWS, and took about 30 seconds per structure. Regarding ColabFold, the latter was used with the following parameters: template usage was disabled (use_templates = false) to prevent bias from known structures. MSAs were generated using MMseqs2 [44] against the UniRef sequence databases [45] (msa_mode = mmseqs2_uniref_env). The model type utilised was alphafold2_ptm, which incorporates predicted TM-score and alignment error metrics. Five distinct models were generated per sequence (num_models = 5), each undergoing three recycling iterations (num_recycles = 3) to refine predictions. Amber relaxation was not applied (num_relax = 0) to expedite computation. All ColabFold predictions were executed on NVIDIA A100 GPUs via Google Colab, with an average runtime of approximately 1500 seconds per structure. For our calculations, we used the structure with the highest overall confidence, as measured by the pLDDT. The RMSD between the predicted structures of the original enzymes and their existing experimental structures (codes 1A2J, 1EDG, 1TAQ, 1UA7 in the PDB database) is reported in Table A, along with confidence metrics of the predictions.

### 5.3 RMSD and RMSF calculations

RMSD between protein structures is defined up to a global translation and rotation. In all cases, we apply the optimal superposition that minimises the RMSD, computed using the Kabsch algorithm [46]. For the analyses presented in the main text, the alignment is restricted to the protected region of the enzyme; that is, the subset of residues whose geometry we aim to conserve, rather than the entire structure. To ensure a rigorous comparison, RMSD for the protected residues is calculated using all heavy atoms, including side chains. For the remaining residues, only backbone atoms (C, C${\;}_{\alpha }$, O, and N) are considered, as these atoms are universally present across all amino acid types and thus allow for consistent structural comparison. When calculating the RMSD between two structures over a dynamical trajectory, the deviation is calculated with respect to the time-averaged position of the wild-type enzyme, see Eq. 3. RMSF (see Eq. 4) is defined and calculated similarly to the RMSD, with the only difference that the average structure over time of the wild type is replaced with that of the mutant itself. In practice, RMSF measures how strong the fluctuations are within the same enzyme, rather than being a measure of the similarity between two different ones.

### 5.4 Molecular dynamics simulations

MD simulations were performed to assess the conformational stability and dynamics of enzyme structures generated by our algorithm. For each enzyme, the wild-type crystal structure was obtained from the PDB, and multiple mutant structures were generated by folding sequences produced by our MC algorithm at different sampling temperatures using ESMFold. All structures – wild type and mutant – were then subjected to the same MD protocol under identical conditions. Prior to simulation setup, crystal waters, hydrogens, and C-terminal oxygens were removed from each PDB-derived structure. All structures were then protonated and solvated using PDBFixer from OpenMM [47]. Simulations were carried out with OpenMM using the AMBER ff19SB force field [48] and the OPC water model [49]. Each system was placed in a periodic box sized to leave approximately 1 nm between periodic images of the protein. Sodium and chloride ions were added to neutralise the system and to achieve an overall salt concentration of 150 mM, representative of physiological ionic strength. Long-range electrostatics were treated with the particle mesh Ewald (PME) method [24], using a real-space cutoff of 1.0 nm and an Ewald tolerance of 10^-4^. Constraints were applied to all bonds involving hydrogen, and water molecules were kept rigid, with a constraint tolerance of 10^-4^.

Systems were first energy-minimised, then equilibrated in the NPT ensemble at 300 K and 1 bar for 50 ns using a 2 fs integration timestep, with trajectory and log output written every 100 steps. Production runs were subsequently performed under the same NPT conditions for 50 ns, with coordinates saved every 100 steps (0.2 ps). Trajectories were written in DCD format with solvent excluded.

Analysis was carried out using MDAnalysis. Periodic boundary conditions were handled by unwrapping coordinates with the built-in unwrap procedure using inferred bonds. For each enzyme, an additional production run was performed starting from the cleaned wild-type structure alone, and the resulting trajectory was used to compute a backbone-aligned time-averaged structure. These wild-type average structures served as a common reference for comparing variants generated at different sampling temperatures (e.g., for structural alignment and visual comparison).

## Supporting information

S1 AppendixTable A. Structural prediction accuracy between ESMFold and AlphaFold2 for the reference sequence of the enzymes discussed in the main text.RMSD values are reported in Å, and pLDDT scores are presented in the range of [0,1], with 1 representing the highest confidence. RMSD and pLDDT assess the fidelity of predicted structures relative to experimentally determined conformations and the model confidence in the prediction, respectively. In 1TAQ, the high RMSD stems from the wrong predicted alignment of two domains, each of which, however, is internally described with very high accuracy. Fig A. **Correlation between ESMFold [7] and AlphaFold2 [43] RMSD prediction for a random subset of mutants generated during our simulations**. The subset was specifically chosen to sample a wide range of energies and RMSD of the active site. As somewhat expected, predictions are more highly correlated in the small RMSD region (≤ 1.5 Å), as shown in the inset on the right, which also generally corresponds to sequences of higher similarity with the original ones (as discussed in the main text). High-energy regions often correspond to almost random sequences, for which we do not expect a protein folding algorithm to work particularly well because of the lack of a reference system close enough for the inference process to provide a reliable result, as also shown by the drop in the confidence metric, see Fig B. However, such high-RMSD sequences are also irrelevant from a practical point of view, considering keeping the active site intact is required to preserve catalytic activity. Fig B. **Correlation between ESMFold and AlphaFold2 pLDDT confidence scores for a random subset of mutants generated during our simulations**. There is a strong correlation between the pLDDT from both models, which is evident from the clustering of points along the diagonal. Predictions for lower energy mutants tend to have a higher confidence. The same subset from Fig A was used for this analysis. Fig C. **Comparison of RMSD for protected residues vs. non-protected residues for all four representative enzymes from Fig 3, as a function of sampling temperature.** In general, lower temperatures correspond to lower RMSD values for the entire enzyme. However, the RMSD of the protected residues is always lower due to the residue preservation nature of our generative method. Fig D. **Comparison of pLDDT for protected residues vs. non-protected residues for all four representative enzymes from** **Fig 3, as a function of sampling temperature.** As discussed in the main text, lower temperatures result in higher pLDDT values, with a slight deterioration for non-protected residues due to randomly occurring structural shifts from more confident alpha helices or beta sheets to less confident random coils. Table B. **Structural conservation of the active-site residues from a sample of mutants generated using our MC sampling approach.** For 13 representative enzymes (4 in the main text plus an additional 9), the table reports the protected site residues, the RMSD of those residues between the parent and three mutant examples (**S1–S3**), and the corresponding sequence similarities. Protected residues have been chosen to represent those corresponding to the catalytic core of the enzyme. In all systems, the structural deviation in the catalytic core remains extremely small, with a mean RMSD across all MC-sampled structures over the 13 enzymes of 0.355Å, despite having sequence identities as low as 2%. These data illustrate that our generative strategy can explore distant regions of sequence space while preserving the geometry of the active site, thereby enriching the pool of mutants that are immediately amenable to experimental screening. Fig E. **Sensitivity of MC sampling to the number of simultaneous mutations per proposal step.** Each row corresponds to one enzyme: oxidoreductase (1A2J), cellulase (1EDG), hydrolase (1UA7), and Taq polymerase (1TAQ). Left column: embedding energy $E_{m}$ (Eq. 1, log scale) vs MC step. Right column: sequence identity (%) relative to the wild type vs MC step. Line shading indicates the number of mutations per step (*n* = 1,2,3,5), from dark to light. All runs use *T* = 10^-4^. Fig F. **BLOSUM62 vs pLM comparison for oxidoreductase (1A2J), cellulase (1EDG), hydrolase (1UA7), and Taq polymerase (1TAQ).** Left column: BLOSUM62 energy evolution (*E*_BLOSUM_ defined in Eq. 5) over MC steps at six temperatures, showing mean ± standard deviation across five independent repeats. Right column: 2D contour plot of RMSD of protected residues vs. sequence identity, comparing pLM (blue) and BLOSUM62 (green) sampling. Contours are estimated via Gaussian kernel density estimation (KDE) on paired (log_10_ RMSD, sequence identity) values from the final 2,500 MC steps. Dashed lines trace the mode trajectory connecting the 2D KDE peak at each temperature. Fig G. **DCA vs pLM comparison for oxidoreductase (1A2J), cellulase (1EDG), and hydrolase (1UA7).** Left column: DCA Potts Hamiltonian energy evolution (*E*_DCA_ defined in Eq. 6) over MC steps at six temperatures, showing mean ± standard deviation across five independent repeats. Right column: 2D contour plot of RMSD of protected residues vs. sequence identity, comparing pLM (blue) and DCA (red) sampling. Contours are estimated via Gaussian kernel density estimation (KDE) on paired (log_10_ RMSD, sequence identity) values from the final 2,500 MC steps. Dashed lines trace the mode trajectory connecting the 2D KDE peak at each temperature. Fig H. **Molecular dynamics simulations of representative mutants generated at different sampling temperatures for all remaining enzymes considered in this work.** Left panel: time evolution of the RMSD (see Eq. 3 in the main text). Right panel: RMSF (see Eq. 4 in the main text) averaged over time, for each residue; shaded regions indicate protected residues (i.e., those belonging to the putative catalytic site and thus preserved during the generative procedure). See main text for further details. Fig I **Molecular dynamics simulations of representative mutants (continued).** RMSD (left column, Eq. 3) and RMSF (right column, Eq. 4) for enzymes 1TAQ, 1TCA, 1THX, and 1UA7. See Fig H for full caption details. Fig J. **Molecular dynamics simulations of representative mutants (continued).** RMSD (left column, Eq. 3) and RMSF (right column, Eq. 4) for enzymes 1ZG4, 4M6K, and 4RQR. See Fig H for full caption details. Fig K. **Embedding energy distributions for synthetic and random variants based on the experimental dataset from Russ et al. [30].** Left: distribution of embedding energy $E_{m}$ for synthetic variants (split by functional/non-functional classification) and randomly generated mutants. Right: scatter plot of sequence identity vs. embedding energy $E_{m}$. Notably, low embedding energy in the random mutants arises purely from high sequence similarity, whereas functional synthetic variants achieve low $E_{m}$ across a much broader range of sequence identities. Fig K. **Cumulative distribution function (CDF) of embedding energy** $E_{m}$ **for the synthetic and random variant groups shown in Fig K.** The CDF compares functional synthetic variants, non-functional synthetic variants, and randomly generated mutants. The key threshold $E_{m}=0.05$ is marked; 98.7% of functional synthetic variants fall below this value, whereas only 41.8% of random mutants do so, and those with $E_{m}<0.05$ have uniformly high sequence identity to the reference. Fig M. **Classification performance of the embedding energy** $E_{m}$ **on the synthetic variants from Russ et al. [30].** The 999 synthetic chorismate mutase variants are split into functional (*n* = 398, NRE>0.42) and non-functional (*n* = 601) classes, with $E_{m}$ (against the closest natural functional reference) used as the classifier score and predicted-positive corresponding to $E_{m}<0.05$. **(a)** Confusion matrix at $E_{m}<0.05$ (raw counts and row-normalised percentages): recall = 98.7% (95% bootstrap CI [0.976, 0.997]), precision = 50.2% ([0.469, 0.534]), specificity = 35.1%, FPR = 64.9%. The threshold thus acts as a high-sensitivity rule-out filter: high $E_{m}$ is strong evidence of non-functionality, whereas low $E_{m}$ is necessary but not sufficient. **(b)** ROC curve obtained by sweeping the threshold on $E_{m}$: AUC = 0.796 (95% bootstrap CI [0.768, 0.823], 1000 resamples). The red operating points correspond to $E_{m}<0.05$ and $E_{m}<0.15$; the latter saturates recall at 1.000 for FPR = 97.5%.(PDF)
