# Supplementary material for: Mind the gap: An embedding guide to safely travel in sequence space
Source: PLoS Comput Biol. 2026 Jul 9;22(7):e1014433. doi: 10.1371/journal.pcbi.1014433 (PMC13375127; doi:10.1371/journal.pcbi.1014433)
Supplement: S1 Appendix — RMSD values are reported in Å, and pLDDT scores are presented in the range of [0,1], with 1 representing the highest confidence. RMSD and pLDDT assess the fidelity of predicted structures relative to experimentally determined conformations and the model confidence in the prediction, respectively. In 1TAQ, the high RMSD stems from the wrong predicted alignment of two domains, each of which, however, is internally described with very high accuracy. Fig A. Correlation between ESMFold [7] and AlphaFold2 [43] RMSD prediction for a random subset of mutants generated during our simulations. The subset was specifically chosen to sample a wide range of energies and RMSD of the active site. As somewhat expected, predictions are more highly correlated in the small RMSD region (≤ 1.5 Å), as shown in the inset on the right, which also generally corresponds to sequences of higher similarity with the original ones (as discussed in the main text). High-energy regions often correspond to almost random sequences, for which we do not expect a protein folding algorithm to work particularly well because of the lack of a reference system close enough for the inference process to provide a reliable result, as also shown by the drop in the confidence metric, see Fig B. However, such high-RMSD sequences are also irrelevant from a practical point of view, considering keeping the active site intact is required to preserve catalytic activity. Fig B. Correlation between ESMFold and AlphaFold2 pLDDT confidence scores for a random subset of mutants generated during our simulations. There is a strong correlation between the pLDDT from both models, which is evident from the clustering of points along the diagonal. Predictions for lower energy mutants tend to have a higher confidence. The same subset from Fig A was used for this analysis. Fig C. Comparison of RMSD for protected residues vs. non-protected residues for all four representative enzymes from Fig 3, as a function of sampling temperature. [file pcbi.1014433.s001.pdf]

# Supplementary Information

## A Comparison between ESMFold and AlphaFold2

Figure A compares structural predictions from ESMFold and AlphaFold2 across 30 mutants for each of the four enzymes discussed in the main text. The right panel highlights the low-RMSD regime in the left panel. In most cases, low-energy structures that are predicted to have a low RMSD with ESMFold are also confirmed to have a low RMSD by AlphaFold2.

As shown in Fig B, structures with low predicted energy also exhibit high confidence scores (pLDDT) in both models, with a strong correlation evident from the clustering of points along the diagonal. This consistency further supports the reliability of low-energy, low-RMSD predictions; precisely the class of structures most relevant for practical applications in enzyme design, where preserving the geometry of the catalytic site is critical.

It is worth noting, however, that the data is slightly biased in favour of ESMFold, as ESMFold-derived embeddings were used during mutant selection. This may account for the marginally lower RMSD and higher pLDDT values observed in ESMFold predictions relative to AlphaFold2.

Table A: **Structural prediction accuracy between ESMFold and AlphaFold2 for the reference sequence of the enzymes discussed in the main text.** RMSD values are reported in Å, and pLDDT scores are presented in the range of [0,1], with 1 representing the highest confidence. RMSD and pLDDT assess the fidelity of predicted structures relative to experimentally determined conformations and the model confidence in the prediction, respectively. In 1TAQ, the high RMSD stems from the wrong predicted alignment of two domains, each of which, however, is internally described with very high accuracy.

| PDB Code | ESMFold | | AlphaFold2 | |
|---|---|---|---|---|
| | RMSD (Å) | pLDDT | RMSD (Å) | pLDDT |
| 1A2J | 2.59 | 0.941 | 2.76 | 0.961 |
| 1EDG | 1.42 | 0.931 | 1.47 | 0.970 |
| 1UA7 | 1.22 | 0.930 | 1.36 | 0.978 |
| 1TAQ | 30.3 | 0.874 | 33.7 | 0.905 |

## B RMSD and pLDDT outside the protected region

Fig C compares the RMSD of the protected residues with that of the rest of the enzyme, while Fig D reports the confidence metric for these predictions. We see for both the protected and unprotected regions, lower sampling temperatures lead to lower distortions, while the local structure of the protected region is always better maintained, with markedly lower RMSD. This result is a feature of our procedure, which only biases sampling to guarantee conservation of the environment for those residues specifically defined by the users, while allowing the rest of the enzyme to freely sample structural diversity. At the same time, it should also be noted that confidence metrics, such as pLDDT, also invariably deteriorate outside the protected region. However, this is a reflection of the fact that the relatively high randomness of the sequence outside the protected region leads more naturally to random coils rather than alpha helices or beta sheets, whose dynamical, unstructured nature is inherently associated to lower pLDDT values.

## C Additional library of variants generated in this study

To show the generality of our approach, we apply our generative procedure on a larger set of 13 different enzymes, covering different folds and families. This library of over 12,500 sequences (all with RMSD smaller than 2Å) was curated and validated through structure prediction and is available at `https://doi.org/10.5281/zenodo.15696797` to support further experimental testing and integration into directed evolution pipelines. Selected results from this library are reported in Table B.

## D Sensitivity analysis to the number of mutations

In the main text, our MC sampling proposes single-point mutations at each step. However, single-residue proposals may be susceptible to epistatic barriers, where mutating one residue at a time traps the sampler in local minima that

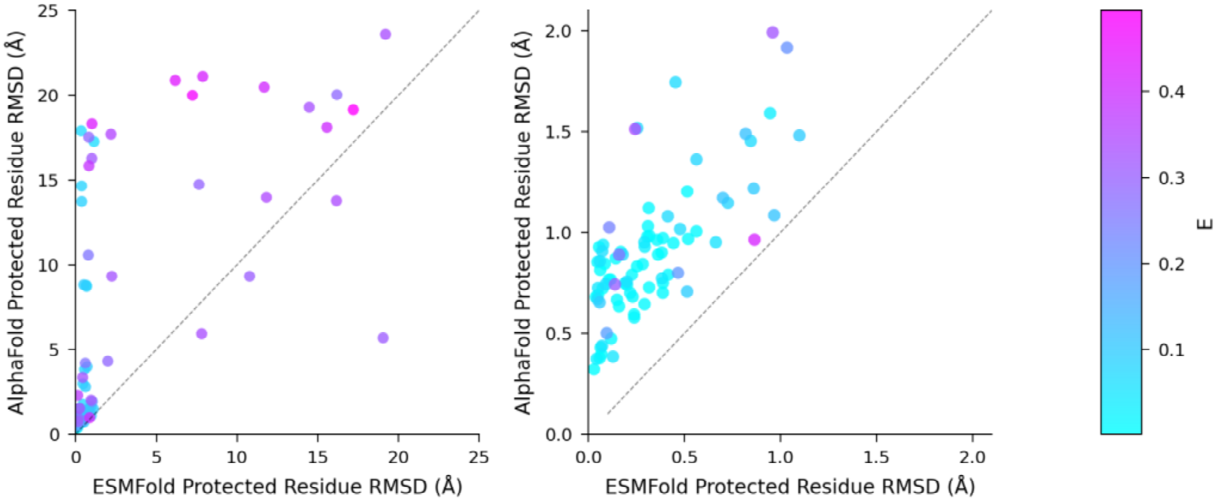


Fig A: **Correlation between ESMFold [7] and AlphaFold2 [43] RMSD prediction for a random subset of mutants generated during our simulations**. The subset was specifically chosen to sample a wide range of energies and RMSD of the active site. As somewhat expected, predictions are more highly correlated in the small RMSD region ($\leq$ 1.5 Å), as shown in the inset on the right, which also generally corresponds to sequences of higher similarity with the original ones (as discussed in the main text). High-energy regions often correspond to almost random sequences, for which we do not expect a protein folding algorithm to work particularly well because of the lack of a reference system close enough for the inference process to provide a reliable result, as also shown by the drop in the confidence metric, see Fig B. However, such high-RMSD sequences are also irrelevant from a practical point of view, considering keeping the active site intact is required to preserve catalytic activity.

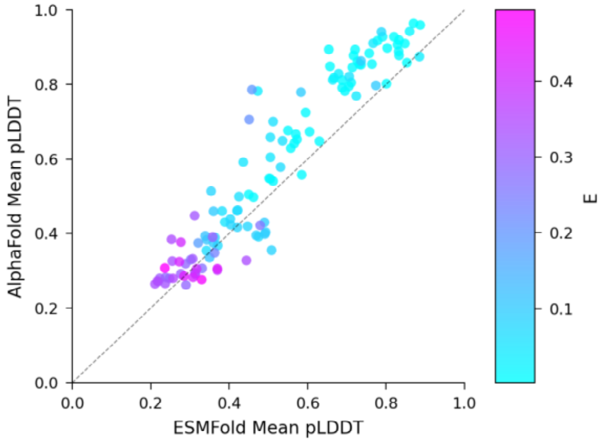


Fig B: **Correlation between ESMFold and AlphaFold2 pLDDT confidence scores for a random subset of mutants generated during our simulations**. There is a strong correlation between the pLDDT from both models, which is evident from the clustering of points along the diagonal. Predictions for lower energy mutants tend to have a higher confidence. The same subset from Fig A was used for this analysis.

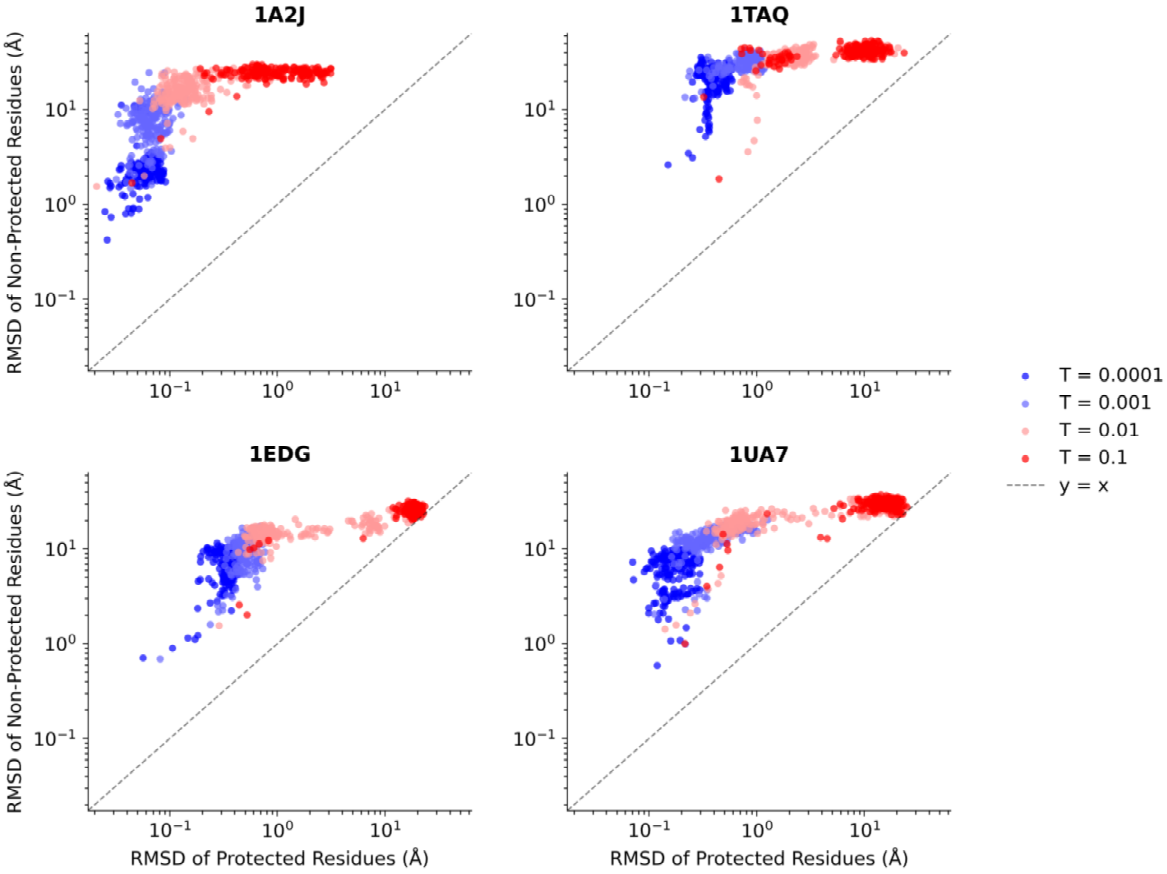


Fig C: **Comparison of RMSD for protected residues vs. non-protected residues for all four representative enzymes from Fig 3, as a function of sampling temperature.** In general, lower temperatures correspond to lower RMSD values for the entire enzyme. However, the RMSD of the protected residues is always lower due to the residue preservation nature of our generative method.

could be escaped by simultaneous multi-site changes. From a statistical mechanics perspective, the sampling could break *practical ergodicity*, i.e., not being able to reach all the microstates of the sequence space in a *practically* long enough simulation. To investigate this, we repeat the sampling experiments for all four enzymes – oxidoreductase (1A2J), cellulase (1EDG), hydrolase (1UA7), and Taq polymerase (1TAQ) – at $T = 10^{-4}$, but vary the number of simultaneous substitutions proposed per MC step: $n \in \{1, 2, 3, 5\}$.

Figure E shows the embedding energy $E_m$ and sequence identity relative to the wild type over 10,000 MC steps. As $n$ increases, the acceptance rate drops sharply: for 1A2J, acceptance rates are approximately 20%, 6%, 2%, and 0.1% for $n = 1, 2, 3, 5$, respectively. This is expected, since proposing multiple simultaneous mutations increases the probability that at least one substitution is energetically unfavourable, leading to rejection of the entire proposal. Consequently, trajectories with higher $n$ show slower exploration and more abrupt energy jumps when proposals are accepted. Based on these results, single-point mutations thus seem to equilibrate the fastest, suggesting the system is able to traverse epistatic barriers in the energy/fitness landscape. Regardless of the choice of $n$, in a sufficient amount of time, the system equilibrates to the same energy level, and thus we assume that it samples the same distribution of energies. This part of the investigation used the pLM-guided MC implementation from BAGEL [50].

## E Embedding-based approach against BLOSUM sampling

As a first baseline, we consider an independent-site model based on the BLOSUM62 substitution matrix [51]. Unlike DCA benchmarked later, which captures pairwise coevolutionary couplings between residue positions, BLOSUM62

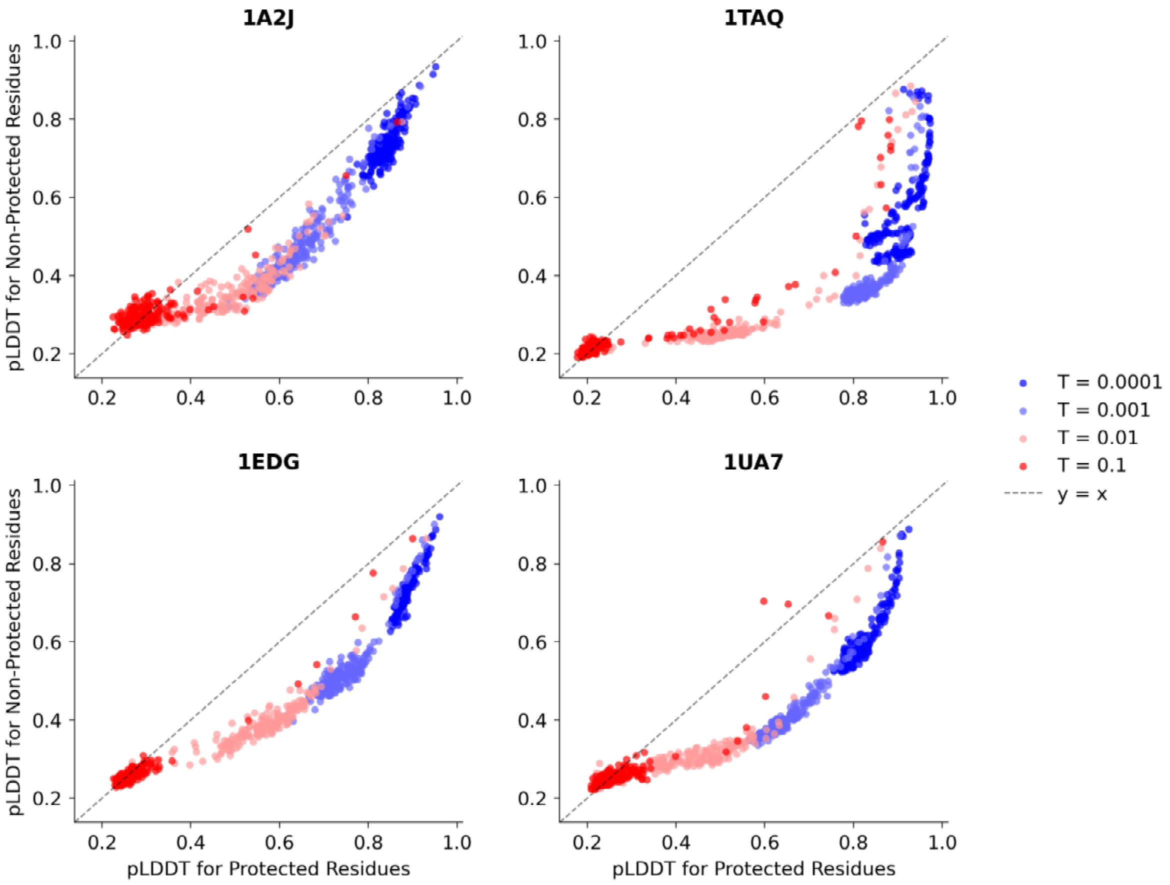


Fig D: **Comparison of pLDDT for protected residues vs. non-protected residues for all four representative enzymes from Fig 3, as a function of sampling temperature.** As discussed in the main text, lower temperatures result in higher pLDDT values, with a slight deterioration for non-protected residues due to randomly occurring structural shifts from more confident alpha helices or beta sheets to less confident random coils.

encodes only single-site substitution preferences derived from aligned protein blocks. This makes it an informative control: any difference in the structural-preservation behaviour of pLM-guided versus BLOSUM62-guided sampling cannot be attributed to evolutionary substitution statistics alone, but must reflect the higher-order sequence context captured by the language model.

We define the BLOSUM62 energy of a mutant sequence $\boldsymbol{\sigma}$ relative to the wild-type sequence $\boldsymbol{\sigma}^0$ as:

$$E_{\text{BLOSUM}}(\boldsymbol{\sigma}) = -\sum_{i=1}^{L} B(\sigma_i^0, \sigma_i) \tag{5}$$

where $B(\sigma_i^0, \sigma_i)$ is the BLOSUM62 log-odds score for substituting amino acid $\sigma_i^0$ with $\sigma_i$ at position $i$. The negation ensures that conservative substitutions (high BLOSUM62 scores) correspond to low energy.

We run this energy function through the same MC optimisation loop as for the pLM approach, sweeping through a range of temperatures, for all four enzymes: oxidoreductase (1A2J), cellulase (1EDG), hydrolase (1UA7), and Taq polymerase (1TAQ). Fig F shows the comparison between the BLOSUM62 and pLM-guided sampling.

## F Embedding-based approach against DCA sampling

As a second baseline, we carry out DCA sampling. We compute the parameters using the EVcouplings web server [52] for three enzymes from the main body, oxidoreductase (1A2J), cellulase (1EDG) and hydrolase (1UA7). Taq poly-

Table B: **Structural conservation of the active-site residues from a sample of mutants generated using our MC sampling approach.** For 13 representative enzymes (4 in the main text plus an additional 9), the table reports the protected site residues, the RMSD of those residues between the parent and three mutant examples (**S1–S3**), and the corresponding sequence similarities. Protected residues have been chosen to represent those corresponding to the catalytic core of the enzyme. In all systems, the structural deviation in the catalytic core remains extremely small, with a mean RMSD across all MC-sampled structures over the 13 enzymes of 0.355Å, despite having sequence identities as low as 2%. These data illustrate that our generative strategy can explore distant regions of sequence space while preserving the geometry of the active site, thereby enriching the pool of mutants that are immediately amenable to experimental screening.

| **PDB ID** | **Class** | **Protected Residues, $\{P\}$** | **Protected RMSD (Å)** | | | **Sequence Identity (%)** | | |
|---|---|---|---|---|---|---|---|---|
| | | | **S1** | **S2** | **S3** | **S1** | **S2** | **S3** |
| 1A2J | Oxidoreductase | C30, C33 | 0.021 | 0.270 | 0.137 | 79 | 2 | 5 |
| 1A58 | Isomerase | R66, F71, M72, Q74, G83, A112, N113, K114, Q122, F124, H132, L133, H137 | 0.119 | 0.434 | 0.446 | 95 | 19 | 20 |
| 1AEC | Hydrolase | C25, H162 | 0.012 | 0.184 | 0.217 | 92 | 6 | 6 |
| 1EDG | Cellulase | R79, H122, N169, E170, H254, Y256, E307 | 0.057 | 0.480 | 0.422 | 96 | 10 | 14 |
| 1TAQ | Taq polymerase | D610, I614, E615, F667, Y671, K663, R659 | 0.130 | 0.425 | 0.479 | 95 | 25 | 27 |
| 1TCA | Hydrolase | S105, D187, H224, T40, Q106 | 0.042 | 0.500 | 0.379 | 91 | 6 | 9 |
| 1THX | Oxidoreductase | W36, C37, G38, P39, C40 | 0.037 | 0.353 | 0.471 | 50 | 10 | 9 |
| 1UA7 | Hydrolase | D173, E205, D266 | 0.070 | 0.476 | 0.416 | 29 | 6 | 10 |
| 1ZG4 | Hydrolase | S45, K48, S105, E141, N145 | 0.020 | 0.483 | 0.476 | 98 | 17 | 19 |
| 2PPN | Isomerase | H25, Y26, F36, F46, W59, Y80, Y82, H87, F99 | 0.028 | 0.481 | 0.490 | 97 | 35 | 37 |
| 4M6K | Oxidoreductase | E31, R71, F35, N65 | 0.034 | 0.388 | 0.386 | 98 | 22 | 21 |
| 4RQR | Oxidoreductase | C26, C29 | 0.008 | 0.199 | 0.468 | 83 | 2 | 2 |
| 9PAP | Hydrolase | C25, H159, N175, Q19, W177 | 0.030 | 0.496 | 0.451 | 97 | 15 | 16 |

merase (1TAQ) is too long to be sent to the web server. The server fits model parameters at four different bitscore thresholds and ranks them; we select the recommended model for each enzyme (bitscores 0.3, 0.1, 0.1 for 1A2J, 1EDG and 1UA7, respectively). After loading them locally with the `EVcouplings` Python package [52], we effectively have a Potts model [53] of the form:

$$E_{\text{DCA}}(\boldsymbol{\sigma}) = -\sum_i h_i(\sigma_i) - \sum_{i<j} J_{ij}(\sigma_i, \sigma_j) \tag{6}$$

where $\boldsymbol{\sigma} = (\sigma_1, \ldots, \sigma_L)$ is a protein sequence of length $L$, $\sigma_i$ denotes the amino acid at position $i$, $h_i(\sigma_i)$ are local fields capturing single-site amino acid preferences, and $J_{ij}(\sigma_i, \sigma_j)$ are pairwise couplings encoding residue-residue coevolutionary constraints. The parameters are inferred from the MSA via pseudo-likelihood maximisation [54]. We sample through this in the same MC optimisation loop as in the pLM one above, sweeping through a range of temperatures.

Fig G shows the comparison between the DCA and pLM-guided sampling, with a consistent behaviour across all the three enzymes. Firstly, we see that similarly to the pLM approach, DCA sampling at various temperatures leads to equilibration at different energy levels, with two lowest temperature actually equilibrating at a lower energy than that of the wild type. Note that the magnitude of the energies should not be compared directly with the pLM approach. Second, in terms of structural preservation of the protected residues, we observe that the pLM approach reaches much lower sequence identity than the DCA approach at comparable, or even lower, RMSD.

# G Molecular Dynamics study of different enzyme mutants

For completeness, in the following section we report the results of the same analysis of Molecular Dynamics trajectories as described in Section 2.4 of the main manuscript, for each of the 13 enzyme studied (minus the two already described in the main text). Qualitatively, this analysis confirms the result already discussed: mutants generated at low MC-sampling temperature not only remain folded over the whole trajectory (as confirmed by the low RMSF for residues in the active site), but remain folded in a structure which is very close to that of the active site, as one can

evince from the low RMSD (centred around the average structure of the wild-type mutant). Results are shown in Figs. H–J.

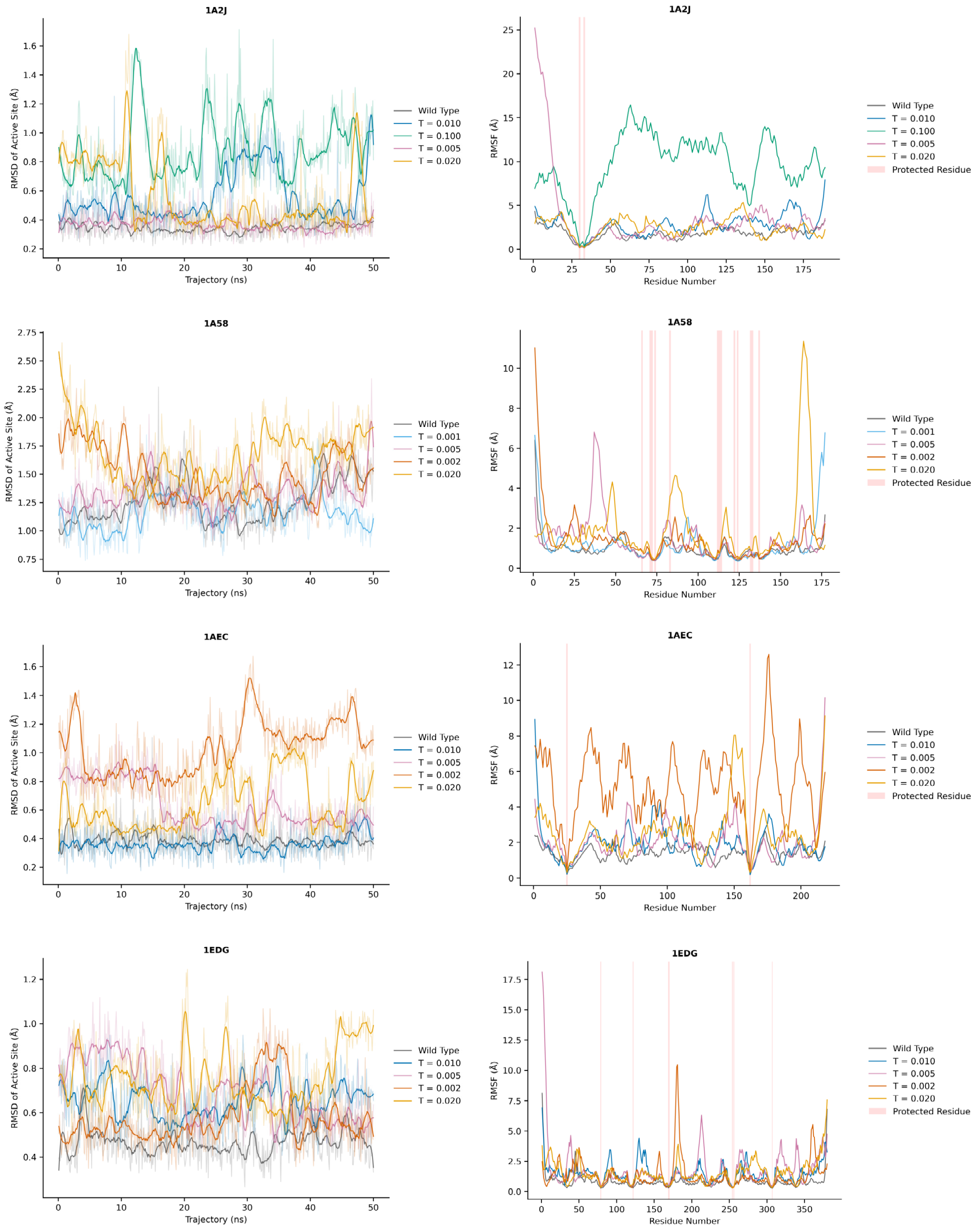


Fig H: **Molecular dynamics simulations of representative mutants generated at different sampling temperatures for all remaining enzymes considered in this work.** Left panel: time evolution of the RMSD (see Eq. 3 in the main text). Right panel: RMSF (see Eq. 4 in the main text) averaged over time, for each residue; shaded regions indicate protected residues (i.e., those belonging to the putative catalytic site and thus preserved during the generative procedure). See main text for further details.

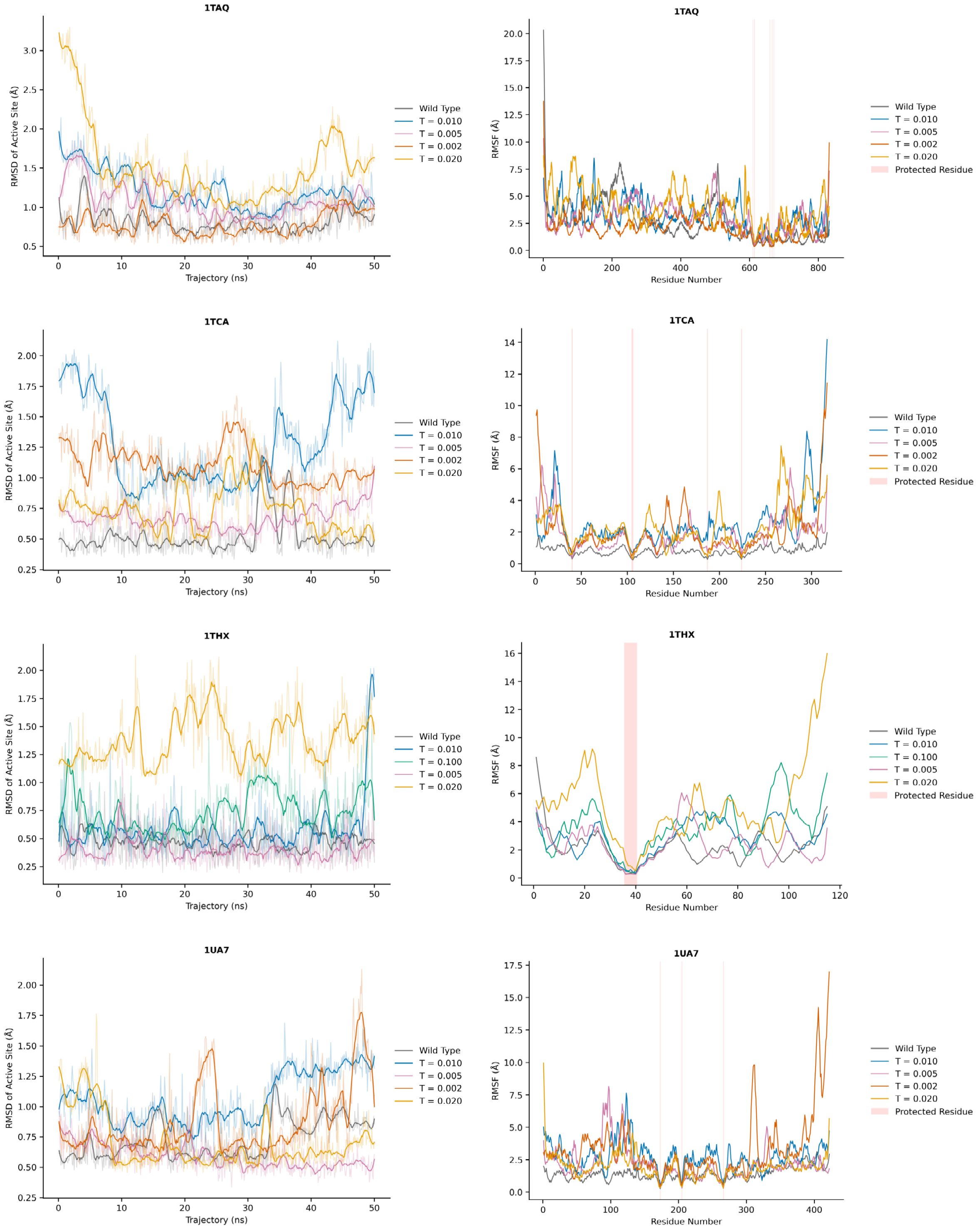


Fig I: **Molecular dynamics simulations of representative mutants (continued).** RMSD (left column, Eq. 3) and RMSF (right column, Eq. 4) for enzymes 1TAQ, 1TCA, 1THX, and 1UA7. See Fig H for full caption details.

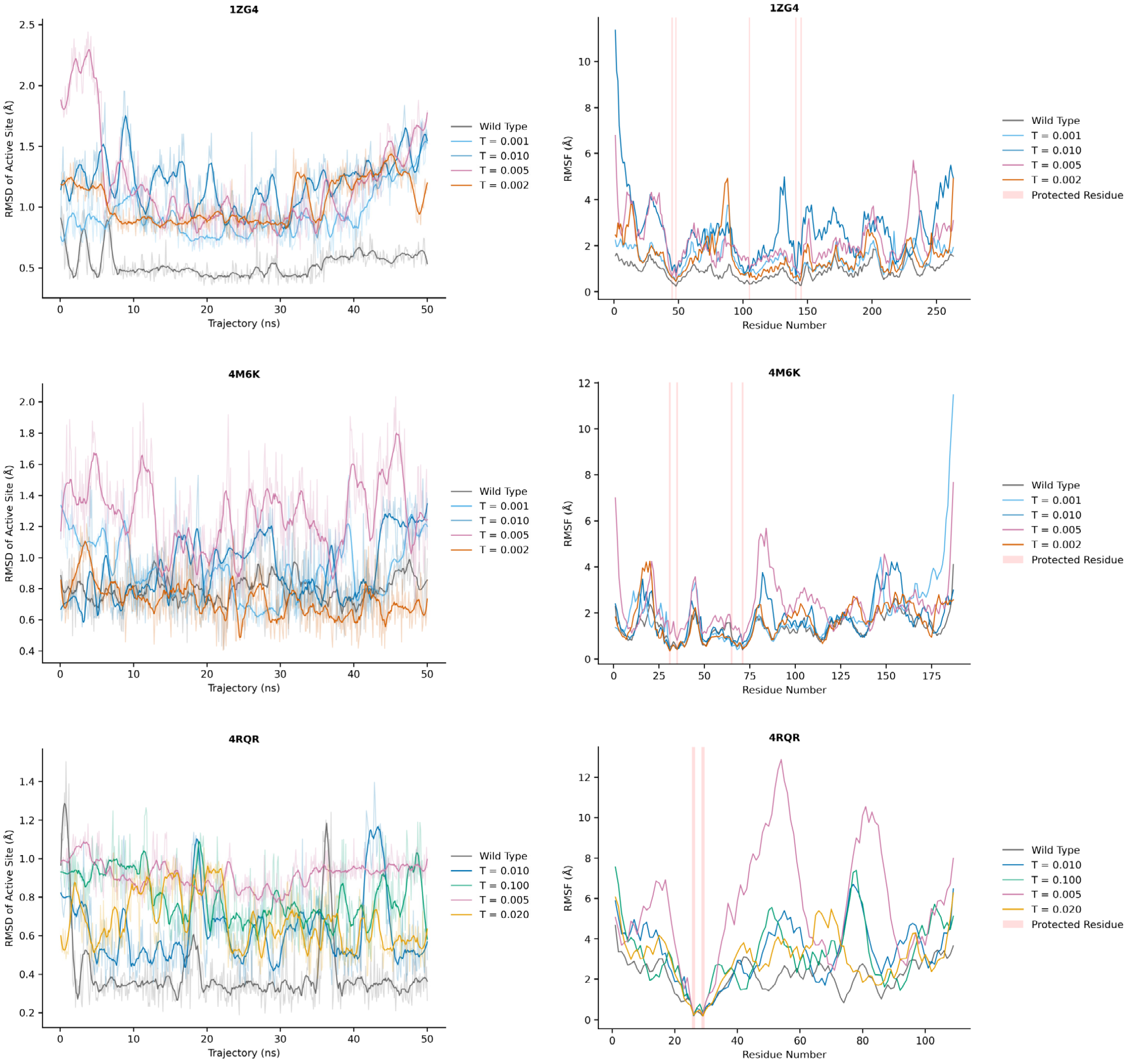


Fig J: **Molecular dynamics simulations of representative mutants (continued).** RMSD (left column, Eq. 3) and RMSF (right column, Eq. 4) for enzymes 1ZG4, 4M6K, and 4RQR. See Fig H for full caption details.

# H Comparison of variants from Russ et al. (2020) vs randomly generated samples

The full distributions supporting the analysis described in Section 2.5 of the main text are shown in Figs. K and L. The experimental dataset is from Russ et al. [30]. To complement this analysis, we also quantify the performance of the embedding energy $E_m$ as a binary classifier of functional vs. non-functional synthetic variants in Fig M, using lower $E_m$ as evidence for functionality and reporting the confusion matrix at the threshold $E_m = 0.05$ used in the main text alongside the full ROC curve.

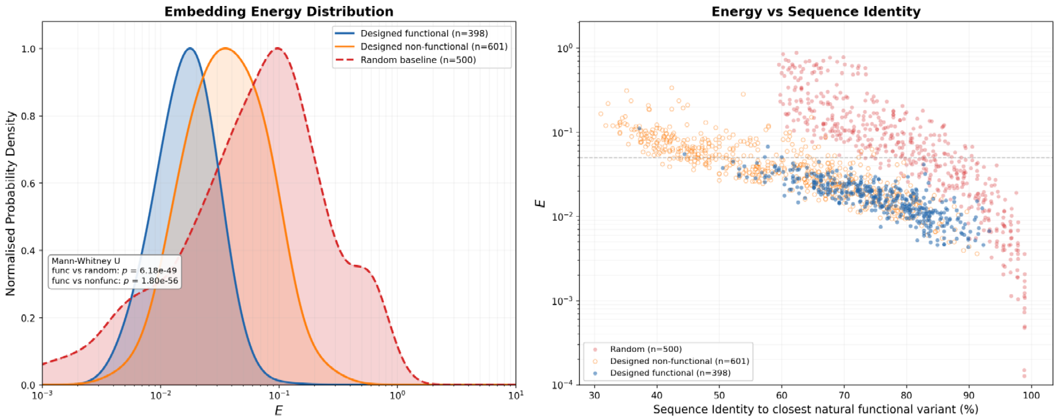


Fig K: **Embedding energy distributions for synthetic and random variants based on the experimental dataset from Russ et al. [30].** Left: distribution of embedding energy $E_m$ for synthetic variants (split by functional/non-functional classification) and randomly generated mutants. Right: scatter plot of sequence identity vs. embedding energy $E_m$. Notably, low embedding energy in the random mutants arises purely from high sequence similarity, whereas functional synthetic variants achieve low $E_m$ across a much broader range of sequence identities.

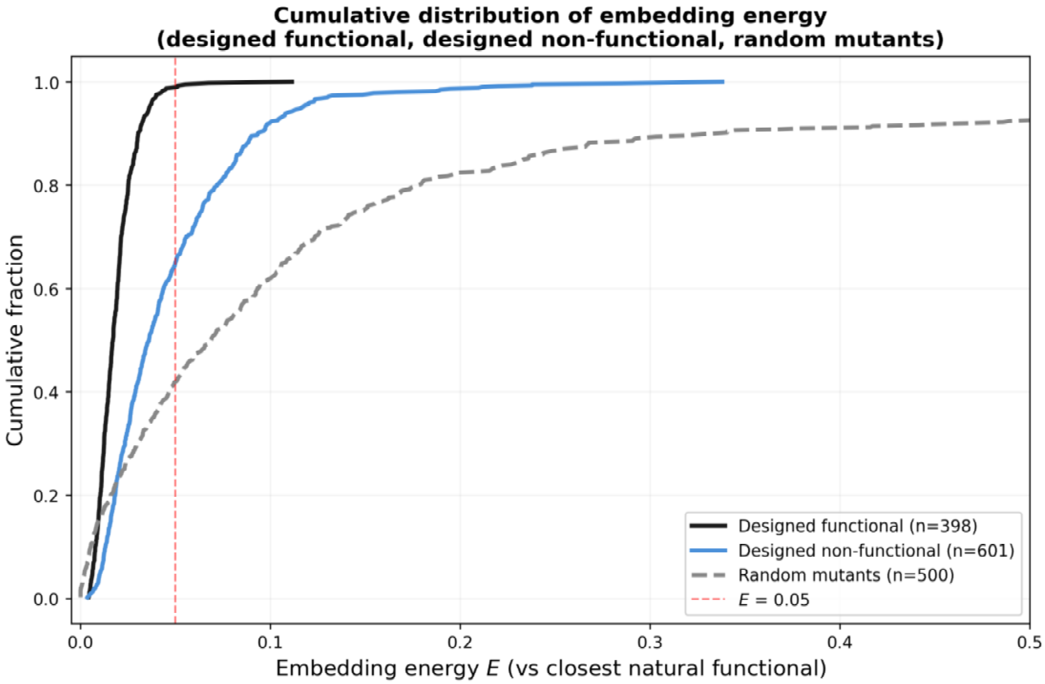


Fig L: **Cumulative distribution function (CDF) of embedding energy $E_m$ for the synthetic and random variant groups shown in Fig K.** The CDF compares functional synthetic variants, non-functional synthetic variants, and randomly generated mutants. The key threshold $E_m = 0.05$ is marked; 98.7% of functional synthetic variants fall below this value, whereas only 41.8% of random mutants do so, and those with $E_m < 0.05$ have uniformly high sequence identity to the reference.

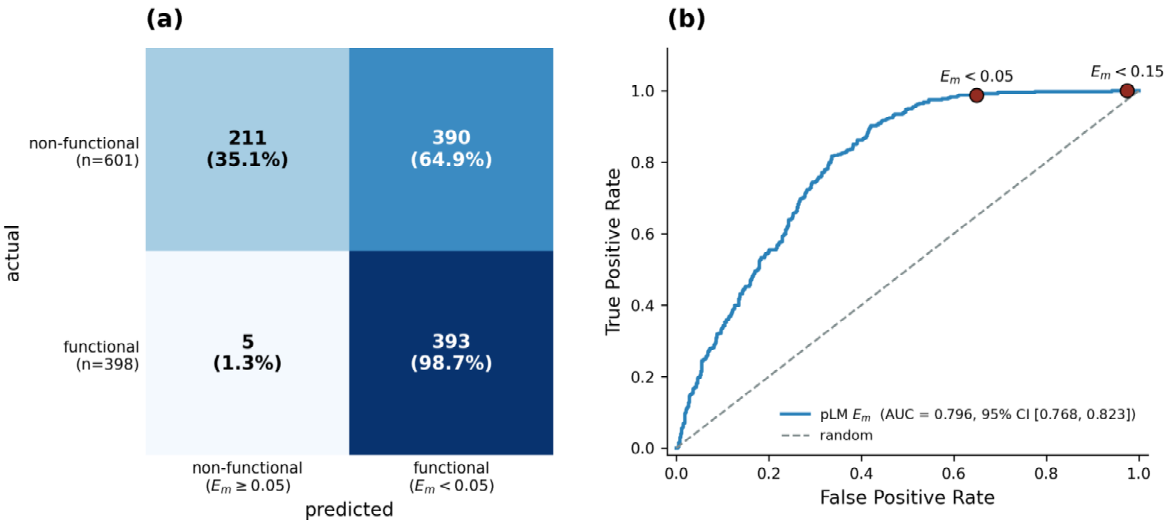


Fig M: **Classification performance of the embedding energy $E_m$ on the synthetic variants from Russ et al. [30].** The 999 synthetic chorismate mutase variants are split into functional ($n = 398$, $\mathrm{NRE} > 0.42$) and non-functional ($n = 601$) classes, with $E_m$ (against the closest natural functional reference) used as the classifier score and predicted-positive corresponding to $E_m < 0.05$. **(a)** Confusion matrix at $E_m < 0.05$ (raw counts and row-normalised percentages): recall $= 98.7\%$ (95% bootstrap CI $[0.976, 0.997]$), precision $= 50.2\%$ ($[0.469, 0.534]$), specificity $= 35.1\%$, FPR $= 64.9\%$. The threshold thus acts as a high-sensitivity rule-out filter: high $E_m$ is strong evidence of non-functionality, whereas low $E_m$ is necessary but not sufficient. **(b)** ROC curve obtained by sweeping the threshold on $E_m$: AUC $= 0.796$ (95% bootstrap CI $[0.768, 0.823]$, 1000 resamples). The red operating points correspond to $E_m < 0.05$ and $E_m < 0.15$; the latter saturates recall at $1.000$ for FPR $= 97.5\%$.

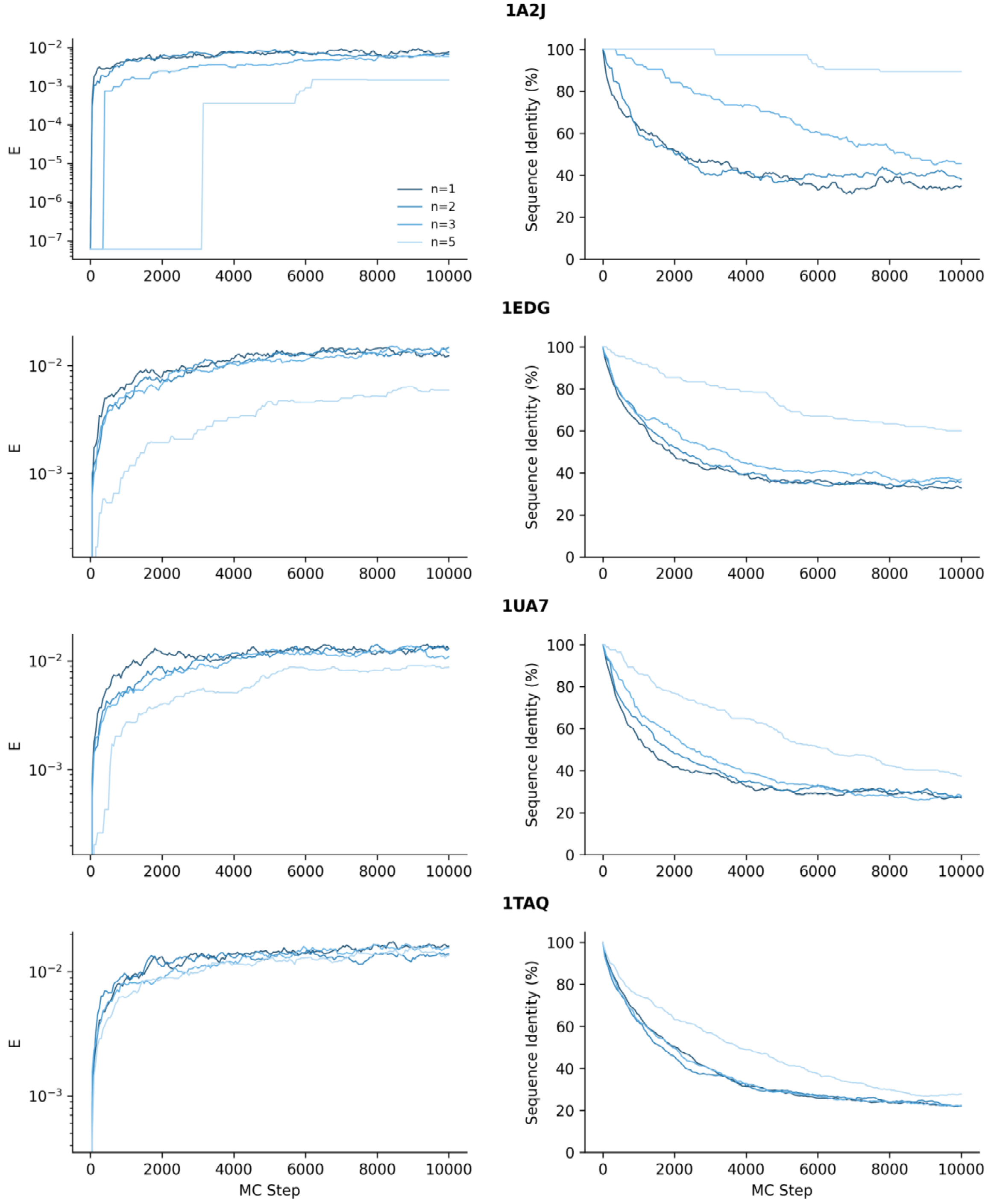


Fig E: **Sensitivity of MC sampling to the number of simultaneous mutations per proposal step.** Each row corresponds to one enzyme: oxidoreductase (1A2J), cellulase (1EDG), hydrolase (1UA7), and Taq polymerase (1TAQ). Left column: embedding energy $E_m$ (Eq. 1, log scale) vs MC step. Right column: sequence identity (%) relative to the wild type vs MC step. Line shading indicates the number of mutations per step ($n = 1, 2, 3, 5$), from dark to light. All runs use $T = 10^{-4}$.

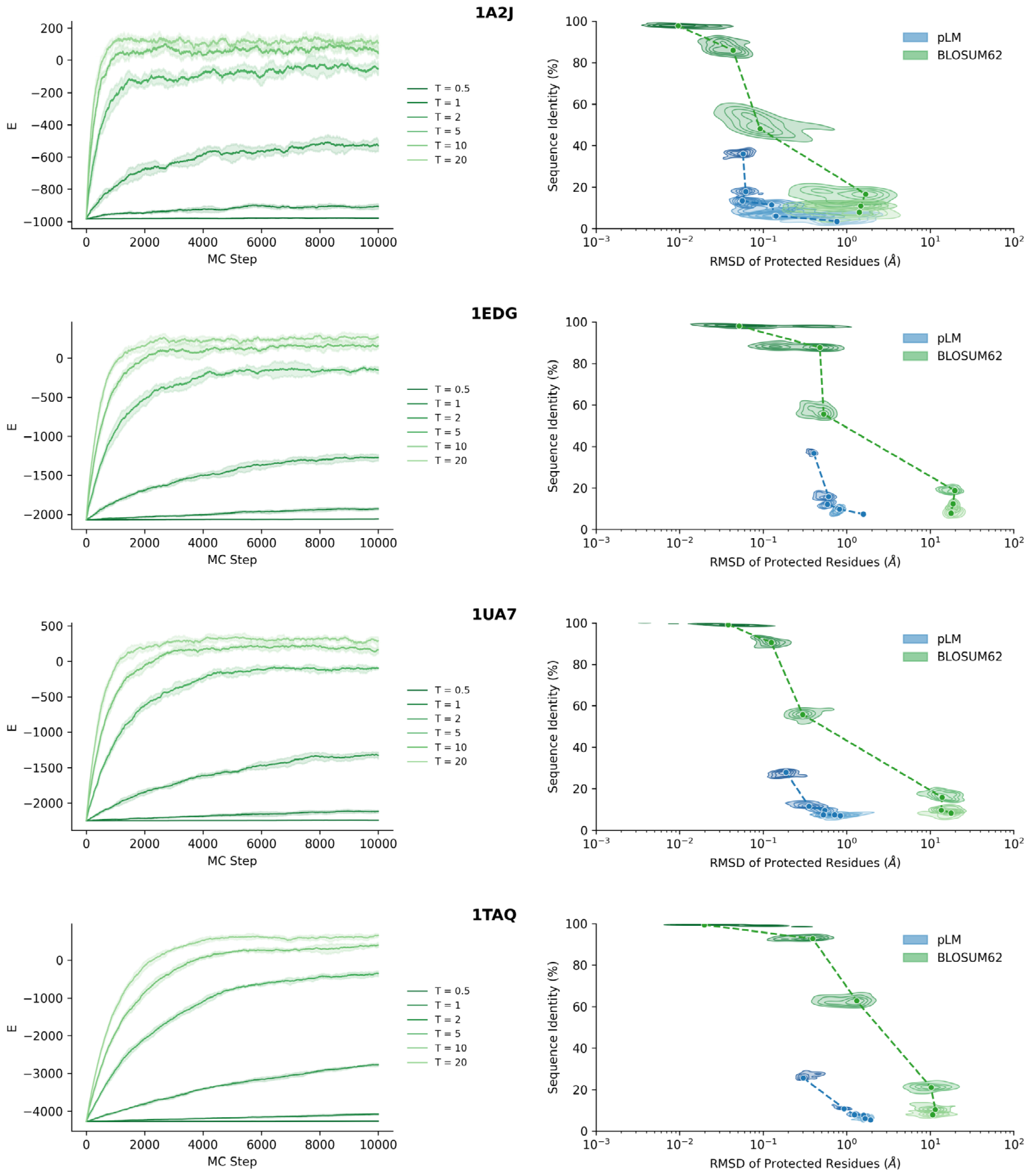


Fig F: **BLOSUM62 vs pLM comparison for oxidoreductase (1A2J), cellulase (1EDG), hydrolase (1UA7), and Taq polymerase (1TAQ).** Left column: BLOSUM62 energy evolution ($E_{\mathrm{BLOSUM}}$ defined in Eq. 5) over MC steps at six temperatures, showing mean $\pm$ standard deviation across five independent repeats. Right column: 2D contour plot of RMSD of protected residues vs. sequence identity, comparing pLM (blue) and BLOSUM62 (green) sampling. Contours are estimated via Gaussian kernel density estimation (KDE) on paired ($\log_{10}$ RMSD, sequence identity) values from the final 2,500 MC steps. Dashed lines trace the mode trajectory connecting the 2D KDE peak at each temperature.

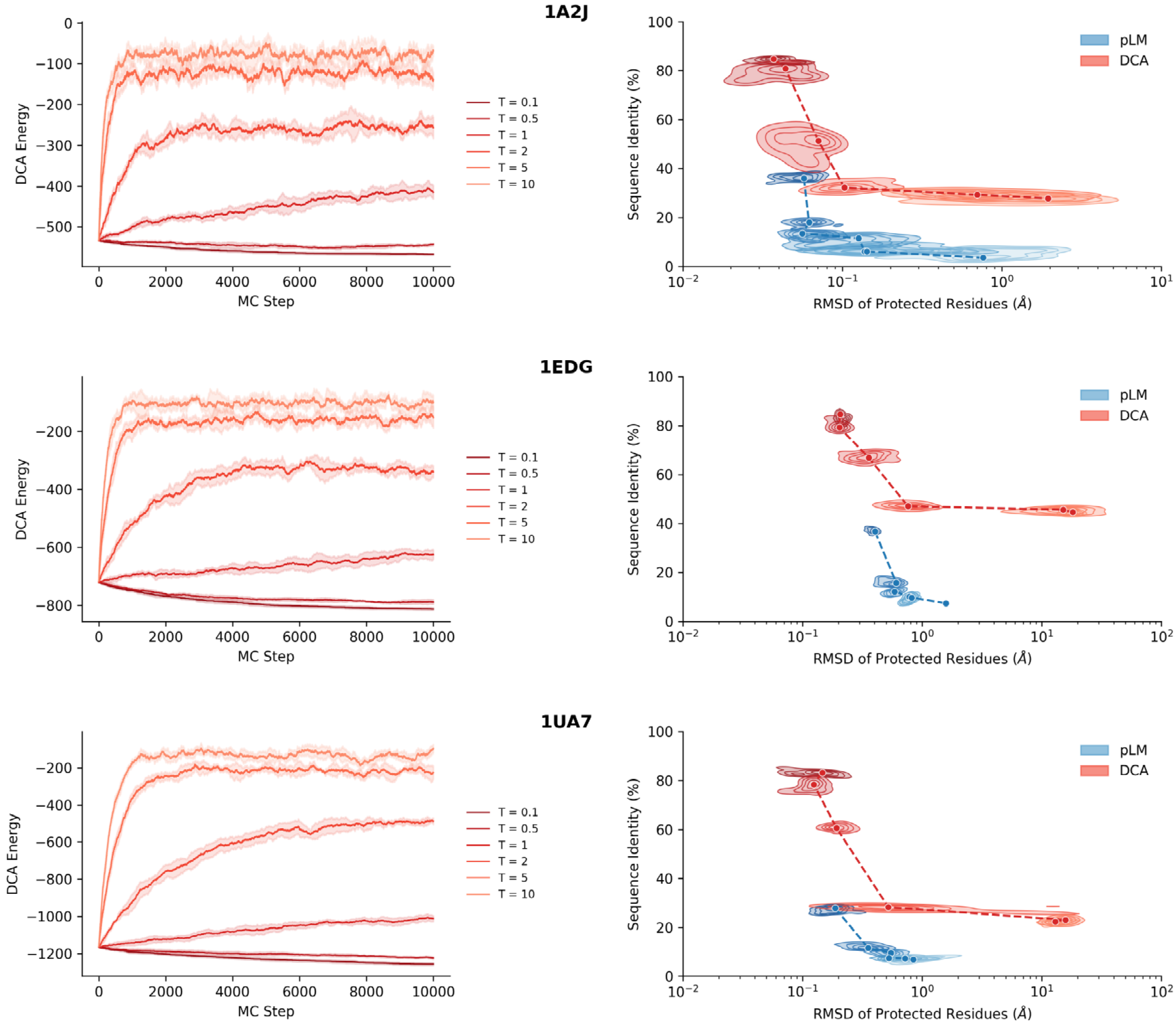


Fig G: **DCA vs pLM comparison for oxidoreductase (1A2J), cellulase (1EDG), and hydrolase (1UA7).** Left column: DCA Potts Hamiltonian energy evolution ($E_{DCA}$ defined in Eq. 6) over MC steps at six temperatures, showing mean $\pm$ standard deviation across five independent repeats. Right column: 2D contour plot of RMSD of protected residues vs. sequence identity, comparing pLM (blue) and DCA (red) sampling. Contours are estimated via Gaussian kernel density estimation (KDE) on paired ($\log_{10}$ RMSD, sequence identity) values from the final 2,500 MC steps. Dashed lines trace the mode trajectory connecting the 2D KDE peak at each temperature.
