# Supplementary material for: Mind the gap: An embedding guide to safely travel in sequence space
Source: PLoS Comput Biol. 2026 Jul 9;22(7):e1014433. doi: 10.1371/journal.pcbi.1014433 (PMC13375127; doi:10.1371/journal.pcbi.1014433)
Supplement: Attachment — Submitted filename: MindTheGapReply.pdf [file pcbi.1014433.s002.pdf]

Response to Reviewers' comments

We have addressed each of the reviewers' comments sequentially in the following response (our response highlighted in red). For your convenience, we have attempted to provide self-contained explanations of all modifications and additions here. However, please note that a complete understanding of the work and the new results, including new graphs, will require consulting the revised manuscript.

Reviewers' comments:

Reviewer #1: In this paper, the authors propose a generative algorithm for enzyme design that combines residue-level embeddings from ESM-2 with a Monte Carlo sampling framework. Their key idea is to define an "energy" function based on embedding similarity at user-defined protected residues (i.e., catalytic site residues) and to accept or reject mutations according to how much the embeddings of these residues are perturbed. **In this way, the algorithm is claimed to generate mutants that can differ drastically in global sequence identity (as low as 2–5% in Table S-2) while preserving the local geometry of the catalytic site with RMSD values consistently below 0.3 A (Figure 3).** They demonstrate this across 13 enzymes of diverse classes, highlighting examples such as Taq polymerase (1TAQ), oxidoreductases (1A2J, 4M6K), cellulases (1EDG), and hydrolases (1UA7). Structural coherence of the generated enzymes is validated in silico by ESMFold and AlphaFold2, with correlations between embedding "energy" and structural RMSD (Spearman rho = 0.85–0.93) as well as embedding energy and pLDDT (rho = –0.85 to –0.94) reported in Table II. The authors emphasize that their approach is interpretable and produces large mutant libraries (12,500 sequences per enzyme) with preserved active site geometry for future experimental screening.

The paper makes a strong case for interpretability and for the ability to bias generative protein design towards the conservation of catalytic residues. However, for me, the claims of methodological advance are undermined by two central weaknesses. **First, the work does not include any benchmarking against other sampling-based generative approaches for protein design, making it difficult to judge novelty or relative performance.**

Second, the validation is entirely computational and does not demonstrate that any of the generated enzymes are functional. For enzymes, structure is insufficient, and catalytic activity must be shown. Without addressing these two gaps, the manuscript does not yet provide evidence that this is a viable generative strategy for enzyme engineering.

Here are four concerns the authors need to address:

1. The authors need to add valid benchmarks. The obvious comparisons are latent diffusion on ESM-2 embeddings, including DiMA and AMP-Diffusion, as well as Gaussian perturbation of ESM-2 embeddings as done in PepPrCLIP for peptide design. The authors should compare their Monte Carlo sampling to these types of strategies on the same set of enzymes, reporting side-by-side results for catalytic-site RMSD, global RMSD, pLDDT distributions, sequence identity ranges, and sequence diversity metrics. Without this, it is impossible to tell whether the proposed method provides any advantage over existing latent generative frameworks.

We agree that placing our method in the context of existing generative frameworks is valuable. However, we note that the methods cited (DiMA, AMP-Diffusion, PepPrCLIP) were developed for different tasks and, to our knowledge, have not been applied or shown to lead to preservation of specific structural motifs in enzymes. A direct comparison is, therefore, not straightforward.
Instead, and as requested also by another reviewer, we compare to a different, well-established computational method previously used specifically for enzyme design, more precisely, Direct Coupling

Analysis. While the details can be read in the revised manuscript, the general message is that our pLM-based method outperforms DCA, in the sense, that it produces enzymes with either smaller sequence similarity to the original one (at a fixed value of the calculated RMSD w.r.t the wild-type), or for the same sequence similarity, enzymes that display a closer resemblance to the wild-type in terms of active site structure RMSD.

This comparison is now discussed in the revised manuscript, where we add:

"We highlight here that our method outperforms standard approaches for *in silico* directed evolution based on point or pairwise substitution correlations derived from Multiple Sequence Alignment, namely, sampling guided by BLOSUM-derived substitution energies or by Potts models fitted via mean-field Direct Coupling Analysis (see the Supplementary Information). Specifically, sampling based on our pLM-derived embedding energy (Eq.~\ref{eq:energy}) achieves higher sequence diversity while preserving the local geometry of the catalytic site closer to that of the original enzyme and, conversely, lower structural distortion for a given level of sequence diversity."

For reference, the full results are summarised in Sections E and F of the Supplementary Information, Figs. S-6 and S-7.

We also want to add another comment. A key distinction, and advantage, of our approach is the ability to explicitly conserve user-specified residues (e.g. known catalytic motifs) during the generative process. Methods operating in latent space do not have this control, and would require generating many variants and subsequently filtering for the presence of these motifs, adding computational overhead. Another advantage is that by using an energy definition that is bounded by below to do MC sampling mathematically implies very well known convergence results and guarantees full sampling of all the relevant structures. We are not aware of any paper showing such properties can be proven for latent space methods. More generally, we do not claim our method to be computationally superior (which would be hill-defined, and depending on the exact benchmark anyway) but we do show our method demonstrably generates variants that, when folded with established protein-folding algorithms, conserve the structure and geometry of any user-specified site — as shown in Figure 3 and quantified across the full benchmark in Table I. Because our approach generates hundreds of structurally conserved variants on a single standard GPU in a few hours, it can be reliably used to provide candidates for experimental testing without ever becoming a bottleneck in terms of design speed anyway, so these computational comparisons seem to us a mute point. A truly informative benchmark would ultimately require experimental validation across all methods on the same design goal, which is beyond the scope of the present work (we do, however, provide some comparison with experimental data, see later).

To strengthen our conclusions, instead, in the revised manuscript we now show that not only structural and chemical environments are conserved, but the same is true when considering the dynamics and flexibility of the enzyme, as confirmed by additional Molecular Dynamics simulations, the new section "Low embedding energy variants further preserve the local dynamics of the wild-type" and in particular, the new Fig 9 as well as additional material in the Supplementary Information. While we do not claim that this will necessarily lead to conservation of function, we believe our model will at least have a bias towards enzymes that preserve catalytic activity in the lab, as our method bias against enzymes that do not preserve the structure of catalytic motifs and, as we additionally show, also generates variants with similar dynamics and flexibility to the functional wild type.

2. Demonstrating that catalytic residues remain geometrically intact does not establish function. At minimum, the authors should select two enzymes from their test set and experimentally validate activity. For example, I could imagine that Taq polymerase mutants could be cloned and tested in PCR assays for polymerase activity, fidelity, and thermostability. Similarly, you could take cellulase mutants and assay them for hydrolytic activity against cellulose or model substrates, with kinetic

constants (k_cat, K_M) reported relative to wild-type. Even partial retention of activity in mutants with <30% sequence identity would strongly substantiate the approach.

We appreciate this suggestion and agree that experimental validation would be a valuable next step. We acknowledge that preserving catalytic-site geometry is not logically equivalent to preserving function. However, the well-established principle in chemistry that local structure strongly correlates with function provides a solid basis for our approach: catalysis depends on the local chemical environment, so preserving this environment should increase the likelihood of retaining activity.

Conversely, variants that fail to preserve the catalytic-site structure are unlikely to be functional. Our method therefore biases the generation process toward sequences with a higher probability of yielding functional enzymes. As we describe in the manuscript, we envision this as a valuable step preceding experimental optimisation campaigns: generating diverse "seed" sequences that cover broad regions of sequence space while maintaining confidence in structural (and thus likely functional) integrity.

We look forward to a full experimental validation in future work and hope our publicly available sequence libraries will facilitate this. While various constraints (including funding) currently prevents us from performing new experiments, we now test our method against existing experimental data, and results presented in a new section 'Comparison to experimental data', see Fig. 10 and Supplementary Figs. S-11 and S-12. Here, we will outline briefly what we did, but details can be found in the new section in the manuscript. In practice, we use the experimental data gathered by Russ et al [Russ et al, “An evolution-based model for designing chorismate mutase enzymes”. Science, 369(6502):440–445, 2020] in their study of chorismate mutase, repeating their analysis. This enzyme, which has some highly conserved residues in the catalytic region, has a large number of variants across species. We take the 1,000 variants that conserve the catalytic site (compared to the one present in E.coli) and were designed using another machine learning approach, bmDCA, where sequences are sampled from a statistical model fitted to the full MSA of 1,259 natural AroQ homologs. Crucially, all these variants have been experimentally measured and already classified as functional vs non-functional. Their sequence identity (compared to the base sequence) span ~100% to 20%. For all these functional mutants, we measure their embedding energy as defined and calculated by our method, using the closest functional natural variant as the reference. We also generate 500 variants by randomly mutating the base variant while keeping the active region fixed (**not** guided by any energy function) spanning again sequence identities from 20% to 100%. In practice, we recover the same results as Russ et al, although using our embedding energy rather than the statistical energy arising from their model: low (embedding) energy is not a sufficient condition, but it is a **necessary** condition for functionality, with 98.7% of all functional variants showing embedding energies lower than 0.05, while still being up to 70% different in sequence from the original enzyme. By an appropriate choice of the temperature, our method can generate sequence with an average energy around any target value and thus we will be able to remove from the pool of potential candidates for downstream experimental validation those which will almost surely not be functional. This result, together with what we previously showed in the manuscript, also supports the interpretation of similarity in the geometry of the catalytic region with preservation of functionality, providing an interpretative key we believe is instead missing from other measures, such as the use of the log-likelyhood of the overall sequence.

3. The validation currently re-demonstrates that ESM-2 embeddings already encode structural coherence, as shown by the high correlation between embedding energy and RMSD (rho = 0.85–0.93). This is expected and does not establish that Monte Carlo contributes beyond what diffusion- or Gaussian-perturbation models can already achieve. To strengthen the work, the authors should provide quantitative evidence that their method explores sequence space more effectively (ie, higher entropy of sampled mutants at matched catalytic-site RMSD) or more efficiently (fewer sampling steps required for comparable results).

While it is known that ESM-2 embeddings encode some structural information (this was indeed the reason to use them to define our energy for sampling), we believe the strength of the correlation we observe, and the fact that a simple cosine similarity suffices to guide preservation of entire catalytic-site geometries in 3D, is a non-trivial and practically useful finding.

Regarding the comparison between Monte Carlo and diffusion-based approaches: it is well known that Monte Carlo sampling and diffusion processes can describe equivalent physics, the underlying probability distribution follows a Fokker-Planck equation, of which the diffusion equation is a special case. For this reason, it is not clear what the reviewer means when they say *“This is expected and does not establish that Monte Carlo contributes beyond what diffusion- or Gaussian-perturbation models can already achieve”*. It probably does not, since it described a similar process.
However, our sampling - unlike the algorithms doing diffusion in latent space previously cited - does diffusion directly in sequence space. In this regard, Monte Carlo sequence sampling, the way we propose it at least, seems to us a much simpler, intuitive and interpretable way of approaching the problem of traversing the sequence-embedding space, compared to latent space methods: a “bad” mutation will lead to a change in the structure of the catalytic site, and thus discarded.
It also provides several practical advantages. The first is explicit control over which residues are preserved during the generation process. The second is more subtle, but because our energy is bounded from below (corresponding to the embeddings of the original enzyme to mimic), the sampling comes with well-known mathematical guarantees:

(i) convergence to a Boltzmann distribution that exponentially favours low-energy (structurally conserved) sequences. In other words, once the system is equilibrated, all generated sequences span a specific range of energy, and thus structural similarity with the original WT, given the strong correlation we have shown between the two. This also means that, waiting enough time, we will generate **all** sequences in this energy range, regardless of how far in sequence space they are from the original enzyme, as this does not matter in the sampling process.

(ii) the ability to control the extent of exploration through a single, interpretable parameter — the temperature.

Unguided sampling / simple diffusion in latent space, including Gaussian perturbation, does not inherently provide these guarantees, or at least we are not aware of any previous publication showing this mathematical connection, which in our case is instead trivial.

4. The framing of the manuscript says that there is practical readiness for enzyme design -- this is just not the case. The authors should temper their claims and reframe the title accordingly. A more accurate title would be “Embedding-guided Monte Carlo sampling generates sequence-diverse enzyme variants with preserved catalytic-site geometry”. This makes clear that what is shown here is structural preservation in silico, not yet functional enzyme generation.

The current title “Mind the Gap: An Embedding Guide to Safely Travel in Sequence Space” was chosen to be intriguing, it hardly contains any claim. Specifically, the title does not claim to achieve functional enzyme generation, proved in an experimental setting. What our title highlights is that the ability to guide the search in sequence space by the use of embeddings can ensure that we ‘safely’ find variant sequences that recover the same (albeit *in silico*) properties, those being defined by the structure alone. While we already tried to stress this in the original manuscript - where we note we carefully never made the claim that the variants generated by our algorithm will be functional - we try to make this point even clearer in the revised manuscript by adding the following sentence: “We should stress, however, that while necessary (see also Section \ref{sec:experiments}), preservation of the local environment is still not sufficient to guarantee catalytic activity. ”

Reviewer #2: The manuscript from Wu et al. presents an interesting generative method using protein language models. The work proposes using an energy function built on ESM-2 per-token embeddings to explore the mutational landscape while preserving key structural properties (catalytic sites) of proteins. The approach is simple yet convincing, with theoretical foundations well-stated and demonstrated throughout the paper. The discussion opens to broader applications and developments that could lead to better landscape exploration. As such, I think the paper is of high quality and well-suited for publication.

However, I would like to highlight some potential improvements that I believe the authors should address:

Major Points:

1. **Baseline comparisons**: "pLMs encode knowledge of chemically conservative substitutions allowing them to distinguish between structure-benign and disruptive mutations" In my opinion this critical point highlight the need for a stronger comparison with baselines energy functions. The work would benefit from comparisons with baseline energy functions ranging from simple (BLOSUM, independent site modeling) to more complex (DCA-type models) to demonstrate that the language model truly adds value.

We thank the reviewer for raising this point, and we now provide exactly such an analysis, which clearly strengthens the message of our paper. As it can be observed in Fig.S6,S7 in the Supplementary Information, which reports both results from sampling using energies from a simple BLOSUM as well as DCA (as requested by the reviewer) sampling based on our energy, derived from the embedding similarity provided by a pLM, outperforms both these types of models. To be more accurate, we observe that for the same level of sequence similarity, structures obtained by our sampling have smaller distortion in the active site (as measured by the RMSD), while if we compare structures with the same level of distortion, our sequences have a much larger difference (as measured by the Hamming distance) from the original one, increasing diversity. We speculate that the reason for this result is that, through the self-attention mechanism, embeddings from pLM contain information about correlations between residues that go beyond what can be obtained using models based on pair-energies, as in DCA models, and thus obviously also beyond single-site energy like in BLOSUM.

2. **Coupling analysis**: Since the model only performs single-site mutations, I question its ability to "tunnel" through fitness valleys by mutating strongly coupled residues. Can the model handle mutations at positions with strong epistatic interactions? This could be analyzed by identifying coupled residues through structural analysis or DCA, then examining whether the model can mutate these positions and how this correlates with temperature. Without this capability, the model may be limited to neutral mutations.

This is indeed an interesting point. If two residues have strong epistatic interactions, a protocol allowing to only make a single mutation per MC step would reject most of the mutations on these residues, ***if*** these mutations changed the folding of the protected/immutable site. At the same time, we observe that by simply changing the mutations protocol to also allow more than a single mutation per MC step before evaluating the energy change, non-neutral mutations to strongly coupled residues would incur in little or no energy change, and thus with high acceptance (at least if they compensate each other).
We have now run such simulations but we see no qualitative difference in the results obtained when only single point mutations are allowed (see Supplementary Information, Figure S5). In this regard we notice that, neutral or not, mutations to strongly coupled residues occur without any penalty if they do

not disrupt the fold of the protected site / immutable site, even if completely disrupt the enzyme fold outside of it. In fact, we observe many mutants per enzyme having a largely different structure, while still conserving the local geometry of the immutable region.

While more advanced sampling methods (e.g., parallel tempering) would allow to use a single-point mutation protocol and still overcome the energy barrier related to non-neutral mutations, we notice that such tunneling through the energy would still be possible by allowing double (or, more generally, multiple) mutations before calculating the energy term. This is because non-neutral mutations that compensate each other would still be expected to occur with low energy cost and thus high probability. While the exact result will depend on how many residues are strongly coupled to the protected region, we notice that such a double-mutation protocol, did not show any qualitative difference in the probability distribution of the generated enzymes from our original protocol with single-point mutations only.

3. **Computational efficiency**: Better reporting of computational costs is needed. What rejection rates should we expect, and how long does a Markov chain take to reach convergence (in term of time/FLOPS) ?

Rejection rates are a function of both the sampling temperature (monotonically increasing with the inverse of the temperature). They would also depend on specifics of the sampling process, e.g., how many aminoacids are allowed to mutate per MC step. But rejection rates are not particularly important: in Monte Carlo, a high rejection rate is perfectly fine if it helps the system decorrelate faster, thus producing truly independent samples. A similar consideration can be applied to the time to equilibrate: this is not the best number to measure computational efficiency, because the system can reach equilibrium, and yet take a long time to generate new uncorrelated variants once in equilibrium. A better number to measure computational efficiency would be to calculate the correlation function of the Hamming distance and use it to quantify the correlation time. This quantity (but also the rejection rate and the time-to-equilibration) are system and sampling dependent and while they can be optimized, such optimization is outside the scope of this paper.

Having said that, we believe this point to be mute and not particularly important for any practical application, for the following reason: for all the enzymes we describe here (up to almost 900 residues, for Taq polymerase) we can generate a hundred or so variants in an hour, using standard computational equipment nowadays available with a simple laptop with a single GPU. If our approach took 1 minute instead of one hour, it would not change in any appreciable way its usability for downstream experimental applications. Thus, its computational efficiency is good enough that any reasonably interested person would be able to use it. We now stress this point in the new manuscript, where we add:

“First, the computational cost of our generative procedure is minimal, determined solely by the pLM's sequence-to-embedding transformation, and is easily handled by low-end computational hardware, including desktop machines. The efficiency of such an algorithm is best measured by the number of independent variants that can be generated for a given computational budget, and can in principle be optimised in a system-dependent fashion through careful choice of parameters and mutation protocol (e.g., allowing more than a single mutation per MC step). Without any such optimisation, for the system sizes considered here — up to approximately 900 amino acids in length — one can already generate over 100 independent variants in under one hour on a standard laptop GPU. This baseline performance can be further improved through more complex sampling schemes (e.g., parallel tempering), or by parallelising the mutation process and leveraging batch predictions with a Heat Bath acceptance method instead of Metropolis MC [Frenkel & Smit].”

4. **Structural diversity analysis**: Do unprotected residues converge systematically to similar structures? Figure 3 suggests a bit of variability. Can the model diversify structure while maintaining catalytic sites? Does it sometimes converge to structures found in other protein families (which can

also be an interesting thing)? FoldSeek analysis could help reveal whether the model discovers alternative folds with similar catalytic sites.

While this is an interesting point, we believe it merits a proper, larger scale analysis. Such an analysis is outside of the scope of our manuscript, mostly because it would dilute our key message: to present a sampling protocol, based on the information provided by a pLM, to provide a reliable way to produce candidate mutants that should more likely conserve the structure of a predefined part of an enzyme.

Minor Points:

1. **Structure prediction validation**: “To reduce the possibility of having generated adversarial sequencs that might mislead the protein folding algorithm, calculations using AlphaFold2 have also been performed. These results are provided in Supplementary Information and fully confirm all trend observed using ESMFold” Given ESMFold's higher susceptibility to adversarial examples, I recommend reporting AlphaFold2 results in the main text. If you need to rerun some computations, also consider next-generation folding methods (AlphaFold3, Boltz, or Chai).

It is not clear that ESMFold has higher susceptibility to adversarial examples and, in fact, it has been recently shown that ESMFold might actually better capture the effect of mutations, while AlphaFold over-stabilise original structures [Feldman et al, https://www.biorxiv.org/content/10.64898/2026.02.25.708002v1]. Regardless, we prefer to keep the data where it is and not further overload the main manuscript, especially considering the extensive additions we have now made to it in response to various requests. In any case, the AlphaFold data confirms our claims, and is still present and fully visible in the Supplementary Information for any reader who wishes to check this point.

2. **Energy function alternatives**: Has cosine embedding similarity been compared with Euclidean distance? Is there a justification for the cosine choice? Similarly, why not use a pseudo-likelihood function from ESM's language modeling head?

In very high-dimensional space, the distance between any two points starts to look roughly the same. In other words, when the number of dimensions increases, the Euclidean distance becomes less effective in distinguishing the difference between two embedding vectors, thus weakening the strength of the correlation that a variation in this distance carries. Additionally, the Euclidean distance makes more sense to tell apart vectors when their magnitude varies in an appreciable range, so that its variation can carry information. However, due to the underlying ESM2 architecture (in particular, the Layer Normalization), the per-aminoacid embeddings have roughly the same magnitude, centred around $\sqrt{d_h}$, where $d_h = 1280$ is the size of the hidden dimension in our model. Cosine similarity instead tends to maintain better contrast between "similar" and "dissimilar" items in high-dimensional manifolds, which is why we have used this specific similarity measure in our work.

Regarding using the pseudo-likelihood, at the time we simply did not consider to use this measure because we did not see a direct correlation between this quantity and the (dis)similarity between two local environments, which is what we wanted to use as an energy to guide our sampling, under the assumption that preserving the local geometry of the catalytic site should bias towards variants that preserve catalytic activity.
While we were already aware that previous work has shown how this log-likelyhood correlates with the general fitness of a sequence, we have now realised it has been used to also look at enzymes functionality. In the revised manuscript, we now show, by comparison to experimental data, that our cosine energy not only measure similarity in geometry, but can also be used to tell screen out non-functional enzymes. These results are presented in a new section “Comparison to experimental data”, see Fig.10 and 11. Here, we will outline briefly what we did, but details can be found in the new section in the manuscript. In practice, we use the experimental data gathered by Russ et al [Russ et al, “An evolution-based model for designing chorismate mutase enzymes”. Science,

369(6502):440–445, 2020] in their study of chorismate mutase, repeating their analysis. This enzyme, which has some highly conserved residues in the catalytic region, has a large number of variants across species. We take the 1,000 variants that conserve the catalytic site (compared to the one present in E.coli) designed using another machine learning approach, bmDCA, where sequences are sampled from a statistical model fitted to the full MSA of 1,259 natural AroQ homologs. Crucially, all these variants have been experimentally measured and already classified as functional vs non-functional. Their sequence identity (compared to the base sequence) span ~100% to 20%. For all these functional mutants, we measure their embedding energy as defined and calculated by our method, using the closest functional natural variant as the reference. We also generate 500 variants by randomly mutating the base variant while keeping the active region fixed (**not** guided by any energy function) spanning again sequence identities from 20% to 100%. In practice, we recover the same results as Russ et al, although using our embedding energy rather than the statistical energy arising from their model: low (embedding) energy is not a sufficient condition, but it is a **necessary** condition for functionality, with 98.7% of all functional variants showing embedding energies lower than 0.05, while still being up to 70% different in sequence from the original enzyme. By an appropriate choice of the temperature, our method can generate sequence with an average energy around any target value and thus we will be able to remove from the pool of potential candidates for downstream experimental validation those which will almost surely not be functional. This result, together with what we previously showed in the manuscript, also supports the interpretation of similarity in the geometry of the catalytic region with preservation of functionality, providing an interpretative key we believe is instead missing from other measures, such as the use of the log-likelyhood of the overall sequence.

3. **Model specifications**: Which ESM-2 model version is used? Is it vanilla or fine-tuned through ESMFold training? Would ESMFold-finetuned models behave differently?

In the revised manuscript, we have added that we use the 650M version of the ESM-2 model, i.e. esm2_t33_650M_UR50D. We consider this to be the ‘vanilla’ one, but we also note that to the best of our knowledge, ESM-2 is never fine-tuned when trained as ESMFold. More specifically, the representation trunk (i.e. ESM-2 weights) are kept frozen, while only the folding trunk’s weights are being updated as they are trained on the PDB.

4. **Page 2**: "through training, these models learn the complex joint probability distribution of sequence and structure (or sequence-structure-function)" - It seems bold to say that the distribution of structure is learned. This is merely an emerging ability from the training.

We thank the reviewer for this observation. We note that this is a standard claim made by the generative protein modelling papers we cite (e.g. Madani et al., 2023). In that work, sequences generated by ProGen (a model trained on protein sequences) were experimentally shown to fold into the correct structure and exhibit catalytic activity, providing direct evidence that the model captures the statistical relationship between sequence, structure, and function. Whether one describes this as "learning" or "emergence" is a matter of terminology; the generated sequences demonstrably reflect the joint distribution over sequence and structure, which is what our statement conveys.

5. **Page 3**: "masked language problem" - This should be "masked language modeling," The masked language modeling is more a training technic/objective than a problem.

We have changed this in the revised manuscript.

Reviewer #3: This manuscript presents a hybrid approach that combines protein language model (pLM) embeddings (from ESM) with Monte Carlo (MC) sampling to generate enzyme mutants that preserve the local environment of catalytic sites. The method allows the exploration of diverse sequence variants while maintaining the geometry of a conserved region. The authors also

emphasize that the MC framework provides interpretability and tunability (e.g., via the temperature parameter) in contrast to end-to-end deep learning generative methods.

The approach is conceptually elegant and the manuscript is well written. The idea of using an energy-based Monte Carlo sampling guided by pLM embeddings is interesting and timely, especially in the context of generative protein design. However, I have two main concerns that should be addressed to make the work suitable for publication in PLoS Computational Biology.

1.
The authors do not discuss a substantial body of previous work that employed Monte Carlo sampling for generative modeling in sequence space, particularly in the context of Direct Coupling Analysis (DCA) but also in Transformer-based architectures. These approaches are in many ways conceptually analogous to the present one: the sampling is performed in sequence space under a model-derived energy, and subsets of sites can be fixed to represent structural or functional constraints (for example, catalytic sites).

Although pLM-based models do not require a multiple sequence alignment (MSA), while DCA-based ones do, a discussion comparing these frameworks would be highly valuable. In particular, the authors should clarify under which conditions their approach is preferable or complementary to DCA-based sampling, and whether the absence of MSA information limits or enhances interpretability and performance.

Relevant references include:

1. De la Paz, J. A., Nartey, C. M., Yuvaraj, M., & Morcos, F. (2020). Epistatic contributions promote the unification of incompatible models of neutral molecular evolution. Proc. Natl. Acad. Sci., 117, 5873–5882.
2. Bisardi, M., Rodriguez-Rivas, J., Zamponi, F., & Weigt, M. (2022). Modeling sequence-space exploration and emergence of epistatic signals in protein evolution. Mol. Biol. Evol., 39, msab321.
3. Alvarez, S., Nartey, C., Mercado, N., & Morcos, F. (2022). Novel sequence space explored by functional proteins generated through computational evolution-based design. Biophys. J., 121, 45a.
4. Alvarez, S., Nartey, C. M., Mercado, N., de la Paz, J. A., Huseinbegovic, T., & Morcos, F. (2024). In vivo functional phenotypes from a computational epistatic model of evolution. Proc. Natl. Acad. Sci., 121, e2308895121.
5. Biswas, A., Choudhuri, I., Arnold, E., Lyumkis, D., Haldane, A., & Levy, R. M. (2024). Kinetic coevolutionary models predict the temporal emergence of HIV-1 resistance mutations under drug selection pressure. Proc. Natl. Acad. Sci., 121, e2316662121.
6. Di Bari, L., Bisardi, M., Cotogno, S., Weigt, M., & Zamponi, F. (2024). Emergent time scales of epistasis in protein evolution. Proc. Natl. Acad. Sci., 121, e2406807121.
7. Sgarbossa, Damiano, Umberto Lupo, and Anne-Florence Bitbol. "Generative power of a protein language model trained on multiple sequence alignments." Elife 12 (2023): e79854

A discussion situating the present work within this existing framework is necessary to properly assess its novelty and contribution.

We agree with the reviewer’s comment that we should have discussed more extensively the previous literature, in particular, to understand its novelty, and we reviewed the suggested citations, which we found very useful. We have now implemented several changes.

Also, in response to a similar question by Referee 2, in the revised version of the manuscript we now provide a comparison to baseline models with substitutions based both on a simple BLOSUM matrix, as well as a more complex DCA approach.

Similarly to what was found by Sgarbossa et al, we find that a pLM based method, at least for the cases we investigate, is superior in sampling mutant sequence space. More precisely, we observe that:
i) if one wants to fix the maximum RMSD acceptable, DCA methods lead to mutants with higher sequence similarity to the wild-type (that is, less sequence diversity).
ii) if one instead wants to achieve a given sequence diversity, the RMSD between the variants generated by our method and the wild type is smaller compared to those generated via DCA-based methods. In other words, our pLM based method seems to preserve the original structure better.

"we highlight that the work and the results we present in this manuscript are better understood in the context of a wealth of recent literature on epistatic signals [1-7], that is, the context dependence of protein mutations, and its use to understand proteins evolution and protein design. In particular, various works have shown how methods such as Direct Coupling Analysis can detect epistatic signals, generating a roadmap of allowed vs forbidden mutations. Such roadmap, which naturally emerges following proteins evolution [2], can be leveraged to generate functional protein mutants. For example, Alvarez et al [3,4] has shown how DCA can be used to generate new-to-nature protein sequences, and proved that the sequences designed using epistatic models are functional *in vivo*, while often exploring areas of sequence space that nature has not yet reached. Di Bari et al [6] have previously discussed that these signals can be used to understand the timescales of evolution. What Alvarez et al [3,4] implied, and this is confirmed by our work, the same signals can be used to speed up evolution in silico. We also want to highlight here that we are not the first to show the power of deep-learning and the transformer architecture in capturing epistatic signals. In fact, close to the spirit of our work, Sgarbossa et al [7] previously used MSA Transformer, a protein language model trained on Multiple Sequence Alignments, to go beyond the pair correlations learnt by DCA. This work also showed how such signals can be used to generate high-quality, diverse sequences that mimic the natural one. Similarly to our own observations, as previously described, the MSA Transformer generated sequences that are more diverse compared to those generated via a DCA model, exploring new, viable areas of the fitness landscape. Our work extends these results in one important way. While more generally the fact that a protein language model purely trained on sequences alone, without the need of an MSA, can also capture co-evolutionary signals was extensively discussed in the original paper presenting the ESM-2 pLM, we show here that these signals not only correlate more generally to sequence fitness and functionality, but that the learnt embeddings from which these signals are calculated can be used as a direct measure of structural similarity in the local geometry of a given amino acid, extending their potential use to other tasks. For example, here we use them to generate new sequences that preserve the geometric structure of specific regions. In fact, this also suggest that if epistatic signals can be captured by ESM-2, and similarly the embeddings strongly correlate to local geometry, then the reason why epistatic signals can be used to screen out non-functional variants is exactly because they provide an idea of what mutations can be made without affecting functionality."

2.The validation presented in the manuscript is limited to showing that the generated sequences preserve the predicted fold (using AlphaFold or ESMFold). While this is a useful sanity check, it is also expected, given that pLM embeddings already encode structural information. Without any experimental validation it is difficult to assess the practical utility of the proposed approach. The results could at least be checked against existing data, for instance a comparison of model-generated variants with known functional mutants Even a small-scale in silico functional benchmark, e.g., on known mutational fitness datasets, would allow to better assess the proposed method.

We agree with the reviewer on the need for additional validation, beyond protein folding algorithms, to strengthen our paper. In the revised manuscript, we add 2 additional tests: one, as suggested by the reviewer, using experimental data taken from the literature. For the other one, we use a computational

method that is completely orthogonal to information-based protein folding, more precisely, Molecular Dynamics.

We look forward to a full experimental validation in future work and hope our publicly available sequence libraries will facilitate this. While various constraints (including funding) currently prevent us from performing new experiments, we now test our method against existing experimental data, and results presented in a new section 'Comparison to experimental data', see Fig. 10 and Supplementary Figs. S-11 and S-12. Here, we will outline briefly what we did, but details can be found in the new section in the manuscript. In practice, we use the experimental data gathered by Russ et al [Russ et al, “An evolution-based model for designing chorismate mutase enzymes”. Science, 369(6502):440–445, 2020] in their study of chorismate mutase, repeating their analysis. This enzyme, which has some highly conserved residues in the catalytic region, has a large number of variants across species.

We take the 1,000 variants that conserve the catalytic site (compared to the one present in E.coli) designed using another machine learning approach, bmDCA, where sequences are sampled from a statistical model fitted to the full MSA of 1,259 natural AroQ homologs. Crucially, all these variants have been experimentally measured and already classified as functional vs non-functional. Their sequence identity (compared to the base sequence) span ~100% to 20%. For all these functional mutants, we measure their embedding energy as defined and calculated by our method, using the closest functional natural variant as the reference. We also generate 500 variants by randomly mutating the base variant while keeping the active region fixed (**not** guided by any energy function) spanning again sequence identities from 20% to 100%. In practice, we recover the same results as Russ et al, although using our embedding energy rather than the statistical energy arising from their model: low (embedding) energy is not a sufficient condition, but it is a **necessary** condition for functionality, with > 98.7% of all functional variants showing embedding energies lower than 0.05, while still being up to 70% different in sequence from the original enzyme. By an appropriate choice of the temperature, our method can generate sequence with an average energy around any target value and thus we will be able to remove from the pool of potential candidates for downstream experimental validation those which will almost surely not be functional. This result, together with what we previously showed in the manuscript, also supports the interpretation of similarity in the geometry of the catalytic region with preservation of functionality, providing an interpretative key we believe is instead missing from other measures, such as the use of the log-likelyhood of the overall sequence.

Additionally, as discussed above, we use Molecular Dynamics to simulate a random subset of the enzymes generated by our method. The results, briefly outlined here, are described in a new section “Low embedding energy variants further preserve the local dynamics of the wild-type”.
All mutants tested are dynamically stable on the timescales considered: they preserve their tertiary structure, do not unfold, and exhibit fluctuations around their mean positions indistinguishable from those of the original enzyme. Previous work [Merlicek et al, Ai. zymes–a modular platform for evolutionary enzyme design, Angewandte Chemie International Edition 64 (2025)] has shown that short 10ns trajectories can efficiently screen out unstable mutants; here, stability is maintained up to 100ns (50ns equilibration +50ns production), an order of magnitude longer. More importantly, we apply a more stringent test: we compare the root-mean-square fluctuations of the active site in each mutant against those of the wild type, using the wild-type active site positions as the reference. Remarkably, fluctuations for all active site residues remain on the same scale in both mutants and wild type. In practice, these simulations confirm that our generative procedure preserves not only the average fold of the active site but also its dynamics.
While conserved folding is demonstrated here only \textit{in silico}, the agreement between two orthogonal methods — an information-based folding algorithm and physics-based MD — makes it less likely that the generated sequences are adversarial examples that merely satisfy

the structural predictor. The preservation of both average structure and dynamics relative to a known functional wild type should further increase the probability that catalytic activity is retained. We do not, however, claim functionality directly: our method generates enzymes with preserved structure in a user-defined region, and we suggest that this bodes well for preservation of function, since loss of this structure — as measured by the embedding energy and corroborated by the experimental comparisons described above — almost certainly entails loss of activity.
