# Supplementary material for: Mind the gap: An embedding guide to safely travel in sequence space
Source: PLoS Comput Biol. 2026 Jul 9;22(7):e1014433. doi: 10.1371/journal.pcbi.1014433 (PMC13375127; doi:10.1371/journal.pcbi.1014433)
Supplement: Attachment — Submitted filename: Reply_MindTheGapReply_2ND_ROUND.pdf [file pcbi.1014433.s003.pdf]

Response to Reviewers’ comments - 2n round

We thank the Reviewers for the positive feedback and recognition of the extensive work done during the 1st round of reviews. In the following, we address the few remaining remarks raised.

Like in the first review, we address each of the reviewers' comments sequentially. Changes in the main text reported here are highlighted in red. For the referees’ convenience, we have attempted to provide self-contained explanations of all modifications and additions here. However, a more complete understanding of the changes made might require consulting the revised manuscript, of which a version with all changes highlighted in red has also been provided.

==

Reviewers' comments:

==

Reviewer #1:

“I thank the authors for the revision. The new DCA/BLOSUM comparison and the chorismate mutase analysis are good improvements, and I do accept the authors' position that a head-to-head benchmark against latent-space generative models is out of scope. However, two issues from my original review remain inadequately addressed, and one new issue arises from the chorismate mutase analysis. These should be fixed before acceptance.

We thank the reviewer for recognising the additional work made in the first review in response to their comment. Additional comments are discussed below.

1. The current revision still does not situate the method within the body of work on sampling/perturbing pLM embeddings. Yes, the tasks are different, but pLM sampling The authors' Fokker–Planck argument that MC and diffusion "describe equivalent physics" actually strengthens the case for discussing these methods (not for omitting them). The authors should provide a dedicated paragraph in Related Work that (i) cites these methods, (ii) clearly states what task each was designed for, and (iii) articulates concretely what the present method offers that they do not (i.e., explicit residue-level constraints, Boltzmann-distribution guarantees).”

While we agree that the requested comparison might be interesting in general, the focus of our paper is the generation of enzyme variants, not to review all types of generative approaches for protein design. We reiterate that latent space embedding sampling/perturbation has not been used, to the best of our knowledge, for generation of enzyme mimics, only for peptide-based binder design. The answer to the question of whether latent-space perturbation method would work when one wants to mimic enzymes while preserving specific structural pockets is unclear, not trivial, and deserves its own study. Making a comparison here would simply distract

from the focus of our paper, and potentially suggest that a yet-to-be-tested approach would similarly work, which we simply do not know.

In this regard, we point out that after the first round of reviews, we did add a discussion of other generative methods in much more detail, but have done so discussing methods which were directly applied to enzyme generation. We do not think this discussion should be extended to other methods not tested on this problem.

“*2. I noticed that the new Figure 10 reports that 98.7% of functional variants have embedding energy < 0.05. This is sensitivity only, right? The actionable question for anyone using the energy as a pre-experimental filter is: what fraction of non-functional variants also fall below 0.05? Without that number, the "necessary condition" claim is not quantitatively useful. A filter that retains 98.7% of functional variants is worthless if it also retains 95% of non-functional ones. Please add a confusion matrix (or ROC curve) over the full Russ et al. dataset at the chosen threshold, and report precision, recall, and AUC. Also clarify the choice of "closest functional natural variant" as reference, which risks circularity if the reference is itself drawn from the labeled set.”*

We thank the reviewer for raising this point, as the analysis performed made us better understand our results. As requested, we now added the confusion matrix and ROC curve in Supplementary Information Section H (Fig. S-13).

The results of this analysis are:

Precision 50.2%, 95% CI [0.469, 0.534]
Recall 98.7%, 95% CI $[0.976, 0.997]
AUC 0.796, 95\% bootstrap CI [0.768, 0.823].
False-positive rate 64.9%

We notice that the false-positive rate and correlated quantities can probably be improved by a few percent without much impact on the false negatives using a different threshold, as visible from the ROC.

Regarding the last question, the "closest functional natural variant" is the natural sequence that was measured as functional in the same assay with the closest sequence alignment. The labelled set considered in our analysis only contains **synthetic** variants and does not include any of the natural variants used as reference for the embedding energy calculation, so no circularity is present. To make it clearer across that section, we changed the nomenclature from "designed variant" to "synthetic variant"

“*3. I asked for quantitative evidence that MC explores sequence space more*

effectively or efficiently than alternatives (entropy at matched RMSD, or step-count to comparable diversity). The authors responded with a theoretical argument about Boltzmann convergence. The DCA/BLOSUM comparison in the SI partially addresses this — please surface those numbers (entropy or sequence-diversity at matched catalytic-site RMSD) explicitly in the main text where concern #3 is relevant, rather than leaving it to the SI."

As per the reviewer's request, we have surfaced the numbers to the main text while keeping the related figures in the SI to avoid cluttering the already dense main text.

Quantitatively, pLM-guided sampling reaches sequence identities as low as ~20-30 % while keeping the protected-site RMSD at or below ~0.3A, whereas DCA- and BLOSUM-guided sampling remain confined to ~70-90 % sequence identity at comparable RMSDs and, when pushed to matched levels of sequence diversity, incur distortions exceeding ~1 A for DCA and reaching several A for BLOSUM (Figs S7 and S6).

===

"Reviewer #2: I thank the authors for their thorough response to my comments. The original manuscript was already strong, and the revisions have further improved it. The new baseline comparisons against BLOSUM and DCA convincingly address my main concern. The added multi-mutation analysis addresses the epistasis question reasonably. Finally, the clarifications brought to some technical aspects are also satisfactory. I have no further comments and recommend acceptance."

We thank the reviewer for their kind words. They seem satisfied so nothing to add here.

===

"Reviewer #3: I would like to commend the authors for their extensive efforts in addressing the various concerns raised during the initial round of review. The manuscript has been significantly strengthened by the additional analyses and discussions. I believe the paper is now close to being suitable for publication in PLOS Computational Biology, subject to minor revisions to temper a few overstrained statements introduced in the author responses and the revised text."

We thank the reviewer for their kind words and for recognising our extensive efforts in the first round of review. Below, we address their final requests/concerns.

"Specifically, I urge the authors to tone down the following claims:

1. In their response letter, the authors state:
‘Another advantage is that by using an energy definition that is bounded by below to do MC sampling mathematically implies very well known convergence results and guarantees full sampling of all the relevant structures.’

While this statement is theoretically accurate in the asymptotic long time limit, predicting the mixing time of a Markov chain in a high-dimensional sequence space is notoriously difficult. In concrete, practical applications, one never truly knows how long the Monte Carlo simulation will take to achieve a proper, representative sampling of the landscape. I request that the authors rephrase or qualify this claim to acknowledge the practical limitations imposed by mixing times in high-dimensional spaces.”

We thank the reviewer for the comment and agree that the original wording conflated asymptotic convergence with practical guarantees. We have qualified the claim in two places in the manuscript.

First, in the Methods section, the original text read:

OLD: Under very mild assumptions, statistical mechanics thus guarantees that our algorithm will sample a region minimising the free-energy $F(T) = E - TS(E)$ [...]

we have now rephrased it as:

NEW: "Under very mild assumptions, statistical mechanics thus implies that, in the long-time limit, our algorithm samples a region minimising the free-energy $F(T) = E - TS(E)$ [...]"

Second, and more substantively, in the Discussion section we have rewritten the paragraph that opened with "Beyond practical advantages, our method benefits from well-established theoretical guarantees [...]" to explicitly acknowledge the asymptotic nature of the convergence results and the practical limitations imposed by finite mixing times in high-dimensional sequence space. The paragraph now reads:

‘Beyond practical advantages, our method rests on a well-understood theoretical foundation. By employing a decades-old MC sampling method, we inherit its asymptotic convergence properties: in the long-time limit, the Markov chain samples a Boltzmann distribution associated with our energy function, favouring low-energy (structurally conserved) mutants with exponentially higher probability than high-energy ones. **We stress that these are asymptotic guarantees: as for any Markov chain in a high-dimensional sequence space, the mixing time is not knowable *a priori*, and finite-length runs may under-sample distant regions. In practice, we monitor energy trajectories to diagnose stationarity Fig.6 and find**

that, for the systems considered here, mutants with low sequence identity to the wild type are reached on accessible timescales, though we make no claim of exhaustive coverage of the relevant sequence space.'

“2. In the revised manuscript, the authors highlight:
"We highlight here that our method outperforms standard approaches for in silico directed evolution based on point or pairwise substitution correlations derived from Multiple Sequence Alignment, namely, sampling guided by BLOSUM-derived substitution energies or by Potts models fitted via mean-field Direct Coupling Analysis (see the Supplementary Information)."

The proposed method is certainly a promising alternative to existing frameworks and demonstrates interesting, specific advantages. However, claiming outright superiority over established approaches is premature without direct experimental validation. The authors should tone down assertions of "outperforming" other models until the method has been rigorously validated against wet-lab experiments.

For perspective, the authors should look at very recent work where various DCA variants were systematically benchmarked against experimental data and a variety of quantitative metrics were evaluated:

https://www.biorxiv.org/content/10.64898/2026.04.06.716859v1.abstract
https://arxiv.org/pdf/2605.03578

Metrics similar to those established in these benchmarks should be systematically compared before a definitive claim of superiority can be justified. Framing the method as a promising alternative with distinct structural preservation benefits would be more appropriate and scientifically rigorous.”

We thank the reviewer for this point and for the two references. We agree that our SI comparison is purely structural and does not warrant a generic claim of "outperformance". We have accordingly toned down the relevant wording throughout the main text and SI (please see the marked-up diff, as these comments are short/scattered and difficult to appreciate without the full context of the sentence). We have also added a sentence in the Discussion alongside the existing DCA/epistasis paragraph that acknowledges the two cited works and flags a head-to-head experimental fitness benchmark against these next-generation DCA variants as a natural extension that is out of scope for the present revision.

NEW: 'In a similar vein, recent extensions of the DCA framework have been shown, with experimental validation, to produce more diverse functional variants than the standard formulation we benchmark against in the Supplementary Information ~\cite{netti-2026,chauveau-2026}; a head-to-head experimental fitness comparison

of our pLM-based sampler against these next-generation DCA variants is a natural -- but out-of-scope -- extension of the structural comparison provided here.’
