# Supplementary material for: Mind the gap: An embedding guide to safely travel in sequence space
Source: PLoS Comput Biol. 2026 Jul 9;22(7):e1014433. doi: 10.1371/journal.pcbi.1014433 (PMC13375127; doi:10.1371/journal.pcbi.1014433)
Supplement: Peer review history

# Peer review: Decision Letter 0

**DOI:** 10.1371/journal.pcbi.1014433.r001

**Authors:** Nir Ben-Tal, Martin Weigt

6 Oct 2025

PCOMPBIOL-D-25-01684

Mind the Gap: An Embedding Guide to Safely Travel in Sequence Space

PLOS Computational Biology

Dear Dr. Angioletti-Uberti,

Thank you for submitting your manuscript to PLOS Computational Biology. After careful consideration, we feel that it has merit but does not fully meet PLOS Computational Biology's publication criteria as it currently stands. Therefore, we invite you to submit a revised version of the manuscript that addresses the points raised during the review process.

Please submit your revised manuscript within 60 days Dec 06 2025 11:59PM. If you will need more time than this to complete your revisions, please reply to this message or contact the journal office at ploscompbiol@plos.org. When you're ready to submit your revision, log on to https://www.editorialmanager.com/pcompbiol/ and select the 'Submissions Needing Revision' folder to locate your manuscript file.

Please include the following items when submitting your revised manuscript:

* A rebuttal letter that responds to each point raised by the editor and reviewer(s). You should upload this letter as a separate file labeled 'Response to Reviewers'. This file does not need to include responses to formatting updates and technical items listed in the 'Journal Requirements' section below.

* A marked-up copy of your manuscript that highlights changes made to the original version. You should upload this as a separate file labeled 'Revised Manuscript with Track Changes'.

* An unmarked version of your revised paper without tracked changes. You should upload this as a separate file labeled 'Manuscript'.

If you would like to make changes to your financial disclosure, competing interests statement, or data availability statement, please make these updates within the submission form at the time of resubmission. Guidelines for resubmitting your figure files are available below the reviewer comments at the end of this letter

We look forward to receiving your revised manuscript.

Kind regards,

Martin Weigt

Guest Editor

PLOS Computational Biology

Nir Ben-Tal

Section Editor

PLOS Computational Biology

**Journal Requirements:**

1) Please ensure that the CRediT author contributions listed for every co-author are completed accurately and in full.

At this stage, the following Authors/Authors require contributions: Adam Wu, Quentin Trolliet, Abhinav Rajendran, Jakub Lála, and Stefano Angioletti-Uberti. Please ensure that the full contributions of each author are acknowledged in the "Add/Edit/Remove Authors" section of our submission form.

The list of CRediT author contributions may be found here: https://journals.plos.org/ploscompbiol/s/authorship#loc-author-contributions

2) We ask that a manuscript source file is provided at Revision. Please upload your manuscript file as a .doc, .docx, .rtf or .tex. If you are providing a .tex file, please upload it under the item type u2018LaTeX Source Fileu2019 and leave your .pdf version as the item type u2018Manuscriptu2019.

3) Please provide an Author Summary. This should appear in your manuscript between the Abstract (if applicable) and the Introduction, and should be 150-200 words long. The aim should be to make your findings accessible to a wide audience that includes both scientists and non-scientists. Sample summaries can be found on our website under Submission Guidelines:

https://journals.plos.org/ploscompbiol/s/submission-guidelines#loc-parts-of-a-submission

4) Your manuscript is missing the following section heading: Abstract. Please make sure that the section heading levels are clearly indicated in the manuscript text, and limit sub-sections to 3 heading levels. An outline of the required sections can be consulted in our submission guidelines here:

https://journals.plos.org/ploscompbiol/s/submission-guidelines#loc-parts-of-a-submission

5) Please upload all main figures as separate Figure files in .tif or .eps format. For more information about how to convert and format your figure files please see our guidelines:

https://journals.plos.org/ploscompbiol/s/figures

6) We notice that your supplementary Figures, Tables, and information are included in the manuscript file. Please remove them and upload them with the file type 'Supporting Information'. Please ensure that each Supporting Information file has a legend listed in the manuscript after the references list.

7) Your current Financial Disclosure states, "The author(s) received no specific funding for this work." However, your funding information on the submission form indicates receiving a fund.

Please amend your detailed Financial Disclosure statement. This is published with the article. It must therefore be completed in full sentences and contain the exact wording you wish to be published.

1) Please clarify all sources of financial support for your study. List the grants, grant numbers, and organizations that funded your study, including funding received from your institution. Please note that suppliers of material support, including research materials, should be recognized in the Acknowledgements section rather than in the Financial Disclosure

2) State the initials, alongside each funding source, of each author to receive each grant. For example: "This work was supported by the National Institutes of Health (####### to AM; ###### to CJ) and the National Science Foundation (###### to AM)."

3) State what role the funders took in the study. If the funders had no role in your study, please state: "The funders had no role in study design, data collection and analysis, decision to publish, or preparation of the manuscript."

4) If any authors received a salary from any of your funders, please state which authors and which funders.

Note:  Please ensure that the funders and grant numbers match between the Financial Disclosure field and the Funding Information tab in your submission form. Note that the funders must be provided in the same order in both places as well

8) Please modify your 'Competing Interests' statement in the online submission form and declare all competing interests beginning with the statement "I have read the journal's policy and the authors of this manuscript have the following competing interests:"

Note: If the reviewer comments include a recommendation to cite specific previously published works, please review and evaluate these publications to determine whether they are relevant and should be cited. There is no requirement to cite these works unless the editor has indicated otherwise.

**Reviewers' comments:**

Reviewer's Responses to Questions

Reviewer #1: In this paper, the authors propose a generative algorithm for enzyme design that combines residue-level embeddings from ESM-2 with a Monte Carlo sampling framework. Their key idea is to define an "energy" function based on embedding similarity at user-defined protected residues (i.e., catalytic site residues) and to accept or reject mutations according to how much the embeddings of these residues are perturbed. In this way, the algorithm is claimed to generate mutants that can differ drastically in global sequence identity (as low as 2–5% in Table S-2) while preserving the local geometry of the catalytic site with RMSD values consistently below 0.3 A (Figure 3). They demonstrate this across 13 enzymes of diverse classes, highlighting examples such as Taq polymerase (1TAQ), oxidoreductases (1A2J, 4M6K), cellulases (1EDG), and hydrolases (1UA7). Structural coherence of the generated enzymes is validated in silico by ESMFold and AlphaFold2, with correlations between embedding “energy” and structural RMSD (Spearman rho = 0.85–0.93) as well as embedding energy and pLDDT (rho = –0.85 to –0.94) reported in Table II. The authors emphasize that their approach is interpretable and produces large mutant libraries (12,500 sequences per enzyme) with preserved active site geometry for future experimental screening.

The paper makes a strong case for interpretability and for the ability to bias generative protein design towards the conservation of catalytic residues. However, for me, the claims of methodological advance are undermined by two central weaknesses. First, the work does not include any benchmarking against other sampling-based generative approaches for protein design, making it difficult to judge novelty or relative performance. Second, the validation is entirely computational and does not demonstrate that any of the generated enzymes are functional. For enzymes, structure is insufficient, and catalytic activity must be shown. Without addressing these two gaps, the manuscript does not yet provide evidence that this is a viable generative strategy for enzyme engineering.

Here are four concerns the authors need to address:

1. The authors need to add valid benchmarks. The obvious comparisons are latent diffusion on ESM-2 embeddings, including DiMA and AMP-Diffusion, as well as Gaussian perturbation of ESM-2 embeddings as done in PepPrCLIP for peptide design. The authors should compare their Monte Carlo sampling to these types of strategues on the same set of enzymes, reporting side-by-side results for catalytic-site RMSD, global RMSD, pLDDT distributions, sequence identity ranges, and sequence diversity metrics. Without this, it is impossible to tell whether the proposed method provides any advantage over existing latent generative frameworks.

2. Demonstrating that catalytic residues remain geometrically intact does not establish function. At minimum, the authors should select two enzymes from their test set and experimentally validate activity. For example, I could imaginme that Taq polymerase mutants could be cloned and tested in PCR assays for polymerase activity, fidelity, and thermostability. Similarly, you could take cellulase mutants and assay them for hydrolytic activity against cellulose or model substrates, with kinetic constants (k_cat, K_M) reported relative to wild-type. Even partial retention of activity in mutants with <30% sequence identity would strongly substantiate the approach.

3. The validation currently re-demonstrates that ESM-2 embeddings already encode structural coherence, as shown by the high correlation between embedding energy and RMSD (rho = 0.85–0.93). This is expected and does not establish that Monte Carlo contributes beyond what diffusion- or Gaussian-perturbation models can already achieve. To strengthen the work, the authors should provide quantitative evidence that their method explores sequence space more effectively (ie, higher entropy of sampled mutants at matched catalytic-site RMSD) or more efficiently (fewer sampling steps required for comparable results).

4. The framing of the manuscript says that there is practical readiness for enzyme design -- this is just not the case. The authors should temper their claims and reframe the title accordingly. A more accurate title would be “Embedding-guided Monte Carlo sampling generates sequence-diverse enzyme variants with preserved catalytic-site geometry”. This makes clear that what is shown here is structural preservation in silico, not yet functional enzyme generation.

Reviewer #2: The manuscript from Wu et al. presents an interesting generative method using protein language models. The work proposes using an energy function built on ESM-2 per-token embeddings to explore the mutational landscape while preserving key structural properties (catalytic sites) of proteins. The approach is simple yet convincing, with theoretical foundations well-stated and demonstrated throughout the paper. The discussion opens to broader applications and developments that could lead to better landscape exploration. As such, I think the paper is of high quality and well-suited for publication.

However, I would like to highlight some potential improvements that I believe the authors should address:

Major Points:

1. **Baseline comparisons**: "pLMs encode knowledge of chemically conservative substitutions allowing them to distinguish between structure-benign and disruptive mutations" In my opinion this critical point highlight the need for a stronger comparison with baselines energy functions. The work would benefit from comparisons with baseline energy functions ranging from simple (BLOSUM, independent site modeling) to more complex (DCA-type models) to demonstrate that the language model truly adds value.

2. **Coupling analysis**: Since the model only performs single-site mutations, I question its ability to "tunnel" through fitness valleys by mutating strongly coupled residues. Can the model handle mutations at positions with strong epistatic interactions? This could be analyzed by identifying coupled residues through structural analysis or DCA, then examining whether the model can mutate these positions and how this correlates with temperature. Without this capability, the model may be limited to neutral mutations.

3. **Computational efficiency**: Better reporting of computational costs is needed. What rejection rates should we expect, and how long does a Markov chain take to reach convergence (in term of time/FLOPS) ?

4. **Structural diversity analysis**: Do unprotected residues converge systematically to similar structures? Figure 3 suggests a bit of variability. Can the model diversify structure while maintaining catalytic sites? Does it sometimes converge to structures found in other protein families (which can also be an interesting thing)? FoldSeek analysis could help reveal whether the model discovers alternative folds with similar catalytic sites.

Minor Points:

1. **Structure prediction validation**: “To reduce the possibility of having generated adversarial sequencs that might mislead the protein folding algorithm, calculations using AlphaFold2 have also been performed. These results are provided in Supplementary Information and fully confirm all trend observed using ESMFold” Given ESMFold's higher susceptibility to adversarial examples, I recommend reporting AlphaFold2 results in the main text. If you need to rerun some computations, also consider next-generation folding methods (AlphaFold3, Boltz, or Chai).

2. **Energy function alternatives**: Has cosine embedding similarity been compared with Euclidean distance? Is there a justification for the cosine choice? Similarly, why not use a pseudo-likelihood function from ESM's language modeling head?

3. **Model specifications**: Which ESM-2 model version is used? Is it vanilla or fine-tuned through ESMFold training? Would ESMFold-finetuned models behave differently?

4. **Page 2**: "through training, these models learn the complex joint probability distribution of sequence and structure (or sequence-structure-function)" - It seems bold to say that the distribution of structure is learned. This is merely an emerging ability from the training.

5. **Page 3**: "masked language problem" - This should be "masked language modeling," The masked language modeling is more a training technic/objective than a problem.

Reviewer #3: This manuscript presents a hybrid approach that combines protein language model (pLM) embeddings (from ESM) with Monte Carlo (MC) sampling to generate enzyme mutants that preserve the local environment of catalytic sites. The method allows the exploration of diverse sequence variants while maintaining the geometry of a conserved region. The authors also emphasize that the MC framework provides interpretability and tunability (e.g., via the temperature parameter) in contrast to end-to-end deep learning generative methods.

The approach is conceptually elegant and the manuscript is well written. The idea of using an energy-based Monte Carlo sampling guided by pLM embeddings is interesting and timely, especially in the context of generative protein design. However, I have two main concerns that should be addressed to make the work suitable for publication in PLoS Computational Biology.

1.

The authors do not discuss a substantial body of previous work that employed Monte Carlo sampling for generative modeling in sequence space, particularly in the context of Direct Coupling Analysis (DCA) but also in Transformer-based architectures. These approaches are in many ways conceptually analogous to the present one: the sampling is performed in sequence space under a model-derived energy, and subsets of sites can be fixed to represent structural or functional constraints (for example, catalytic sites).

Although pLM-based models do not require a multiple sequence alignment (MSA), while DCA-based ones do, a discussion comparing these frameworks would be highly valuable. In particular, the authors should clarify under which conditions their approach is preferable or complementary to DCA-based sampling, and whether the absence of MSA information limits or enhances interpretability and performance.

Relevant references include:

1. De la Paz, J. A., Nartey, C. M., Yuvaraj, M., & Morcos, F. (2020). Epistatic contributions promote the unification of incompatible models of neutral molecular evolution. Proc. Natl. Acad. Sci., 117, 5873–5882.

2. Bisardi, M., Rodriguez-Rivas, J., Zamponi, F., & Weigt, M. (2022). Modeling sequence-space exploration and emergence of epistatic signals in protein evolution. Mol. Biol. Evol., 39, msab321.

3. Alvarez, S., Nartey, C., Mercado, N., & Morcos, F. (2022). Novel sequence space explored by functional proteins generated through computational evolution-based design. Biophys. J., 121, 45a.

4. Alvarez, S., Nartey, C. M., Mercado, N., de la Paz, J. A., Huseinbegovic, T., & Morcos, F. (2024). In vivo functional phenotypes from a computational epistatic model of evolution. Proc. Natl. Acad. Sci., 121, e2308895121.

5. Biswas, A., Choudhuri, I., Arnold, E., Lyumkis, D., Haldane, A., & Levy, R. M. (2024). Kinetic coevolutionary models predict the temporal emergence of HIV-1 resistance mutations under drug selection pressure. Proc. Natl. Acad. Sci., 121, e2316662121.

6. Di Bari, L., Bisardi, M., Cotogno, S., Weigt, M., & Zamponi, F. (2024). Emergent time scales of epistasis in protein evolution. Proc. Natl. Acad. Sci., 121, e2406807121.

7. Sgarbossa, Damiano, Umberto Lupo, and Anne-Florence Bitbol. "Generative power of a protein language model trained on multiple sequence alignments." Elife 12 (2023): e79854

A discussion situating the present work within this existing framework is necessary to properly assess its novelty and contribution.

2.

The validation presented in the manuscript is limited to showing that the generated sequences preserve the predicted fold (using AlphaFold or ESMFold). While this is a useful sanity check, it is also expected, given that pLM embeddings already encode structural information. Without any experimental validation it is difficult to assess the practical utility of the proposed approach. The results could at least be checked against existing data, for instance a comparison of model-generated variants with known functional mutants Even a small-scale in silico functional benchmark, e.g., on known mutational fitness datasets, would allow to better assess the proposed method.

In summary, this is an interesting and well-executed study that proposes a novel hybrid use of pLMs and Monte Carlo sampling for enzyme design. However, the manuscript in its current form overlooks key related literature and lacks convincing validation beyond structure prediction. Addressing these points would substantially improve the clarity, novelty, and impact of the work.

**********

**Have the authors made all data and (if applicable) computational code underlying the findings in their manuscript fully available?**

The PLOS Data policy requires authors to make all data and code underlying the findings described in their manuscript fully available without restriction, with rare exception (please refer to the Data Availability Statement in the manuscript PDF file). The data and code should be provided as part of the manuscript or its supporting information, or deposited to a public repository. For example, in addition to summary statistics, the data points behind means, medians and variance measures should be available. If there are restrictions on publicly sharing data or code —e.g. participant privacy or use of data from a third party—those must be specified.

Reviewer #1: Yes

Reviewer #2: Yes

Reviewer #3: Yes

**********

PLOS authors have the option to publish the peer review history of their article (what does this mean?). If published, this will include your full peer review and any attached files.

If you choose “no”, your identity will remain anonymous but your review may still be made public.

**Do you want your identity to be public for this peer review?**  For information about this choice, including consent withdrawal, please see our Privacy Policy.

Reviewer #1: **Yes:**  Pranam Chatterjee

Reviewer #2: No

Reviewer #3: No

[NOTE: If reviewer comments were submitted as an attachment file, they will be attached to this email and accessible via the submission site. Please log into your account, locate the manuscript record, and check for the action link "View Attachments". If this link does not appear, there are no attachment files.]

**Figure resubmission:**

While revising your submission, we strongly recommend that you use PLOS’s NAAS tool (https://ngplosjournals.pagemajik.ai/artanalysis) to test your figure files. NAAS can convert your figure files to the TIFF file type and meet basic requirements (such as print size, resolution), or provide you with a report on issues that do not meet our requirements and that NAAS cannot fix.

After uploading your figures to PLOS’s NAAS tool - https://ngplosjournals.pagemajik.ai/artanalysis, NAAS will process the files provided and display the results in the "Uploaded Files" section of the page as the processing is complete. If the uploaded figures meet our requirements (or NAAS is able to fix the files to meet our requirements), the figure will be marked as "fixed" above. If NAAS is unable to fix the files, a red "failed" label will appear above. When NAAS has confirmed that the figure files meet our requirements, please download the file via the download option, and include these NAAS processed figure files when submitting your revised manuscript.

**Reproducibility:**

To enhance the reproducibility of your results, we recommend that authors of applicable studies deposit laboratory protocols in protocols.io, where a protocol can be assigned its own identifier (DOI) such that it can be cited independently in the future. Additionally, PLOS ONE offers an option to publish peer-reviewed clinical study protocols. Read more information on sharing protocols at https://plos.org/protocols?utm_medium=editorial-email&utm_source=authorletters&utm_campaign=protocols

# Author comment: Author response to Decision Letter 1

**DOI:** 10.1371/journal.pcbi.1014433.r002

16 Apr 2026

Attachment Submitted filename: MindTheGapReply.pdf

# Peer review: Decision Letter 1

**DOI:** 10.1371/journal.pcbi.1014433.r003

**Authors:** Nir Ben-Tal, Martin Weigt

18 May 2026

PCOMPBIOL-D-25-01684R1

Mind the Gap: An Embedding Guide to Safely Travel in Sequence Space

PLOS Computational Biology

Dear Dr. Angioletti-Uberti,

Thank you for submitting your manuscript to PLOS Computational Biology. After careful consideration, we feel that it has merit but does not fully meet PLOS Computational Biology's publication criteria as it currently stands. Therefore, we invite you to submit a revised version of the manuscript that addresses the points raised during the review process.

Please submit your revised manuscript by Jul 18 2026 11:59PM. If you will need more time than this to complete your revisions, please reply to this message or contact the journal office at ploscompbiol@plos.org. When you're ready to submit your revision, log on to https://www.editorialmanager.com/pcompbiol/ and select the 'Submissions Needing Revision' folder to locate your manuscript file.

Please include the following items when submitting your revised manuscript:

* A letter that responds to each point raised by the editor and reviewer(s). You should upload this letter as a separate file labeled 'Response to Reviewers'. This file does not need to include responses to formatting updates and technical items listed in the 'Journal Requirements' section below.

* A marked-up copy of your manuscript that highlights changes made to the original version. You should upload this as a separate file labeled 'Revised Manuscript with Track Changes'.

* An unmarked version of your revised paper without tracked changes. You should upload this as a separate file labeled 'Manuscript'.

If you would like to make changes to your financial disclosure, competing interests statement, or data availability statement, please make these updates within the submission form at the time of resubmission. Guidelines for resubmitting your figure files are available below the reviewer comments at the end of this letter.

As the corresponding author, your ORCID iD is verified in the submission system and will appear in the published article. PLOS supports the use of ORCID, and we encourage all coauthors to register for an ORCID iD and use it as well. Please encourage your coauthors to verify their ORCID iD within the submission system before final acceptance, as unverified ORCID iDs will not appear in the published article. Only the individual author can complete the verification step; PLOS staff cannot verify ORCID iDs on behalf of authors.

We look forward to receiving your revised manuscript.

Kind regards,

Martin Weigt

Guest Editor

PLOS Computational Biology

Nir Ben-Tal

Section Editor

PLOS Computational Biology

**Additional Editor Comments:**

The reviewers acknowledge the valuable work done during the revision and are generically positive about publication in PLoS CB, but ask for further clarifications and raise some new questions related to the revision. If these points are addressed carefully by the authors, acceptance for publication seems probable.

**Journal Requirements:**

1) Please ensure that the CRediT author contributions listed for every co-author are completed accurately and in full.

At this stage, the following Authors/Authors require contributions: Adam Wu, Jakub Lála, Quentin Trolliet, Abhinav Rajendran, and Stefano Angioletti-Uberti. Please ensure that the full contributions of each author are acknowledged in the "Add/Edit/Remove Authors" section of our submission form.

The list of CRediT author contributions may be found here: https://journals.plos.org/ploscompbiol/s/authorship#loc-author-contributions

2) We have noticed that you have uploaded Supporting Information files, but you have not included a list of legends. Please add a full list of legends for your Supporting Information files after the references list.

3) Please amend your detailed Financial Disclosure statement. This is published with the article. It must therefore be completed in full sentences and contain the exact wording you wish to be published.

- State the initials, alongside each funding source, of each author to receive each grant. For example: "This work was supported by the National Institutes of Health (####### to AM; ###### to CJ) and the National Science Foundation (###### to AM)."

- State what role the funders took in the study. If the funders had no role in your study, please state: "The funders had no role in study design, data collection and analysis, decision to publish, or preparation of the manuscript.".

If you did not receive any funding for this study, please simply state: u201cThe authors received no specific funding for this work.u201d

**Reviewers' comments:**

Reviewer's Responses to Questions

**Comments to the Authors:**

**Please note here if the review is uploaded as an attachment.**

Reviewer #1: I thank the authors for the revision. The new DCA/BLOSUM comparison and the chorismate mutase analysis are good improvements, and I do accept the authors' position that a head-to-head benchmark against latent-space generative models is out of scope. However, two issues from my original review remain inadequately addressed, and one new issue arises from the chorismate mutase analysis. These should be fixed before acceptance.

1. The current revision still does not situate the method within the body of work on sampling/perturbing pLM embeddings. Yes, the tasks are different, but pLM sampling The authors' Fokker–Planck argument that MC and diffusion "describe equivalent physics" actually strengthens the case for discussing these methods (not for omitting them). The authors should provide a dedicated paragraph in Related Work that (i) cites these methods, (ii) clearly states what task each was designed for, and (iii) articulates concretely what the present method offers that they do not (i.e., explicit residue-level constraints, Boltzmann-distribution guarantees).

2. I noticed that the new Figure 10 reports that 98.7% of functional variants have embedding energy < 0.05. This is sensitivity only, right? The actionable question for anyone using the energy as a pre-experimental filter is: what fraction of non-functional variants also fall below 0.05? Without that number, the "necessary condition" claim is not quantitatively useful. A filter that retains 98.7% of functional variants is worthless if it also retains 95% of non-functional ones. Please add a confusion matrix (or ROC curve) over the full Russ et al. dataset at the chosen threshold, and report precision, recall, and AUC. Also clarify the choice of "closest functional natural variant" as reference, which risks circularity if the reference is itself drawn from the labeled set.

3. I asked for quantitative evidence that MC explores sequence space more effectively or efficiently than alternatives (entropy at matched RMSD, or step-count to comparable diversity). The authors responded with a theoretical argument about Boltzmann convergence. The DCA/BLOSUM comparison in the SI partially addresses this — please surface those numbers (entropy or sequence-diversity at matched catalytic-site RMSD) explicitly in the main text where concern #3 is relevant, rather than leaving it to the SI.

Reviewer #2: I thank the authors for their thorough response to my comments. The original manuscript was already strong, and the revisions have further improved it. The new baseline comparisons against BLOSUM and DCA convincingly address my main concern. The added multi-mutation analysis addresses the epistasis question reasonably. Finally, the clarifications brought to some technical aspects are also satisfactory. I have no further comments and recommend acceptance.

Reviewer #3: I would like to commend the authors for their extensive efforts in addressing the various concerns raised during the initial round of review. The manuscript has been significantly strengthened by the additional analyses and discussions. I believe the paper is now close to being suitable for publication in PLOS Computational Biology, subject to minor revisions to temper a few overstrained statements introduced in the author responses and the revised text.

Specifically, I urge the authors to tone down the following claims:

1. In their response letter, the authors state:

"Another advantage is that by using an energy definition that is bounded by below to do MC sampling mathematically implies very well known convergence results and guarantees full sampling of all the relevant structures."

While this statement is theoretically accurate in the asymptotic long time limit, predicting the mixing time of a Markov chain in a high-dimensional sequence space is notoriously difficult. In concrete, practical applications, one never truly knows how long the Monte Carlo simulation will take to achieve a proper, representative sampling of the landscape. I request that the authors rephrase or qualify this claim to acknowledge the practical limitations imposed by mixing times in high-dimensional spaces.

2. In the revised manuscript, the authors highlight:

"We highlight here that our method outperforms standard approaches for in silico directed evolution based on point or pairwise substitution correlations derived from Multiple Sequence Alignment, namely, sampling guided by BLOSUM-derived substitution energies or by Potts models fitted via mean-field Direct Coupling Analysis (see the Supplementary Information)."

The proposed method is certainly a promising alternative to existing frameworks and demonstrates interesting, specific advantages. However, claiming outright superiority over established approaches is premature without direct experimental validation. The authors should tone down assertions of "outperforming" other models until the method has been rigorously validated against wet-lab experiments.

For perspective, the authors should look at very recent work where various DCA variants were systematically benchmarked against experimental data and a variety of quantitative metrics were evaluated:

https://www.biorxiv.org/content/10.64898/2026.04.06.716859v1.abstract

https://arxiv.org/pdf/2605.03578

Metrics similar to those established in these benchmarks should be systematically compared before a definitive claim of superiority can be justified. Framing the method as a promising alternative with distinct structural preservation benefits would be more appropriate and scientifically rigorous.

**********

**Have the authors made all data and (if applicable) computational code underlying the findings in their manuscript fully available?**

The PLOS Data policy requires authors to make all data and code underlying the findings described in their manuscript fully available without restriction, with rare exception (please refer to the Data Availability Statement in the manuscript PDF file). The data and code should be provided as part of the manuscript or its supporting information, or deposited to a public repository. For example, in addition to summary statistics, the data points behind means, medians and variance measures should be available. If there are restrictions on publicly sharing data or code —e.g. participant privacy or use of data from a third party—those must be specified.

Reviewer #1: Yes

Reviewer #2: Yes

Reviewer #3: Yes

**********

PLOS authors have the option to publish the peer review history of their article (what does this mean?). If published, this will include your full peer review and any attached files.

If you choose “no”, your identity will remain anonymous but your review may still be made public.

**Do you want your identity to be public for this peer review?**  For information about this choice, including consent withdrawal, please see our Privacy Policy.

Reviewer #1: No

Reviewer #2: No

Reviewer #3: No

[NOTE: If reviewer comments were submitted as an attachment file, they will be attached to this email and accessible via the submission site. Please log into your account, locate the manuscript record, and check for the action link "View Attachments". If this link does not appear, there are no attachment files.]

**Figure resubmission:**

-->While revising your submission, we strongly recommend that you use PLOS’s NAAS tool (https://ngplosjournals.pagemajik.ai/artanalysis) to test your figure files. NAAS can convert your figure files to the TIFF file type and meet basic requirements (such as print size, resolution), or provide you with a report on issues that do not meet our requirements and that NAAS cannot fix.

After uploading your figures to PLOS’s NAAS tool - https://ngplosjournals.pagemajik.ai/artanalysis, NAAS will process the files provided and display the results in the "Uploaded Files" section of the page as the processing is complete. If the uploaded figures meet our requirements (or NAAS is able to fix the files to meet our requirements), the figure will be marked as "fixed" above. If NAAS is unable to fix the files, a red "failed" label will appear above. When NAAS has confirmed that the figure files meet our requirements, please download the file via the download option, and include these NAAS processed figure files when submitting your revised manuscript.

**Reproducibility:**

To enhance the reproducibility of your results, we recommend that authors of applicable studies deposit laboratory protocols in protocols.io, where a protocol can be assigned its own identifier (DOI) such that it can be cited independently in the future. Additionally, PLOS ONE offers an option to publish peer-reviewed clinical study protocols. Read more information on sharing protocols at https://plos.org/protocols?utm_medium=editorial-email&utm_source=authorletters&utm_campaign=protocols

# Author comment: Author response to Decision Letter 2

**DOI:** 10.1371/journal.pcbi.1014433.r004

10 Jun 2026

Attachment Submitted filename: Reply_MindTheGapReply_2ND_ROUND.pdf

# Editor report: Decision Letter 2

**DOI:** 10.1371/journal.pcbi.1014433.r005

**Authors:** Nir Ben-Tal, Martin Weigt

11 Jun 2026

Dear Dr Angioletti-Uberti,

We are pleased to inform you that your manuscript 'Mind the Gap: An Embedding Guide to Safely Travel in Sequence Space' has been provisionally accepted for publication in PLOS Computational Biology.

Before your manuscript can be formally accepted you will need to complete some formatting changes, which you will receive in a follow up email. A member of our team will be in touch with a set of requests.

Please note that your manuscript will not be scheduled for publication until you have made the required changes, so a swift response is appreciated.

IMPORTANT: The editorial review process is now complete. PLOS will only permit corrections to spelling, formatting or significant scientific errors from this point onwards. Requests for major changes, or any which affect the scientific understanding of your work, will cause delays to the publication date of your manuscript.

Should you, your institution's press office or the journal office choose to press release your paper, you will automatically be opted out of early publication. We ask that you notify us now if you or your institution is planning to press release the article. All press must be co-ordinated with PLOS.

Thank you again for supporting Open Access publishing; we are looking forward to publishing your work in PLOS Computational Biology.

Best regards,

Martin Weigt

Guest Editor

PLOS Computational Biology

Nir Ben-Tal

Section Editor

PLOS Computational Biology

***********************************************************

# Editor report: Acceptance letter

**DOI:** 10.1371/journal.pcbi.1014433.r006

**Authors:** Nir Ben-Tal, Martin Weigt

PCOMPBIOL-D-25-01684R2

Mind the Gap: An Embedding Guide to Safely Travel in Sequence Space

Dear Dr Angioletti-Uberti,

I am pleased to inform you that your manuscript has been formally accepted for publication in PLOS Computational Biology. Your manuscript is now with our production department and you will be notified of the publication date in due course.

The corresponding author will soon be receiving a typeset proof for review, to ensure errors have not been introduced during production. Please review the PDF proof of your manuscript carefully, as this is the last chance to correct any errors. Please note that major changes, or those which affect the scientific understanding of the work, will likely cause delays to the publication date of your manuscript.

Soon after your final files are uploaded, unless you have opted out, the early version of your manuscript will be published online. The date of the early version will be your article's publication date. The final article will be published to the same URL, and all versions of the paper will be accessible to readers.

For Research, Software, and Methods articles, you will receive an invoice from PLOS for your publication fee after your manuscript has reached the completed accept phase. If you receive an email requesting payment before acceptance or for any other service, this may be a phishing scheme. Learn how to identify phishing emails and protect your accounts at https://explore.plos.org/phishing.

Thank you again for supporting PLOS Computational Biology and open-access publishing. We are looking forward to publishing your work!

With kind regards,

Sharmila Kamatchi

PLOS Computational Biology | Carlyle House, Carlyle Road, Cambridge CB4 3DN | United Kingdom ploscompbiol@plos.org | Phone +44 (0) 1223-442824 | ploscompbiol.org | @PLOSCompBiol
